# A Data-Driven Semi-Quantitative Framework for Comparative Screening and Tiering of Cellulose Nanocrystal Isolation Pathways: Implications for Process Assessment and Future Optimization

**DOI:** 10.3390/molecules31173042

**Published:** 2026-08-29

**Authors:** Atefeh Esmaeili, Farzin Shabani, Mohammad A. Al-Ghouti

**Affiliations:** Department of Biological and Environmental Sciences, College of Arts and Sciences, Qatar University, Doha P.O. Box 2713, Qatar; atefeheh.esmaeili@qu.edu.qa (A.E.); fshabani@qu.edu.qa (F.SH.)

**Keywords:** nanocrystalline cellulose, lignocellulosic biomass pretreatment, alkaline treatment, bleaching treatment, cellulose acid hydrolysis, process burden assessment

## Abstract

Cellulose nanocrystals (CNCs) are lignocellulose-derived nanomaterials valued across multiple industries due to their low toxicity and density, high mechanical strength and surface area, biodegradability, and potential as renewable alternatives to fossil-based materials. However, CNC isolation remains highly variable, with no structured framework for comparing processing routes according to their integrated operational burden, defined here as the combined relative Labor, Waste, and Cost scores assigned to the reported processing conditions. This study presents a literature-derived, semi-quantitative Scoring and Tiering framework for comparing CNC isolation processes and their main stages (alkaline treatment, bleaching, and acid hydrolysis), based on the relative operational burden reported across 140 studies. The burden was assessed across Labor, Waste, and Cost categories. The proposed framework enables comparison at process, stage, and category levels. The Waste category contributed most strongly to the calculated scores, whereas Labor contributed less. Bleaching showed the greatest variability, while alkaline treatments were more standardized. The proposed framework provides a transparent, literature-derived, semi-quantitative tool for structured screening, comparison, and prioritization of CNC isolation pathways based on their relative operational burden. Its Tiers are intended as comparative indicators rather than absolute measures of sustainability, scalability, or industrial performance. Future laboratory- and pilot-scale validation incorporating feedstock variability, standardized process-balance data, and integration of the Process Tier with CNC quality metrics could enable a more comprehensive evaluation of CNC isolation pathways.

## 1. Introduction

Environmentally friendly principles, which are gaining increasing attention nowadays, reinforce the use of renewable natural resources such as lignocellulosic biomass for different industrial applications [1]. Lignocellulose, produced annually at around 1.3 × 10^10^ metric tons [2], is composed mainly of cellulose, hemicellulose, and lignin [3,4,5]. Cellulose is the most widespread natural polymer [6,7], made of glucose units, forming a hemi-crystalline chain structure that shapes plant cell walls [8,9,10]. Cellulose chains are stabilized by hydrogen bonds and van der Waals forces [7,11] to form microfibrils, which are surrounded by hemicellulose and lignin [12,13,14]. Being biodegradable, renewable, inexpensive, and available in plants and agricultural/industrial wastes makes cellulose a valuable alternative to fossil-fuel-based polymers [1,15,16]. Accordingly, cellulose nanocrystals (CNCs), also known as nanocrystalline cellulose and cellulose nano-whiskers [1,17], have drawn increasing attention due to high surface area and crystallinity, low toxicity, low density, high mechanical strength, biodegradability [18,19,20], and broad applicability across pharmaceutical, materials, energy, environmental, packaging, and chemical industries [6,16,21].

CNCs are typically isolated from lignocellulosic feedstocks through chemical (particularly acid hydrolysis), mechanical, biological, or combined processes [4,15,22]. The chemical route, one of the most widely used methods, involves subjecting feedstocks to a series of chemical pretreatments, including alkaline and bleaching steps to dissolve non-cellulosic substances (extract pure cellulose), followed by acid hydrolysis of the cellulose to obtain CNCs [15,23,24,25,26,27]. The alkaline treatment removes non-cellulosic constituents such as hemicellulose while partially affecting lignin. Bleaching eliminates lignin and residual hemicellulose and whitens the cellulosic fibers [22,24,28,29]. Acid hydrolysis acts on the amorphous regions of the resulting cellulose to release crystalline nanostructures [25,29,30,31]. However, the three main chemical stages (alkaline and bleaching treatments, and acid hydrolysis) have been reported in the literature under highly variable experimental conditions, including various chemical types, reagent concentrations, processing temperatures, reaction times, numbers of cycles, and feedstocks. While CNC isolation from agricultural wastes aligns with circular economy and waste-to-value principles, the operational aspects of these processes, including environmental sustainability, cost-effectiveness, and labor intensity, remain largely unstandardized across reported procedures. Moreover, existing studies primarily focus on yield, crystallinity, or individual process optimization, with limited effort to systematically compare the overall processing routes in terms of their integrated operational burden. This lack of comparative assessment constrains the identification of efficient and scalable CNC isolation pathways.

Therefore, this study aims to address this gap by conducting a structured analytical review of CNC isolation studies employing the three main chemical stages (alkaline treatment, bleaching, and acid hydrolysis), compiling key experimental variables, and analyzing their empirical distribution ranges. Subsequently, a structured Scoring and Tiering framework was developed to classify CNC isolation stages and the overall process based on three key dimensions (categories): labor intensity (Labor), waste generation (Waste), and cost (Cost). The framework provides a literature-derived, semi-quantitative, multidimensional approach for comparing the relative operational burden of CNC isolation pathways at the stage, category, and overall-process levels. It is intended to support preliminary screening and the identification of relatively lower-burden options reported in the literature rather than to measure absolute sustainability, scalability, or industrial performance.

A key strength of the approach is its transparent integration of Labor, Waste, and Cost within a single framework while retaining separate stage- and category-level comparisons. The sensitivity analysis also provides evidence of internal robustness to plausible changes in the assigned weights and penalties. However, the framework is based on published data, author-defined dimensionless ordinal penalties, laboratory-scale chemical prices, and Tier boundaries established from empirical percentile distributions within the defined 140-study dataset. It does not fully incorporate feedstock variability, complete process balances, or CNC quality metrics and has not yet been externally validated using standardized laboratory-, pilot-, or industrial-scale data. These limitations define priorities for the framework’s future development and validation.

## 2. Experimental Section

### 2.1. Materials

All statistical analyses and visualizations throughout the study were conducted using R software (version 4.4.1; R Core Team, Vienna, Austria).

### 2.2. Methods

#### 2.2.1. Study Selection Criteria

This study was conducted as a structured analytical review rather than a systematic review. The literature-identification procedure was designed to construct a well-defined analytical dataset containing sufficiently detailed experimental information to characterize the operational conditions used during CNC isolation. Relevant studies published between 2015 and 2025 were identified through Google Scholar using CNC extraction and isolation search terms, supplemented by Harzing’s Publish or Perish software (Windows GUI version 8.12.4612.8838; Harzing.com) with Crossref as the underlying bibliographic source.

Studies were generally included when they reported sufficiently detailed experimental procedures for the CNC-isolation workflow, including alkaline treatment, bleaching treatment, and acid hydrolysis, and provided sufficient quantitative characterization of the resulting CNC product. Studies investigating a single treatment stage were eligible if sufficient quantitative information was reported for that stage. Following screening and consolidation of duplicate records, the final analytical dataset comprised 140 unique studies and 686 experimental trials. The complete literature-identification and selection procedure, including the exclusion criteria, is provided in the Supplementary Material. The included studies are listed in Supplementary Table S1, and the screening process is shown in Figure S1.

#### 2.2.2. Dataset Preparation

For clarity, a stage refers to one of the three main experimental stages involved in the entire CNC isolation process: alkaline treatment, bleaching treatment, or acid hydrolysis. An operational variable is a reported process parameter or experimental condition, such as chemical concentration, temperature, processing time, or number of cycles. The distribution range describes the observed values of an operational variable across the reviewed dataset, whereas an operational regime is a literature-derived interval for an operational variable, classified as Low, Moderate, or High for each chemical type. Operational intensity refers to the relative processing severity indicated by the Low, Moderate, or High regimes of individual operational variables or by their combined conditions. Operational burden refers to the combined penalty points within a category, stage, or overall CNC-isolation process. A penalty point is a dimensionless ordinal weight assigned within a scoring category, namely Labor, Waste, or Cost. A Tier is a final percentile-based class (Tier 1–3) derived from the operational burden at the category, stage, or overall-process level. These terms are used consistently throughout the manuscript.

A comprehensive dataset was compiled by extracting all data on the CNC isolation process from the reviewed literature. The data were classified into three stages and included detailed experimental conditions (operational variables), such as chemical type, chemical concentration, concentration unit, experimental temperature, operating time, and number of repeated cycles.

To harmonize the data across the dataset and enable a more reasonable comparison of concentrations within each chemical type, the concentrations of alkaline and bleaching chemicals were converted to molarity, and acid concentrations were converted to normality using the reported % units. The concentration unit conversion formulae are provided in Table 1. The factors 10, 100, and 1000 in Table 1 arise from dimensional unit conversion rather than data scaling. Division by 100 converts percentage values to fractions, while multiplication by 1000 converts concentrations expressed on a milliliter basis to a liter basis. For % *w*/*v* and % *v*/*v* concentrations, these conversions simplify to a net factor of 10. These calculations were used to express concentrations reported in different units on a common molarity or normality basis and therefore did not alter the relative variation among observations. In Table 1, *ρ* _pure_ denotes the density of the pure chemical, whereas *ρ* _solution_ refers to the density of the prepared chemical solution used in the experiment. Density values were obtained from standard chemistry references and relevant sources [32,33,34,35,36]. When the required density value was not directly available, it was estimated using linear interpolation or extrapolation. The complete data-extraction and harmonization procedures, the density-estimation formula, and the estimated density values are provided in the Supplementary Material and in Table S2.

#### 2.2.3. Data Analyses and Operational Regimes Computation

After normalizing chemical concentrations and harmonizing experimental durations (time), the data were cleaned prior to analysis. Statistical analyses, including tests of normality and skewness, were conducted, and numerical operational variables were visualized using histograms and density plots, followed by estimation of Kernel Density functions (Estimates; KDE). Correlation analyses among the operational variables (concentration, temperature, time, and cycle) were performed for each of the three CNC isolation stages, with |r| = 0.8 used as a prespecified screening threshold for very high pairwise correlation [37].

The distribution ranges of each operational variable were analyzed using Gaussian Mixture Models (GMMs) implemented in the Mclust R package [38] to identify distinct distributional patterns and potential subgroups within each chemical category and stage. GMMs with one to four components were fitted, and the Bayesian Information Criterion (BIC) was employed to select the optimum number of components and covariance structure. The identified mixture components were interpreted as operational regimes reflecting common experimental practices rather than as hidden physicochemical processes. For scoring purposes, mixture-model thresholds were combined with robust distribution-based safety checks to classify operational regimes as Low, Moderate, or High, representing increasing operational intensity. The complete statistical analyses, mixture-modeling procedure, threshold determination, fallback rules, and validity checks are provided in the Supplementary Material.

#### 2.2.4. Ranking Chemicals According to Price

Chemical prices were calculated based on the cost of preparing 1 L of solution for each chemical, corresponding to the literature-derived operational regimes for concentration. Chemical market prices were obtained from two reputable, widely recognized suppliers, Sigma-Aldrich [39] and Fisher Scientific [40] and converted to US dollars. The lowest comparable quoted unit price for each chemical was selected and used for further calculation. Reagent grade and stock purity were incorporated into the calculations through reagent purity correction using the listed stock concentration or purity. The resulting estimates therefore represent laboratory-scale catalog costs and should not be interpreted as bulk industrial prices.

Chemical costs were categorized using unified study-wide price categories based on the observed distribution of prices across all alkaline, bleaching, and acid treatments. To ensure consistent and conservative penalty assignment, each cost range was assigned to a single category using worst-case logic. The resulting price categories and corresponding penalty points were used later to develop the CNC Scoring and Tiering Rubric from a cost perspective. The selected chemicals, unified prices, calculation procedures, price categories, corresponding penalty points, and calculation examples are provided in Supplementary Material and Tables S3–S6.

#### 2.2.5. Ranking Chemicals According to Environmental Friendliness

To score the chemicals associated with each stage of CNC isolation from a waste perspective, apart from chemical concentrations that directly affect the waste generated, the environmental friendliness of chemicals should be taken into account. To do that, the chemicals were evaluated and compared on two aspects: (1) post-use environmental burden, especially after the chemicals enter wastewater streams (Table S7) and (2) toxicity and ecotoxicity of the chemicals or their spent persistent inorganic ions/residuals (Tables S8–S13). The chemicals were assigned penalty scores/points (severity rank values), with higher values/scores indicating greater environmental harm and lower values reflecting greater environmental friendliness. Eventually, the total score for each chemical across the evaluated parameters in the two aspects was calculated and used to rank the chemicals by environmental friendliness from a waste perspective (Table S14).

##### 2.2.5.1. Post-Use Environmental Burden in Wastewater Streams

Chemicals within each stage (alkaline chemicals, bleaching chemicals, and acids) were comparatively assessed according to their documented post-use effects in wastewater. The assessment considered persistence, scaling and corrosion, nutrient enrichment, formation of toxic by-products, and potential effects on wastewater infrastructure and receiving environments. Based on the evidence and comparative reasoning detailed in “Section 3.4.1”, chemicals within each group were ordered according to their relative post-use burden, with higher ordinal penalty scores being assigned to chemicals considered to impose a greater burden. The resulting rankings and their underlying considerations are summarized in Table S7. These scores represent relative rankings within the proposed rubric rather than measured or empirically calibrated environmental-impact values.

##### 2.2.5.2. Toxicity and Ecotoxicity of the Chemicals

Animal and environmental toxicity (ecotoxicity; aquatic toxicity) values for each chemical or its related mineral ions were primarily extracted from Material Safety Data Sheets (MSDSs), which compile data from reliable sources such as OECD (Organization for Economic Co-operation and Development) standard test guideline, the ECOTOX database of the U.S. Environmental Protection Agency (EPA), and the European Chemicals Agency (ECHA; EU REACH database). Published studies reporting relevant standard toxicological tests were also referred to where available. Guideline threshold values were retrieved from reports and official websites of the Environmental Protection Agency (EPA) and the World Health Organization (WHO). When multiple values were available from reliable sources, the lowest value was selected to ensure a conservative approach, while unverified data (data for which the original source was not specified) were used only when no reliable data were available. Similar toxicity tests or endpoints were compared across chemicals within each stage, and chemicals with lower toxicity values were assigned greater penalty points, indicating their relatively higher toxicity. Refer to Supplementary Material for more details. Results for chemical toxicity and ecotoxicity are shown in Tables S8–S11, with Table S14 summarizing all penalty points and the total points assigned to each chemical.

##### 2.2.5.3. Toxicity and Ecotoxicity of the Inorganic Ions

Toxicity and ecotoxicity of the inorganic ions derived from the chemicals involved in each of the three CNC isolation stages are summarized in Tables S12 and S13. Regarding the alkaline chemicals, sodium and potassium ions were used as the representative inorganic ions derived from sodium and potassium hydroxides, respectively. Regarding the bleaching chemicals, chlorine (Cl_2_) and free chlorine (ClO^−^ and HClO) were considered as inorganic ions derived from sodium hypochlorite, while chlorite (ClO_2_^−^) and chlorine dioxide (ClO_2_) were considered as sodium chlorite-originated ions. Chlorate (ClO_3_^−^) was considered a residual ion for both sodium hypochlorite and sodium chlorite, as it forms as a byproduct after the application of either bleaching agent and is also found as an impurity in both chemicals. For the mineral acids, sulfate (SO_4_^−2^), phosphate (PO_4_^−3^), and chloride (Cl^−1^) were considered as mineral ions derived from sulfuric acid, phosphoric acid, and hydrochloric acid, respectively.

##### 2.2.5.4. Overall Ranking of Chemicals from an Environmental Friendliness Perspective

To rank the chemicals by environmental friendliness for the Waste category, the total penalty points for each chemical, derived from Tables S7–S13, were summarized to obtain the final penalty points (Table S14). The chemicals related to each of the three stages of alkaline, bleach, and acid treatments were then ranked accordingly, with those having the smallest total points identified as the most environmentally friendly. The reasoning underlying the assigned penalty scores is detailed in “Section 3.4” and summarized in Tables S7–S13.

#### 2.2.6. CNC Scoring and Tiering Rubric: A Practical Guide

##### 2.2.6.1. Scoring Rules

Literature-derived distribution ranges for all operational variables categorized based on the chemical types involved in the three processing stages of the CNC isolation (alkali treatment, bleaching treatment, and acid hydrolysis), together with the results related to the chemicals’ environmental friendliness order and calculated price categories, were used to develop a Scoring and Tiering Rubric as a practical guide to evaluate CNC isolation methodologies, from three key perspectives or categories: labor intensity (Labor), waste generation (Waste), and cost (Cost). Therefore, the evaluation presented here considered the chemicals and consumables used in each CNC isolation processing stage, as well as the operational variables applied. The ultimate goal was to develop a practical, data-driven scoring system that classifies each of the three stages of the CNC isolation and the entire CNC isolation process, encompassing all stages, into three Tiers based on relative operational burden (Labor, Waste, and Cost) across the literature:

Tier 1: Lowest combined operational burden relative to other protocols.

Tier 2: Intermediate burden.

Tier 3: Highest combined operational burden.

This classification facilitates the identification of protocols with a relatively lower operational burden (considering cost, waste generation, and labor requirements) for the overall CNC isolation process and for each of its three processing stages, along with the total burden within each category.

The presented scoring framework is a data-driven and semi-quantitative screening tool. Operational regimes and final Tier cutoffs were derived from the empirical literature dataset, whereas individual penalty points are dimensionless ordinal weights that represent gradual increases in relative operational burden (overall operational intensity). They are not physical measurements or empirically calibrated effect sizes. Thus, an increase from 0.5 to 1.0 indicates a higher assigned burden but should not be interpreted as a measured twofold increase in Labor, Waste, Cost, environmental impact, or industrial performance. A common 0.5-point increment was used to preserve ordering and maintain consistent resolution across rubric components.

##### 2.2.6.2. Scoring Details: Labor Points

Labor points were assigned based on parameters that contribute to the process’s labor intensity, including treatment time, the number of stage cycles, additional adjustment steps, and the operation of high-pressure vessels. Time-derived penalties were based on chemical-specific operational regimes (Low, Moderate, and High) rather than absolute treatment duration to facilitate reasonable cross-chemical comparisons. The detailed criteria, operational regime thresholds, and corresponding penalty points used to calculate the Labor points for each stage are provided in the Supplementary Materials and summarized in Table 2.

##### 2.2.6.3. Scoring Details: Waste Points

Waste points were assigned according to the environmental friendliness of the chemicals, increasing from the most to the least environmentally friendly. Other contributing parameters included chemical concentration, experimental temperature, number of stage cycles, additional adjustment steps, and the operation of high-pressure vessels. Concentration- and temperature-derived penalties were based on chemical-specific operational regimes (Low, Moderate, and High) rather than absolute values to facilitate reasonable cross-chemical comparisons. The detailed criteria, operational regime thresholds, and corresponding penalty points used to calculate the Waste points for each stage are provided in the Supplementary Materials and summarized in Table 2.

##### 2.2.6.4. Scoring Details: Cost Points

Cost points were assigned relative to the final calculated price and the corresponding price categories of the chemicals, as explained in “Section 2.2.4”, increasing from the cheapest to the most expensive. Concentration-derived cost penalties were assigned using unified study-wide price categories, with standardized penalty increments applied consistently across the three chemical classes and their respective processing stages. Other parameters contributing to cost included experimental temperature, number of stage cycles, additional adjustment steps, and the use of high-pressure vessels. The detailed price subcategories, criteria, operational-regime thresholds, and corresponding penalty points used to calculate the Cost points for each stage are provided in the Supplementary Materials and summarized in Table 2.

##### 2.2.6.5. Tier Calculation: Stage-Level Total Tier Mapping

After assigning Labor, Waste, and Cost penalty points to each processing stage according to the parameters defined in the previous section, the total penalty points for each category can be calculated. In this regard, for each reviewed study, scores for Labor (L), Waste (W), and Cost (C) were calculated at the stage level by summing the assigned penalty points to obtain L_a_, W_a_, and C_a_ for the alkaline stage; L_b_, W_b_, and C_b_ for the bleaching stage; and L_h_, W_h_, and C_h_ for the acid hydrolysis stage.

Stage-Level Total Scores were then computed as the sum of the three category scores for each stage (e.g., total points of alkaline treatment (TP_a_) = L_a_ + W_a_ + C_a_). The empirical distribution of these Stage Total Scores across all 140 studies was used to define Tier boundaries based on percentile thresholds. Specifically, the 33rd percentile (P_33_) and 67th percentile (P_67_) were calculated from the entire dataset and used as cut-off values to define three Tiers: Tier 1 included values ≤ P_33_ (lowest 33% of observations), Tier 2 included values > P_33_ and ≤ P_67_ (middle 33%), and Tier 3 included values > P_67_ (highest 33%). This percentile-based approach preserves internal consistency within the scoring system and allows automatic recalibration as additional literature data are incorporated.

##### 2.2.6.6. Tier Calculation: Category-Level Total Tier Mapping

Category-Level Total Scores were calculated for each study by summing the scores within each category across the three stages (e.g., total labor points (L_Total_) = L_a_ + L_b_ + L_h_). Percentile-based thresholds (P_33_ and P_67_), derived from the empirical distribution of Category Total Scores in all 140 studies, were then used to define the Tier boundaries.

##### 2.2.6.7. Tier Calculation: Overall-Process Tier Mapping (Entire CNC Isolation Process)

This step determined the Tier classification for the entire CNC isolation process. Overall-Process Total Scores (TP_Overall_) were evaluated in two complementary ways: (i) by summing Total Category Scores across all stages (L_Total_ + W_Total_ + C_Total_) and (ii) by summing Total Stage Scores across all categories (TP_a_ + TP_b_ +TP_h_), which both result in the same score values. Tier boundaries were defined using P_33_ and P_67_ derived from the empirical distribution of Overall-Process Total Scores across all 140 studies.

#### 2.2.7. Sensitivity Analysis and Robustness of the Scoring Framework

All sensitivity analyses were performed in R using the readxl, dplyr, tidyr, and purrr packages. The analyses included category-weight sensitivity, alternative penalty spacing, Monte Carlo perturbation, and bootstrap evaluation of Tier-boundary stability. For each scenario, all relevant scores were recalculated, and Tier boundaries were re-estimated from the empirical 33rd (P_33_) and 67th (P_67_) percentiles of the modified score distributions. The unperturbed percentile-based classification was used as the baseline reference. Robustness was assessed using the percentage of process configurations retaining their baseline Tier and the Spearman rank correlation between the perturbed and baseline continuous scores. Refer to the Supplementary Material for more details.

#### 2.2.8. Illustrative Application of the Rubric

The illustrative application of the proposed CNC isolation Rubric system was conducted using representative studies to classify each of the three processing stages and the entire isolation process from Labor, Waste, and Cost perspectives. First, the chemicals and operational variables of the target CNC isolation process were listed and sorted by stage. Using Table 2, Section 1, the corresponding operational regimes and concentration-derived price categories were identified. The corresponding penalty points for Labor, Waste, and Cost were then assigned using the Scoring Framework in Table 2, Section 2.

Category-level Total Scores were calculated as L_total_ = L_a_ + L_b_ + L_h_, W_total_ = W_a_ + W_b_ + W_h_, and C_total_ = C_a_ + C_b_ + C_h_. Stage-level Total Scores were calculated as TP_a_ = L_a_ + W_a_ + C_a_, TP_b_ = L_b_ + W_b_ + C_b_, and TP_h_ = L_h_ + W_h_ + C_h_ in each stage. The Overall-Process Total Score (TP_total_) was obtained by summing either the Category-level or Stage-level Total Scores. Finally, the Category-level, Stage-level, and Overall-Process Total Scores were mapped onto the percentile ranges specified in the Tiering Framework in Table 2, Section 3, to obtain the corresponding Tier classifications. The complete step-by-step procedure and calculation examples are provided in the Supplementary Materials.

## 3. Results and Discussion

### 3.1. Overview of the Dataset and Summary of the Reviewed Literature

CNC isolation routes reported in the literature used a variety of alkaline and bleaching agents, as well as acids. These chemicals have been used in various combinations and under diverse experimental conditions (operational variables) across different studies to isolate CNC from lignocellulosic feedstocks. As described in the methods, inclusion of studies in the dataset required detailed procedural information on the operational variables of the CNC isolation process, covering all three main stages. Following consolidation of the Google Scholar-identified studies and the supplementary Publish or Perish/Crossref results, 886 candidate records were documented in the screening file (Figure S1).

Of these records, 75 were excluded as being related to computer numerical control (CNC) rather than cellulose nanocrystals. A further 112 studies were excluded because they were unrelated to CNC extraction or isolation. Of the remaining studies, 340 records were outside the defined analytical scope (focusing on other forms of cellulose (nano-fibrillated/micro-fibrillated cellulose) or employing other processing routes rather than the defined acid-hydrolysis-based pathway (such as oxidation-based methods)) and 18 were review articles. In addition, 217 potentially relevant papers were either unavailable online or lacked sufficient quantitative data or detailed operational variables. Finally, two duplicate entries corresponding to the same eligible studies were removed.

The final analytical dataset therefore comprised 140 unique studies listed in Supplementary Table S1. These studies contributed 686 experimental trials because individual publications frequently evaluated multiple feedstocks, chemical systems, treatment conditions, or combinations of optimizations.

**Figure 1:**
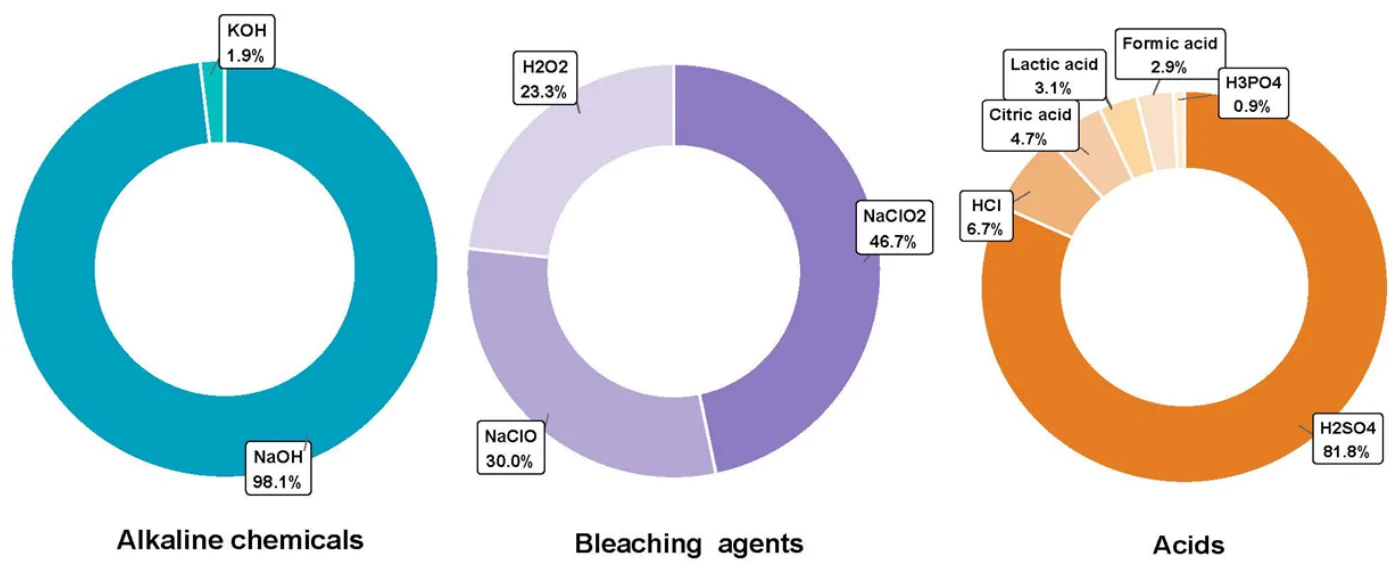
Distribution of alkaline chemicals, bleaching chemicals, and acid types used for the CNC isolation process across the reviewed literature.

Figure 1 summarizes the distribution of alkaline reagents, bleaching agents, and hydrolysis acids used for the CNC isolation across the reviewed literature. These distributions describe the defined analytical dataset and should not be interpreted as exhaustive estimates of publication frequency for the entire CNC-isolation literature.

Sodium hydroxide (NaOH) was the leading alkaline chemical utilized (98.1%), while potassium hydroxide (KOH) was applied in less than 2% of cases. The dominance of NaOH likely stems from its well-established, long history of application in the pulp and paper industry and lignocellulosic processing [41,42]; low cost and availability [42,43,44]; and proven effectiveness in swelling lignocellulosic biomass, removing hemicellulose, and achieving partial delignification [15,24,29]. However, its high frequency of use should not be interpreted as evidence of intrinsic or overall technical superiority over KOH, as such superiority has not been conclusively demonstrated in the literature. Regarding bleaching chemicals, sodium chlorite (NaClO_2_) was the primary bleaching agent employed in the studies (46.7%), with sodium hypochlorite (NaClO) and hydrogen peroxide (H_2_O_2_) accounting for 30% and 23.3%, respectively. Concerning acids for hydrolysis, sulfuric acid (H_2_SO_4_) was by far the most commonly used acid (81.8%). The widespread use of sulfuric acid is attributed to its ability to produce negatively charged cellulose nanocrystals by esterifying the surface hydroxyl groups of cellulose with sulfate groups. The resulting sulfated CNCs exhibit good colloidal stability and improved aqueous dispersibility [15,25,26]. However, sulfuric acid hydrolysis generates acidic, sulfate-containing waste [45], which requires repeated washing, centrifugation, or dialysis to neutralize the acid [46,47,48]. It can also reduce CNC thermal stability by promoting degradation via surface sulfate groups [49,50]. Hydrochloric- and organic-acid routes produce CNCs with different surface chemistries and may provide improved thermal stability [45,51,52]; however, their performance depends on route-specific combinations of acid concentration, temperature, reaction time, acid-to-cellulose ratio, neutralization, and mechanical post-treatment [45,48,52]. Therefore, the prevalent use of sulfuric acid primarily reflects its well-established, reproducible processing conditions rather than its overall superiority in terms of sustainability.

Before data analysis, the chemical concentration units were standardized (molarity for alkaline and bleaching chemicals and normality for acids) using the equations provided in Table 1. The estimated density values corresponding to the reported concentrations with decimal precision are presented in Table S2 and were used to calculate the chemicals’ concentrations. The obtained unified concentrations, along with other operational variables, were then subjected to statistical analyses to identify literature-derived distribution ranges designated as “operational regimes” for each variable.

### 3.2. Data Analyses and Distribution Ranges (Operational Regimes) of the Experimental Variables Involved in the CNC Isolation Process, Categorized by Chemical Type

**Figure 2:**
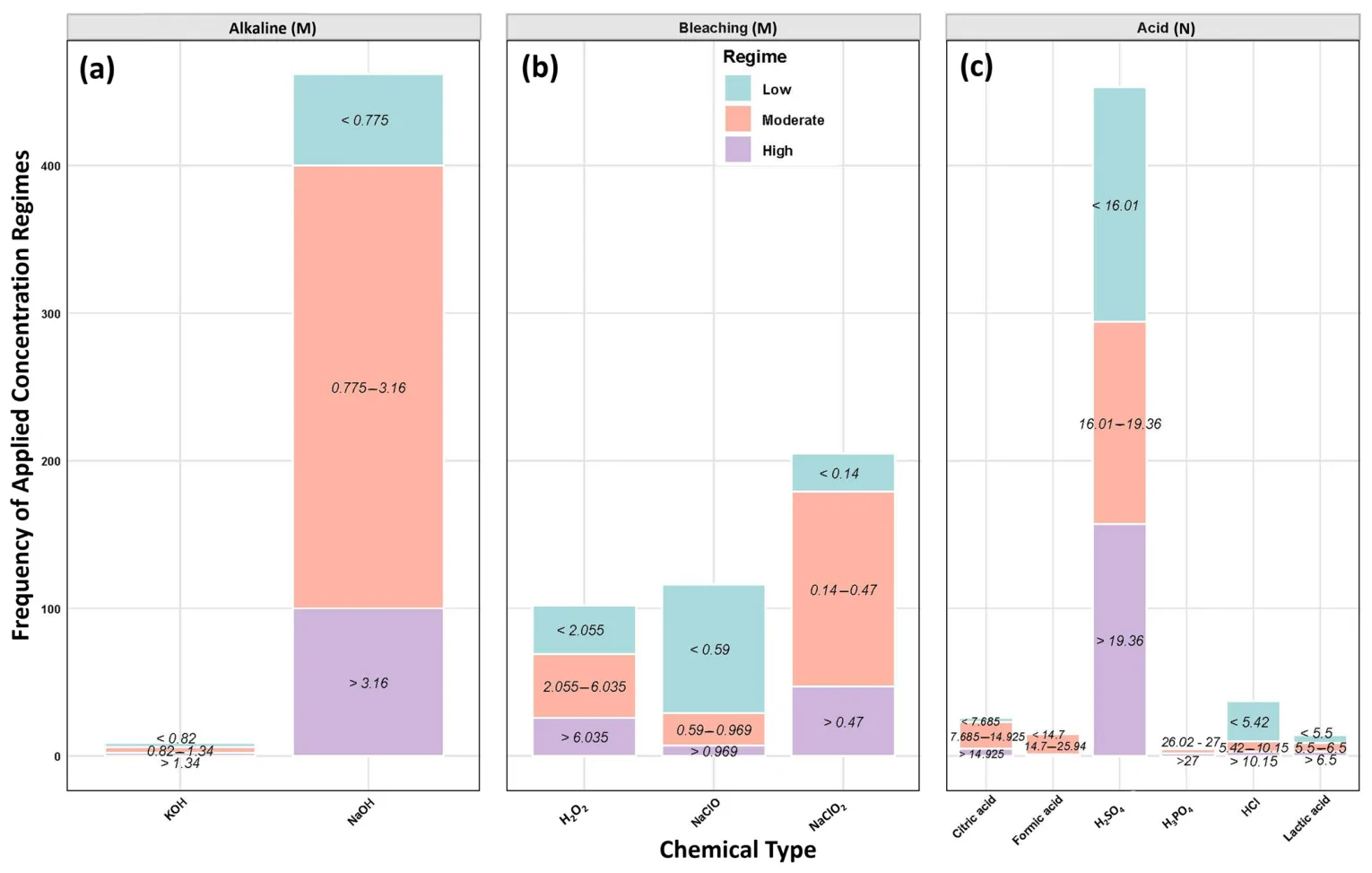
Literature-derived operational regimes (distribution ranges) of chemical concentration across the three stages of the CNC isolation process for the various chemicals used: (**a**) alkaline chemicals in molarity, (**b**) bleaching chemicals in molarity, (**c**) acids in normality. The values shown within the bars indicate the literature-derived concentration ranges defining the Low, Moderate, and High operational regimes for each chemical. Bar segment heights represent the frequency of each regime, expressed as the number of occurrence records across the analyzed dataset. Refer to Table 2 for the exact operational regime boundaries.

**Figure 3:**
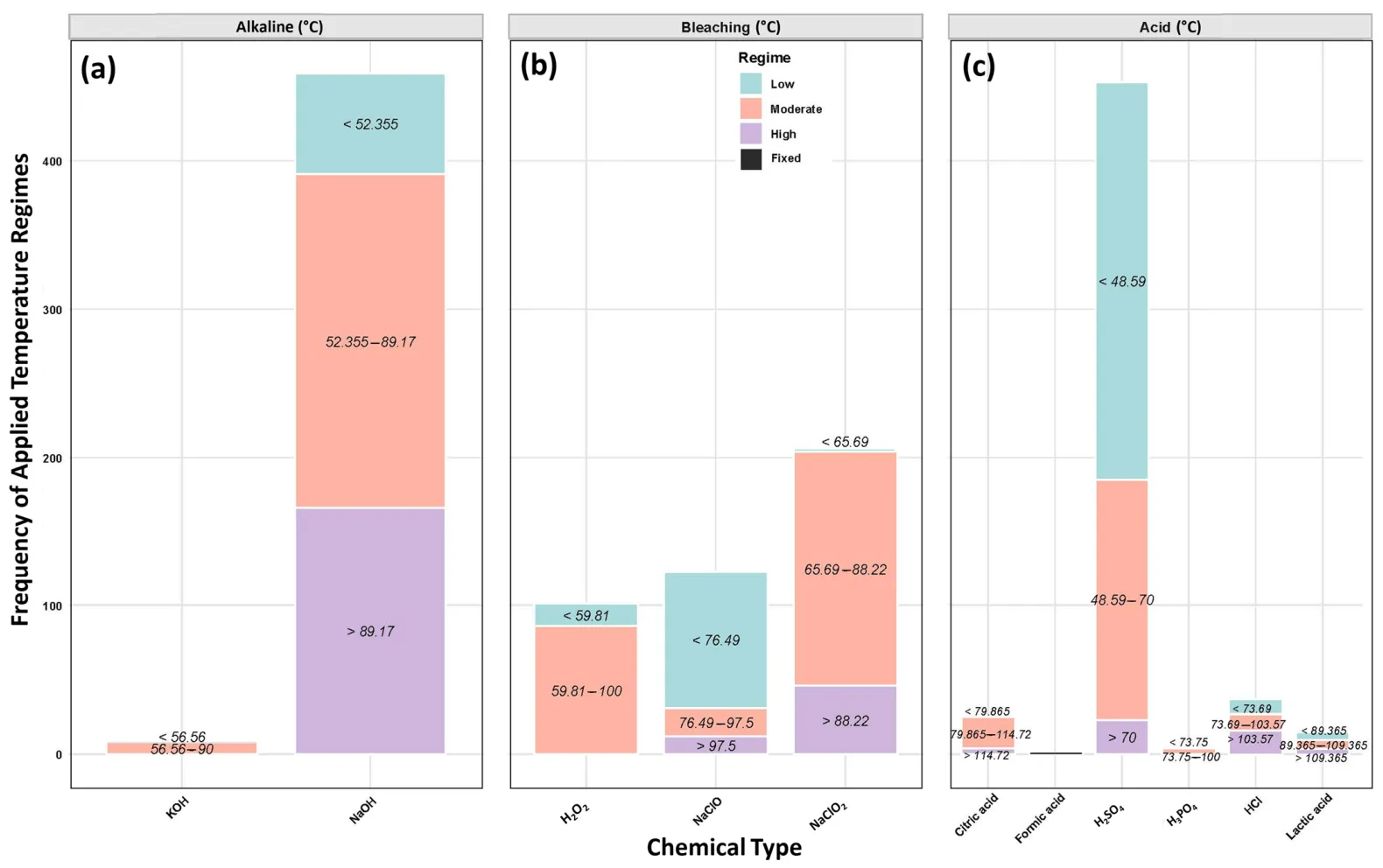
Literature-derived operational regimes (distribution ranges) of experimental temperature (°C) across the three stages of the CNC isolation process for the various chemicals used: (**a**) alkaline chemicals, (**b**) bleaching chemicals, (**c**) acids. The values shown within the bars indicate the literature-derived temperature ranges defining the Low, Moderate, and High operational regimes for each chemical. Bar segment heights represent the frequency of each regime, expressed as the number of occurrence records across the analyzed dataset. Refer to Table 2 for the exact operational regime boundaries.

**Figure 4:**
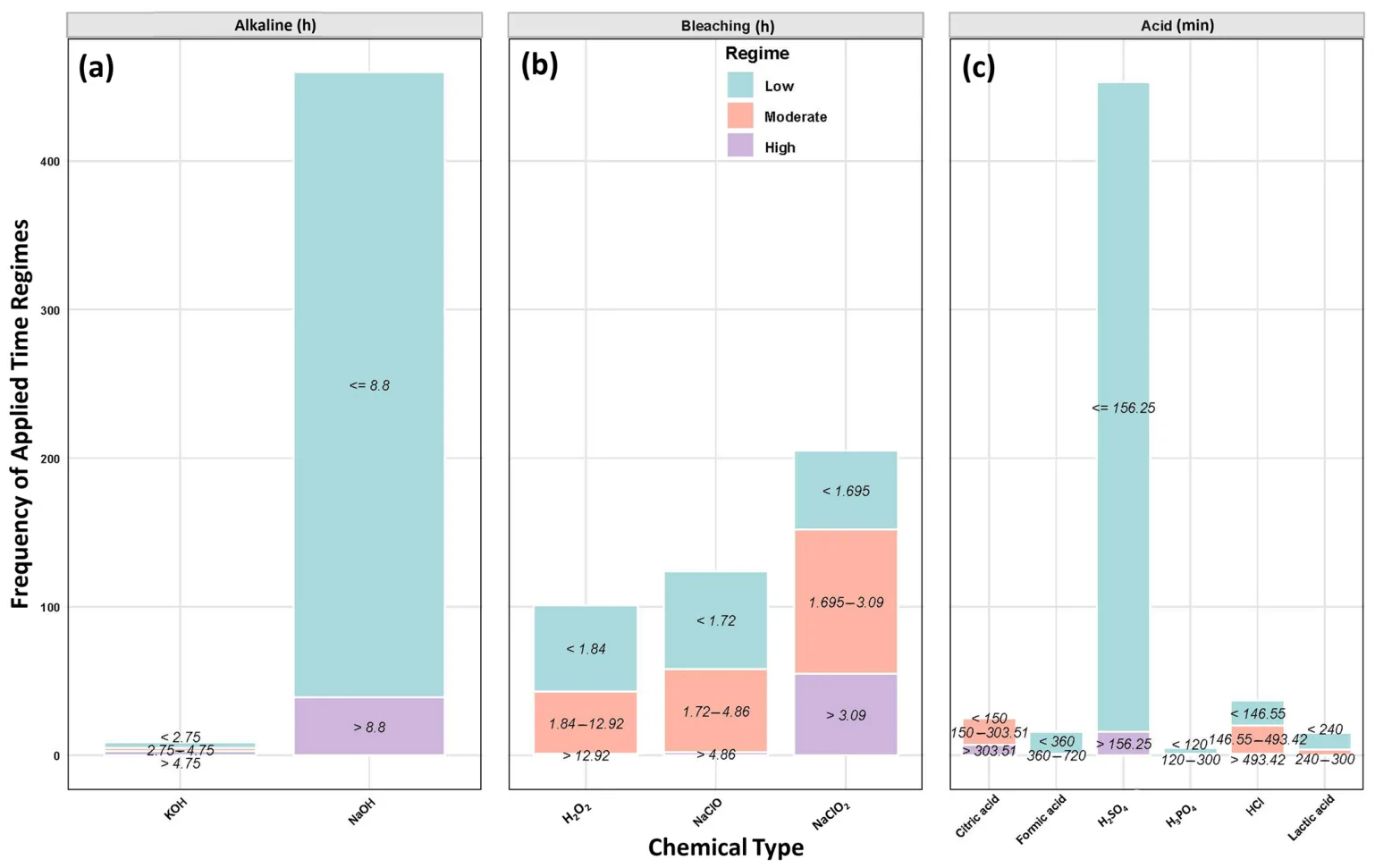
Literature-derived operational regimes (distribution ranges) of experimental time across the three stages of the CNC isolation process for the various chemicals used: (**a**) alkaline chemicals in hours, (**b**) bleaching chemicals in hours, (**c**) acids in minutes. The values shown within the bars indicate the literature-derived processing-time ranges defining the Low, Moderate, and High operational regimes for each chemical. Bar segment heights represent the frequency of each regime, expressed as the number of occurrence records across the analyzed dataset. Refer to Table 2 for the exact operational regime boundaries.

Normality tests (the Shapiro–Wilk test) revealed normal distributions (Shapiro–Wilk *p* > 0.05; |skewness| < 1) for various parameters of alkaline treatment and acid hydrolysis, whereas some variables involved in the bleaching stage, such as temperature, showed some deviations from the normal distribution. Additionally, across the three processing stages, none of the examined variable pairs exceeded the prespecified threshold for very high pairwise correlation (|r|≥ 0.80). Accordingly, no very high pairwise correlations were detected among concentration, temperature, time, and number of cycles. Outputs of the Gaussian Mixture Models (GMMs) used to identify operational regimes for various experimental variables (Figures S2–S6) demonstrated broad diversity in chemical concentrations and experimental variables of the CNC isolation processes across the literature. For example, the molarity values of NaOH used in the alkaline treatment stage (N = 475) revealed a strongly multimodal distribution. GMM identified four distinct components (BIC = −1214.31), with component means at approximately 0.53, 1.02, 1.84, and 4.48 M. The corresponding mixing proportions were 0.13, 0.22, 0.44, and 0.21, indicating that moderate NaOH concentrations (≈1.5–2.5 M) were most frequently reported, whereas very low and high concentrations were less common. These modes aligned closely with distinct concentration levels frequently applied across studies, signifying the presence of well-defined operational regimes rather than a continuous gradient of conditions. Based on the identified mixture-derived thresholds, NaOH molarity was subsequently classified into Low, Moderate, and High regimes for downstream scoring of Labor, Cost, and Waste impacts. The same analyses were conducted for all chemicals and experimental variables (categorized by chemical type), and the resulting distribution ranges (operational regimes) are depicted in Figure 2, Figure 3 and Figure 4 and listed in Table 2 (Section 1). The identified operational regimes were then used for developing the Scoring Rubric as presented in “Section 3.5”.

Figure 2 illustrates the literature-derived distribution of concentration ranges for each chemical, along with the frequency of each concentration range/regime (occurrence records across the dataset). The values shown within the bars indicate the literature-derived concentration ranges defining the Low, Moderate, and High operational regimes for each chemical. Bar segment heights represent the frequency of each regime, expressed as the number of occurrence records across the analyzed dataset. Refer to Table 2 for the exact operational regime boundaries. In the alkaline stage, the dominant alkaline chemical (NaOH) was most frequently used at moderate concentrations across the dataset. Similarly, the most widely used bleaching agent (NaClO_2_) was employed mainly in the moderate-concentration regime, whereas NaClO was predominantly used at low concentrations. In contrast, sulfuric acid (H_2_SO_4_), exhibiting the highest frequency of application among all acids, was used almost equally across all concentration regimes, whereas the second most dominant acid, HCl, was generally utilized within the low-concentration regime. Temperature-range distributions for each chemical across all three stages are depicted in Figure 3. Formic acid was the only chemical for which the model identified no distribution range, as it was applied at 95 °C in 94% of the reported cases and at 100 °C in the remainder; hence, it was assigned a fixed temperature (95 °C). Figure 4 shows the time distribution (operational time) for each chemical in each processing stage. The main highlights were again NaOH and H_2_SO_4_, which were most frequently applied within their low identified time regimes.

### 3.3. Ranking Chemicals According to Price

Chemical prices were calculated based on the cost of preparing 1 L of solution corresponding to the literature-derived concentration regimes. Details of the steps used to obtain chemical prices and price categories are provided in the “Section 2.2.4. Ranking Chemicals According to Price”, while calculation examples are provided in the Supplementary Material. The results are listed in Tables S3–S6, with the price categories and corresponding penalty points shown in Table S6 used later to develop the CNC Scoring and Tiering Rubric from a cost perspective. Final price categories are also listed in Table 2 (Section 1). The presented calculations and price categories can be used by researchers and industrial end users to compute the cost of desired chemicals based on prices available in their region or from local suppliers, and to categorize the resulting prices. However, the resulting estimates reflect laboratory-scale catalog prices rather than bulk industrial costs.

### 3.4. Ranking Chemicals According to Environmental Friendliness

Chemicals were evaluated according to post-use environmental burden in the wastewater stream (Table S7) and toxicity/ecotoxicity (Tables S8–S13) and assigned numerical severity ranks/penalty scores for each evaluation criterion, with higher values indicating greater environmental harm. The results are summarized in the following sections, with the total severity scores of the chemicals (Table S14) ultimately used to rank the chemicals within each stage from an environmental-friendliness perspective regarding waste generation.

#### 3.4.1. Post-Use Environmental Burden in Wastewater Streams

##### 3.4.1.1. Alkaline Chemicals (NaOH and KOH)

Regarding alkaline chemicals, both the hydroxide agents are potent alkali compounds that increase the pH and alkalinity of the wastewater, which subsequently affect pH-sensitive biological processes involved in nitrogen cycles in wastewater and its related treatment reactors [53,54,55,56]. Moreover, hydroxide ions in metal-bearing wastewater can form less soluble or precipitate hydroxide forms of metals [57], which can help immobilize heavy metals but can also deposit on infrastructure across various industries, such as desalination membranes, wastewater treatment plants, and water circulation systems, which is known as the scaling problem. Wastewater is typically treated for reuse, with the reclaimed water sometimes used for irrigation. From the irrigation viewpoint, sodium is the principal cation responsible for lowering soil quality by affecting soil structure (decreasing cohesion among soil particles), which results in soil dispersion and eventually susceptibility to water erosion (soil sodicity) [58,59,60]. Therefore, residual sodium ions in reclaimed water will lead to soil dispersion, reduced water infiltration, and ultimately crop yield loss if the reclaimed water is applied for agricultural irrigation. Although excess potassium in effluent raises concerns about surface water quality [60], this nutrient is the second most abundant nutrient for plants after nitrogen, which promotes plant growth in the agricultural sector [61]. Therefore, overall, the environmental burden of residual sodium derived from sodium hydroxide in wastewater seems to be greater than that of potassium derived from potassium hydroxide, as reflected by a greater assigned penalty point in Table S7.

##### 3.4.1.2. Bleaching Chemicals (NaClO, NaClO_2_, and H_2_O_2_)

With regard to bleaching chemicals, sodium hypochlorite is widely used as a disinfectant in both household and industrial applications. However, disinfection byproducts (DBPs), such as haloacetic acid (HAAs), trihalomethanes (THM4), haloacetonitriles (HANs), and haloketones (HKs), have been reported to form due to the reaction of natural organic matter (such as microorganisms’ biofilm, activated sludge mixture) with chlorine from residual disinfectant [62,63]. Therefore, DBP formation has become a challenge in cleaning membrane surfaces in bioreactors and in the final stage of water disinfection in water treatment plants [62,63]. The genotoxicity, cytotoxicity, and carcinogenicity of these DBSs (HAAs and THM4) have been previously proven [62,64]. Interestingly, increasing solution pH, reaction time, temperature, and chlorine dose were shown to increase HAA creation [63]. Hence, these compounds have caught the attention of regulatory frameworks and are treated as regulated by-products due to their toxicity and carcinogenicity [65]. Chlorate (ClO_3_^−^) can be found as an impurity in sodium hypochlorite. The chlorate concentration in sodium hypochlorite increases with longer chemical storage time and higher storage temperature [66]. Given adequate time, sodium hypochlorite can be converted entirely to chloride, perchlorate (ClO_4_^−^), and chlorate [67], with the last two being extremely difficult to remove from water due to their stability. Moreover, chlorine (Cl_2_) is very reactive in the aquatic environment. Hence, it does not remain for an extended time and forms other by-products such as hypochlorous acid (HOCl) and hydrochloric acid (HCl) that display extended residence time, raising environmental concerns. Free chlorine refers to Cl_2_, HOCl, and the hypochlorite ion (OCl^−^) in equilibrium, with their relative amounts controlled by pH, temperature, and ionic strength. Cl_2_ is the main species at very low pH, while HOCl becomes the leading ion at pH 2–7. There is an equal molar proportion of the two species HOCl and OCl^−^ at pH 7.4 and 20 °C [68]. Sodium chlorite, the most widely used chemical for generating chlorine dioxide (ClO_2_) in drinking water treatment, can also contain chlorate impurities [66]. In contrast to sodium hypochlorite, it generates chlorine dioxide gas in situ, an effective and selective oxidizing agent that does not chlorinate natural organic matter (negligible THMs/HAAs). However, chlorine dioxide and chlorite, which originate from sodium chlorite, react with free chlorine to form the chlorate ion, which is highly recalcitrant, making wastewater purification challenging [69]. Both chlorate and chlorite are regulated DBPs owing to their adverse health effects [65,66]; therefore, they should be monitored because of their toxicity and persistence. Indeed, chlorate and chlorite are reversibly convertible, so they can coexist during drinking water treatment with hypochlorite solution and chlorine dioxide [66]. Consequently, wastewaters containing NaClO and NaClO_2_ display the same environmental burdens as those discussed above for the water-treatment systems, since residual oxidants and their reaction products (e.g., chlorate, chlorite, free chlorine, THMs, HAAs) persist, impair biological treatment, and contribute to regulated toxic by-products. Unlike sodium hypochlorite and sodium chlorite, non-chlorinated hydrogen peroxide is a green bleaching/oxidizing agent [70], which decomposes to water and oxygen, leaving no persistent chlorinated burden in the environment [71]. However, it is an oxidizer that requires safe handling and use, similar to other chemicals. Overall, the environmental burden of sodium hypochlorite is greater than that of sodium chlorite, with hydrogen peroxide being the safest bleaching agent (Table S7).

##### 3.4.1.3. Acids for Hydrolysis

As for the acids, the organic acids (formic, lactic, and citric acids) are weak acids whose dissociation in aqueous media is partial (existing mainly in undissociated forms) [72,73]. However, they are readily biodegradable [74,75,76,77,78]. Thus, when considering the post-use burden of organic acids in wastewater streams, they leave little to no persistent burden in the effluent. For this reason, they are typically utilized as external carbon sources for denitrification in wastewater treatment plants to mitigate nitrogen content [79,80,81,82]. Hence, their residual acidity can be assumed to be biologically consumed. In this regard, the smaller and simpler the acid, the more readily it will be consumed by denitrifying bacteria, resulting in more efficient denitrification [83]. Accordingly, monotropic organic acids like formic and lactic acid with only one carboxylic group are easier to consume as compared to citric acid with three carboxylic groups (triprotic). In addition, the most complex acid among the listed acids, citric acid, has been utilized in the literature as an organic chelator to increase the mobility of heavy metals such as cadmium, enhance plant uptake, and eventually improve phytoremediation [84,85,86]. This is an advantage for remediation frameworks, but it might interfere with metal removal from wastewater if acid residuals persist, which can constitute a post-use burden. On the whole, the order of post-use burden for organic acids can be considered as follows: Formic acid = Lactic acid < Citric acid (Table S7). In contrast, mineral acids dissociate into persistent inorganic ions, each with its own drawbacks. For instance, sulfate, originating from sulfuric acid, is responsible for odor in domestic wastewater due to conversion to hydrogen sulfide [87,88]. Subsequently, hydrogen sulfide can be biologically oxidized again to sulfuric acid, causing corrosion of concrete infrastructure in sewer pipes and wastewater systems [89,90,91,92]. This anion raises another concern: the scaling problem due to the formation of sulfate-containing salts [93,94,95]. Chloride, HCl-derived inorganic ion, has been reported to damage pipelines in reclamation water plants and cause concrete deterioration in facilities containing concrete, such as wastewater treatment plants and composite concrete structures [96,97], due to its corrosive nature. In addition, chloride is not targeted for elimination in wastewater treatment plants (no standard effluent limit exists), resulting in its entry into water-reclamation industries, which can subsequently damage crops if irrigated with reclaimed water [97]. Phosphate originating from spent phosphoric acid imposes its own problems in domestic wastewater, such as scaling associated with struvite (precipitation of phosphate together with ammonium and magnesium as MgNH_4_PO_4_:6H_2_O) in wastewater treatment facilities [98]. More importantly, phosphate in wastewater streams poses adverse effects on aquatic life due to eutrophication if the level is elevated in the effluent. Therefore, many developed countries have set strict regulations for the nutrient concentrations (in particular, phosphate) in the effluent to mitigate eutrophication [99,100,101,102]. Thus, the post-use burden of mineral acids can be concluded as: hydrochloric acid < sulfuric acid < phosphoric acid (Table S7).

It should be noted that these relative post-use burden rankings provide general comparative guidance and should not be regarded as universally applicable. The environmental significance of each burden may vary depending on local water sources, wastewater treatment technologies, infrastructure, effluent discharge or reuse practices, receiving environments, and regulatory requirements. For example, scaling in desalination systems is particularly relevant in regions that depend on desalinated water but less so in regions where drinking water is primarily supplied from rivers or groundwater. Future research should therefore evaluate these burdens within the relevant local context, and users should adapt and validate the proposed rankings before applying them to a specific region or production system.

#### 3.4.2. Toxicity and Ecotoxicity of the Chemicals

##### 3.4.2.1. Alkaline Chemicals (NaOH and KOH)

The toxicity and ecotoxicity values for alkaline chemicals are listed in Table S8. The aquatic toxicity of the alkaline chemicals showed opposite trends for fish and microcrustaceans (water flea; Daphnia), while sodium hydroxide exhibited higher oral toxicity to animals and was therefore ranked as overall more toxic.

##### 3.4.2.2. Bleaching Chemicals (NaClO, NaClO_2_, and H_2_O_2_)

Table S9 summarizes the toxicity/ecotoxicity values of the bleaching chemicals, with their corresponding severity ranks (penalty points). The total environmental severity rank revealed that hydrogen peroxide was slightly more toxic to the tested aquatic organisms than sodium chlorite, with sodium hypochlorite being substantially the most toxic. The chemical scores for different animal toxicity benchmarks indicated that sodium chlorite and sodium hypochlorite were the most and the least toxic agents, respectively, with the non-chlorinated bleach being intermediate (Table S9).

##### 3.4.2.3. Acids for Hydrolysis

The results of toxicity/ecotoxicity assessments for acids are listed in Table S10. Since the toxicity values were available for different fish species, the acids were initially ranked via intra-species toxicity values as follows (from the most toxic to the least, Table S11, with corresponding toxicity values (mg/L) presented in parentheses):

Rainbow trout fish: Phosphoric acid (87) > Citric acid (100) > Lactic acid (130).

Bluegill fish: Sulfuric acid (16–28) > Hydrochloric acid (20.5) > Phosphoric acid (60) >> Citric acid (1516).

Zebrafish: Formic acid (130) > Sulfuric acid (500).

Given the above rankings, formic and lactic acids showed similar toxicity values and ranked equally as the safest acids toward fish, while citric acid was considered the most toxic organic acid due to its smaller toxicity value (100 vs. 130). Hydrochloric acid was revealed to be more toxic than both formic and lactic acids, as its lowest toxicity figure (20.5) was much lower than that of formic and lactic acids (130), indicating greater toxicity to fish. Similarly, sulfuric (16) and phosphoric (60) acids were ranked as more toxic than formic acid (130). The overall fish toxicity ranking is presented below (from the most toxic to the least toxic, Tables S10 and S11), which is consistent with the intra-species toxicity ranking, indicating that mineral acids pose a higher aquatic hazard than organic acids:

Sulfuric acid > Hydrochloric acid > Phosphoric acid > Citric acid > Formic acid = Lactic acid.

Toxicity rankings of acids against Daphnia and Algae were similar, with hydrochloric acid showing the highest toxicity to both. This was followed by triprotic organic acid (citric acid), which was more toxic than the mineral acids sulfuric acid and phosphoric acid, and the monoprotic organic acids (formic and lactic) were the safest.

According to total aquatic toxicity, the acids were overall ranked as follows (from the most toxic to the least toxic, Table S10):

Total Environmental (aquatic) toxicity severity (Fish + Daphnia + Algae): Hydrochloric acid > Sulfuric acid > Citric acid > Phosphoric acid > Formic acid = Lactic acid.

Regarding animal toxicity thresholds, the lack of reported dermal toxicity data for formic and sulfuric acids excluded this benchmark from consideration. Acids exhibited varying degrees of toxicity, depending entirely on the toxicity benchmarks. However, the overall ranking showed that the three mineral acids had the same degree of hazard and were more toxic than the organic acids (Table S10).

It is worth noting that these rankings reflect the relative inherent toxicity and ecotoxicity of the chemicals, based on reported toxicity benchmarks, and should not be interpreted as site-specific environmental risk estimates. Actual exposure and risk depend on local wastewater-treatment practices, neutralization, biodegradation, dilution, discharge or reuse pathways, receiving environments, and the organisms exposed. For example, fish toxicity is especially relevant when untreated or unsatisfactorily treated wastewater is discharged into rivers, whereas readily biodegradable compounds such as citric acid may be substantially removed before discharge with effective wastewater treatment. Hence, future applications of these rankings should consider local conditions and adapt or validate the assigned scores accordingly.

#### 3.4.3. Toxicity and Ecotoxicity of the Inorganic Ions

##### 3.4.3.1. Alkaline Chemicals (NaOH and KOH)

There are no health-based guideline thresholds for sodium (representative of sodium hydroxide) or potassium (representative of potassium hydroxide) in drinking water [103,104]. However, sodium values exceeding 200 mg/L in drinking water result in an unpleasant taste [103], as shown in Table S12. According to acute toxicity benchmark values, potassium was 3–6 times more toxic than sodium and was therefore assigned a higher penalty point (Table S12).

##### 3.4.3.2. Bleaching Chemicals (NaClO, NaClO_2_, and H_2_O_2_)

Based on the acute and chronic toxicity benchmark figures for the ions associated with each beaching chemical (Table S12), both agents received similar total penalty points and were thus ranked equally. However, according to the drinking water guideline values, sodium chlorite had a stricter threshold (lower) and thus appeared more toxic.

##### 3.4.3.3. Mineral Acids for Hydrolysis (H_2_SO_4_, HCl, and H_3_PO_4_)

The toxicological values reported for ions originating from mineral acids are summarized in Table S13. The ranking results revealed different outcomes for the three mineral acids, with phosphoric and hydrochloric acids ranking worst for the acute benchmark and drinking water quality, respectively.

#### 3.4.4. Overall Ranking of Chemicals from an Environmental Friendliness Perspective

The total penalty points, derived from Tables S7–S13, are listed in Table S14 and were used for the overall ranking of the chemicals in each group based on environmental friendliness. The final ranking indicated that sodium hydroxide was more harmful to the environment than potassium hydroxide and would require a greater penalty in the CNC Scoring and Tiering Rubric for the waste category (the next section). Among the bleaching chemicals, hydrogen peroxide was by far the greenest bleach and would be assigned the lowest penalty points, while sodium chlorite would deserve the most penalty points. Among the acids, hydrochloric acid was the most environmentally harmful, followed by sulfuric and phosphoric acids. The organic acids ranked as the safest for the environment, with lactic acid and citric acid determined as the most and least environmentally friendly, respectively. The waste-related penalty points would decrease by moving from hydrochloric acid to lactic acid, as defined in the next section.

### 3.5. CNC Scoring and Tiering Rubric: A Practical Guide

#### 3.5.1. Scoring Details

Outputs of environmental friendliness order and price categories of the chemicals involved in each processing stage of CNC isolation, along with the literature-derived distribution ranges for operational variables of the stage treatments, were used to develop a practical data-driven Scoring and Tiering Rubric system that classifies CNC isolation stages and the overall process into three Tiers based on relative operational burden across the literature from three core categories: Labor intensity, Waste generation, and Cost:

Tier 1: Lowest combined operational burden relative to other protocols.

Tier 2: Intermediate burden.

Tier 3: Highest combined operational burden.

This classification enables the identification of protocols with comparatively lower combined operational burden (more cost-effective, environmentally favorable, and less labor-intensive) at the overall CNC isolation process, stage, and category levels. Detailed information on the scoring procedure for each stage and category is provided in the Supplementary Material, “Section 2.2.6”, and summarized in Table 2 (Section 2: Scoring Framework). Accordingly, each stage was assigned penalty points across the three categories, and the total penalty scores for each stage within each category, together with the total penalty points for each category across the three stages, were used for Tier classifications outlined in the next section.

#### 3.5.2. Tier Calculation: Stage-Level Total Tier Mapping

Tier classification, the final output of the CNC Scoring and Tiering Rubric, was presented as Stage-Level Total Tier, Category-Level Total Tier, and Overall-Process Total Tier. Percentile-based thresholds (P_33_ and P_67_), derived from the empirical distribution of total scores across all 140 studies, were used to define the Tier boundaries. Percentile-based thresholds offer a data-driven classification that is less sensitive to outliers and provides a fair comparison across diverse experimental designs. Details of the procedure used to compute Total Scores and Tiers are presented in the “Section 2.2.6.5. Tier Calculation: Stage-Level Total Tier Mapping” and summarized in Table 2 (Section 3: Tiering Framework).

Stage-Level Total Scores were calculated for each study by summing the three category scores (Labor, L; Waste, W; Cost, C) within each stage (e.g., total points of alkaline treatment (TP_a_) = L_a_ + W_a_ + C_a_). P_33_ and P_67_ of the Stage Totals across the dataset were used to define the Tier boundaries. Stage-Level Total Scores indicate which treatment stage contributes most to the overall CNC process. Stage-Level Tiers reflect integrated operational burden and should be interpreted as a compound measure rather than a guarantee of optimal performance in every category. Stage-Level Tier 1 indicates that the combined operational burden (Labor + Waste + Cost) is among the lowest third of the reviewed literature, which does not necessarily imply that each individual category score is minimal; instead, it reflects a comparatively favorable overall operational profile. This also applies to Tiers 2 and 3.

The Tier classifications were derived from empirical percentile distributions within the defined 140-study dataset and therefore represent relative comparisons within the reviewed literature rather than fixed or universal benchmarks of optimization or sustainability. The Tier boundaries may shift as the dataset composition changes, particularly for process configurations with scores close to the P_33_ or P_67_ thresholds.

**Figure 5:**
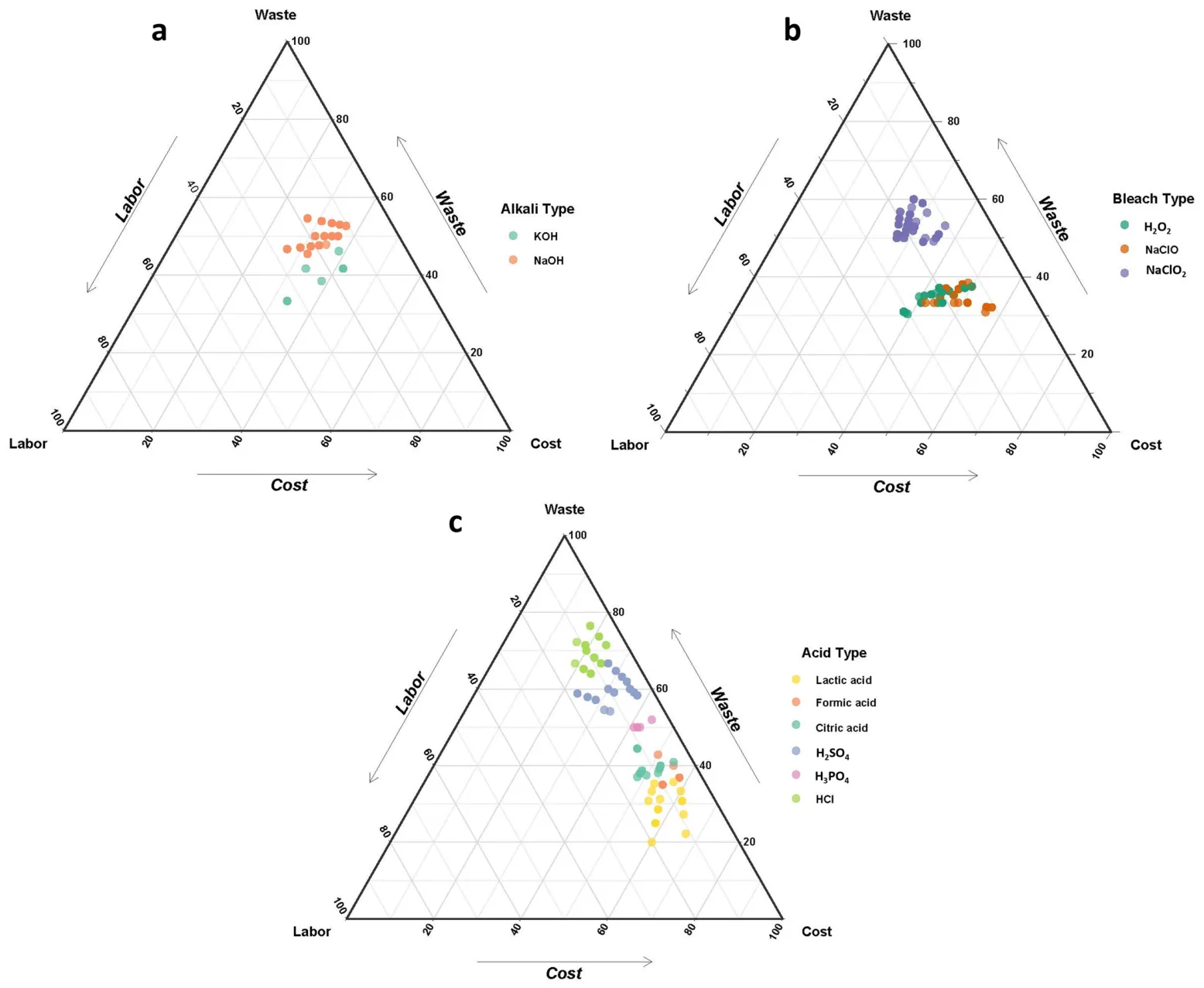
Literature-derived ternary composition of operational burden (Labor, Waste, Cost) for the three CNC isolation stages: (**a**) alkaline treatment, (**b**) bleaching treatment, (**c**) acid hydrolysis. Each point represents a reported protocol, colored by reagent type. Because multiple protocols share identical Labor–Waste–Cost proportions, several points overlap and appear at the same position in the ternary space, resulting in fewer visually distinguishable points than the total number of analyzed protocols. The complete individual-level scores and ternary coordinates underlying the plots are provided in the Supplementary Excel File.

As shown in Table 2 (Section 3), Tier spans vary across processing stages, indicating heterogeneous methodological approaches throughout the literature. Alkaline treatment showed relatively narrow thresholds (P_33_ = 7.5 and P_67_ = 8.5), signifying more standardized and relatively consistent operational protocols across studies. Consistently, alkaline protocols cluster tightly in the ternary composition space, dominated by minimal separation between alkali types, revealing similar burden shares between NaOH and KOH (Figure 5a). On the contrary, the bleaching treatment revealed the widest percentile range (P_33_ = 8.75 and P_67_ = 11.75), suggesting substantial variability in operational variables across the dataset. This is consistent with the ternary plot (Figure 5b), which shows distinct, chemical-specific clusters reflecting systematic differences in burden allocation, with NaClO_2_ inhabiting a separate region characterized by relatively higher waste contributions, and NaClO and H_2_O_2_ protocols displaying more similar burden distributions. Acid hydrolysis demonstrated intermediate variability (P_33_ = 8.5 and P_67_ = 10). Figure 5c illustrates a broad distribution along the Waste–Cost edge, indicating considerable differences in burden allocation among acids, while Labor contributions remain consistently low. Although acid hydrolysis protocols span a wide range of Waste–Cost compositions in the ternary plots, their Stage-Level Total Scores were more tightly distributed, indicating compositional differences, where variations arise mainly from the allocation of operational burden (the relative contributions of Waste and Cost) and not from the differences in overall operational burden. Among all stages, compositions were skewed toward the Waste–Cost axes and away from the Labor vertex, suggesting that Labor contributes comparatively less to the operational burden than Waste generation and Cost in most reported protocols. These patterns confirm that the operational burden in the CNC isolation is indeed stage-specific, with certain steps having a greater impact on overall process sustainability. In addition, stages with higher Tier thresholds, such as bleaching, generally impose greater combined operational burden than alkaline treatment, demonstrating a higher overall process intensity.

#### 3.5.3. Tier Calculation: Category-Level Total Tier Mapping

Category-Level Total Scores were calculated for each study by summing the scores of the three stages within each category (e.g., total labor points (L_Total_) = L_a_ + L_b_ + L_h_, where a, b, and h refer to alkaline treatment, bleaching treatment, and acid hydrolysis, respectively). Refer to the “Section 2.2.6.6. Tier Calculation: Category-Level Total Tier Mapping” for more details. P_33_ and P_67_ of Category Totals across the dataset were used to define the Tier boundaries. The Category-Level Total Scores (L_Total_, W_Total_, and C_Total_) identify which category dominates or contributes most to the overall CNC process. Category-Level Tiers reflect integrated burden across processing stages and should be interpreted as a combined measure rather than a guarantee of minimal burden at each stage. Category-Level Tier 1 indicates that the total burden for that category is among the lowest third of the reviewed literature, which does not necessarily imply that each stage-specific score is minimal; instead, it reflects a comparatively favorable overall operational profile for the given category. This also applies to Tiers 2 and 3.

According to the resulting thresholds (Table 2, Section 3: Tiering Framework), Waste, with P_33_ and P_67_ of 14.25 and 16, respectively, contributed most to the overall burden (largest share) in comparison with Labor (P_33_ = 3.25 and P_67_ = 4) and Cost (P_33_ = 9 and P_67_ = 10.25), as reflected by its noticeably higher W_Total_ values. This is consistent with Stage-Level compositions (Figure 5), where protocols were concentrated toward the waste-dominated region of the ternary diagrams across all stages. The findings demonstrated that waste management and effluent generation are key drivers of process sustainability in CNC isolation. Labor showed comparatively lower totals and a narrower percentile range, reflecting smaller, more consistent contributions across studies, whereas Cost fell between Labor and Waste in both magnitude and variability. In addition, the broader percentile range observed for W_Total_ underscores substantial heterogeneity in waste-related requirements (waste management practices) across studies, underscoring the importance of waste management strategies in determining overall process performance.

#### 3.5.4. Tier Calculation: Overall-Process Tier Mapping (Entire CNC Isolation Process)

Overall-Process Total Scores can be obtained either by summing Total Category Scores across all stages, or by summing Total Stage Scores across all categories, as explained in the Section 2.2 (Section 2.2.6.7. Tier Calculation: Overall-Process Tier Mapping (Entire CNC Isolation Process)). P_33_ and P_67_ of the Overall-Process Totals across the dataset were obtained as 26.25 and 29.75, respectively, which were used to define the Tier boundaries (Table 2, Section 3: Tiering Framework).

The Overall Total Tiers (TP_Overall_) provides a single metric that reflects the combined contributions from all stages and categories, representing the overall burden of the entire CNC isolation workflow. The two complementary metrics (stage-specific and category-specific totals) enable distinguishing between protocols dominated by a single burden dimension and those exhibiting balanced performance across both categories and stages. The illustrative application of the proposed Scoring and Tiering Rubric is discussed later through examples from the reviewed literature.

### 3.6. Sensitivity and Robustness of the Scoring Framework

The sensitivity analyses demonstrated that the framework was generally robust to plausible changes in category weights and penalty assignments (Table 3 and Table 4). With regard to the category-weight sensitivity test, excluding the unchanged baseline scenario, overall-process Tier stability ranged from 91.5% to 100%, while Spearman rank correlations ranged from 0.995 to 0.999, as shown in Table 3. The greatest change occurred when the Waste contribution was reduced by 25% (91.5% retention), whereas a 25% reduction in the Labor contribution left the classification entirely unchanged; in every scenario, more than 90% of configurations retained their baseline Tier. These findings indicate that the classification was highly robust to reasonable variation in category weighting.

In the alternative penalty spacing test, all compressed schemes reproduced the baseline Tier classification exactly, with 100% stability and Spearman correlations (ρ) = 1.00 (Table 3). Under the expanded schemes, overall-process Tier stability remained high, ranging from 86.7% to 93.6%, with Spearman correlations of 0.960–0.977 (Table 3). Expansion of concentration penalties produced the largest changes in concentration-dominated metrics, particularly the alkali-stage total and the Cost-category total, which retained 69.2% and 75.5% of their baseline classifications, respectively. Nevertheless, the overall-process ranking and Tier classification remained largely preserved, indicating that the framework was not strongly dependent on the original numerical spacing of the penalties.

Regarding the Monte Carlo perturbation, under category-weight perturbation, mean Tier retention exceeded 97% for all stage-level metrics, and the overall-process score reached 100% for the category totals and averaged 97.7% for the overall Tier (Table 3). The mean Spearman correlation with the baseline ranking was 0.997 (Table 3). Under independent component-penalty perturbation, mean overall Tier retention remained 83.5% (Table 3), and 41.8% of configurations retained their baseline Tier in at least 90% of simulations. Thus, the framework was highly stable under category-level uncertainty and remained reasonably robust under the more stringent independent perturbation of all component penalties.

Results of the Bootstrap stability of Tier boundaries are listed in Table 4. The Tier boundaries were generally stable. For the alkali-stage total, acid-hydrolysis total, total Labor score, and overall-process score, the bootstrap median thresholds equaled the published thresholds and had narrow 95% intervals. The bleaching-stage, total Waste, and total Cost thresholds showed slightly greater variation, but their bootstrap medians remained equal to or close to the published values. For total Cost, the bootstrap median P_33_ was 8.75, compared with the published value of 9.00, while the 95% bootstrap interval of 8.75–9.25 included the published threshold. This indicates that the small discrepancy was consistent with expected sampling variability.

Together, the four sensitivity tests demonstrated that both the continuous ranking of process configurations and the percentile-based Tier classifications were robust to reasonable variation in category weights, ordinal penalty spacing, individual component penalties, and dataset composition. Most Tier changes occurred among configurations positioned near P_33_ or P_67_, which is expected because observations close to a classification boundary are inherently more likely to change Tier following small score perturbations.

### 3.7. Illustrative Applications of the Rubric

Three representative case studies were selected to illustrate how the proposed scoring framework differentiates CNC isolation protocols across operational burden levels, Tier 1 to Tier 3. These examples demonstrate how variations in chemical concentration, reaction time, and cycles across the alkaline and bleaching treatment and acid hydrolysis stages affect the overall operational burden score. The experimental variables in the example studies, along with the corresponding penalty scores for Labor, Waste, and Cost and the Tier classifications, are presented in Table 5. The Tier 1 protocol (Case Study 1) represents the protocol with the lowest assigned operational-burden score among the illustrative examples (TP_Overall_ = 20.5, Table 5). The alkaline treatment was classified as Tier 1, employing a low operational regime for reaction time and cycle (single cycle) at moderate NaOH concentration and temperature, achieving total penalty points of 1 for Labor (+0.5 for cycle and +0.5 for time, Table 2, Section 2), 4 for Waste (+2.5 out of +3 for concentration as NaOH is less environmentally preferred than KOH, +1 for temperature (representing a medium penalty), and +0.5 for cycle, Table 2, Section 2), and 2.5 for Cost (+1 for concentration, +1 for temperature, +0.5 for cycle, Table 2, Section 2), leading to total alkaline penalty points (TP_a_) of 7.5 and classification as Tier 1 (Table 2, Section 3 and Table 2). Bleaching was conducted using the greenest bleaching chemical (H_2_O_2_) at a low concentration under low regimes for time and cycle and a moderate operational temperature, achieving total penalty points of 1 for Labor (+0.5 for cycle and +0.5 for time, Table 2, Section 2), 2 for Waste (+0.5 out of +4.5 for concentration (reflecting the lowest waste penalty due to the use of an environmentally friendly chemical), +1 for temperature, and +0.5 for cycle, Table 2, Section 2), and 2.5 for Cost (+1 out of +6.5 for concentration, +1 for temperature, +0.5 for cycle, Table 2, Section 2), leading to total bleaching penalty points (TP_b_) of 5.5 and finally Tier 1 classification (Table 2, Section 3 and Table 5). Acid hydrolysis was performed using sulfuric acid, the most commonly used acid, at a low concentration under low-reaction-time and -temperature regimes. Therefore, this stage resulted in total penalty points of 0.5 for Labor (+0.5 for time, Table 2, Section 2), 5 for Waste (+4.5 out of +7 for concentration, +0.5 for temperature, Table 2, Section 2), and 2 for Cost (+1.5 for concentration, +0.5 for temperature, Table 2, Section 2), leading to total hydrolysis penalty points (TP_h_) of 7.5 and classification as Tier 1 (Table 2, Section 3 and Table 2). Overall, employing a bleaching cycle with minimal waste generation through inclusion of a green bleaching chemical with limited operational burden, combined with alkaline and acid hydrolysis stages conducted under low-to-moderate operational regimes, placed this CNC isolation protocol in Tier 1 (lowest integrated labor, waste, and cost scores), suggesting a comparatively lower-burden route within the present scoring framework.

The Tier 2 protocol (Case Study 2) exhibits an intermediate operational burden (TP_Overall_ = 28.5, Table 5). The alkaline treatment, with total penalty points (TP_a_) of 8.5, fell into Tier 2 owing to higher Waste and Cost penalty points (4.5 and 3, respectively, Table 5) compared to Case Study 1, while both studies had the same Labor penalty points. This resulted from the use of NaOH at a fivefold higher concentration, which increased both waste generation and cost, ultimately raising the Stage-level Total Tier by one level. The bleaching treatment in this study was conducted using a moderately environmentally friendly bleaching agent (NaClO) under low operational regimes for all parameters (similar to Case Study 1), which allowed the stage to remain at Stage-level Tier 1 (TP_b_: 8, Table 5). However, acid hydrolysis here was done using HCl, identified as the most environmentally harmful acid in this analysis, under a low-concentration regime but at a high temperature using a pressurized vessel (high pressure). Therefore, although the acid concentration and hydrolysis reaction time were both at their lower regimes, the use of a more environmentally harmful acid and operation under high-temperature and -pressure conditions resulted in substantially greater Labor demand, Waste generation, and Cost (Table 5), ultimately placing this stage in Tier 3 (TP_h_: 12, Table 2). Overall, although the bleaching stage remained relatively low in operational burden, the alkaline treatment and hydrolysis stages, which involved highly concentrated NaOH and the most environmentally hazardous acid, respectively, substantially increased chemical consumption and downstream waste-handling requirements (W_total_: 15.5 vs. 8.5 for Case Studies 2 and 1, respectively, Table 5) along with associated Labor and Cost (L_total_: 3 vs. 2.5 and C_total_: 10 vs. 7 for Case Studies 2 and 1, respectively, Table 5). These factors collectively contributed to the cumulative burden and placed this protocol within the mid-Tier category (TP_Overall_: 28.5, Tier 2, Table 5).

The Tier 3 protocol (Case Study 3) represents the highest operational burden scenario (TP_Overall_ = 31.5; Table 5). The combination of repeated treatment cycles, strong bleaching conditions (use of the most environmentally harmful bleaching chemical, NaClO_2_) with extra stage operational activities (such as pH adjustments), and concentrated sulfuric acid hydrolysis significantly elevated Labor input, Waste generation, and Cost (L_total_: 4.5; Tier 3 and W_total_: 17; Tier 3, C_total_: 10; Tier 2, Table 5). These cumulative parameters resulted in the highest integrated burden score among the examples.

Collectively, these representative case studies illustrate how the rubric can be applied to compare CNC isolation protocols and differentiate their relative assigned operational burdens across the Labor, Waste, and Cost dimensions. The examples demonstrate the applicability of the calculation procedure within the literature-derived scoring system; however, they do not constitute external validation or establish that lower-scoring pathways are inherently greener, more sustainable, or industrially scalable.

### 3.8. Process–Structure–Property Relationships

Extracting pure cellulose from lignocellulosic biomass requires alkaline and bleaching treatments that remove non-cellulosic components of the fiber such as hemicellulose, lignin, pectins, and impurities [15,25,105], thereby increasing the fiber’s cellulose content [4,5,6,7,8]. Alkaline treatment can remove or substantially reduce the hemicellulose content of the lignocellulosic fibers by solubilizing alkali-sensitive hemicellulosic components [106,107,108]. Moreover, alkaline treatment can partially remove lignin from the fiber, with the extent of delignification depending on the biomass source and treatment conditions [42,44,109,110]. In general, alkaline treatment promotes fiber swelling and the partial disruption of the hydrogen-bonding network, improving the surface properties of the resulting fibers [15,111,112]. By altering the crystalline structure of cellulose, weakening the fibers, dissolving amorphous components, and increasing the relative cellulose content, alkali treatment can also affect the mechanical properties of the treated biomass [113,114]. Insufficient alkali treatment may leave residual lignin and hemicellulose, resulting in cellulose with lower purity and crystallinity and a more compact or aggregated fibrillar structure [115]. On the contrary, severe alkali treatment can degrade cellulose by reducing its degree of polymerization and causing fiber shortening, fines formation, and aggregation, thereby inducing structural alterations and transforming cellulose I into cellulose II, which can exhibit greater thermal stability but may have different structural and mechanical properties [43,111,112,116]. Yet, variations in fiber characteristics depend on operating conditions, including the type and concentration of alkali, temperature, and residence time.

Bleaching further purifies the cellulose-rich fraction by removing residual lignin and lignin-associated components, thereby generally increasing cellulose purity and whiteness before hydrolysis [117,118,119]. Bleaching can modify fiber morphology, crystallinity, surface structure, thermal stability, and mechanical properties; however, the magnitude and direction of these changes depend on the bleaching agent and treatment severity [118,120,121]. Increasing oxidant concentration, bleaching duration, or the number of bleaching stages can improve delignification and color removal. However, increasingly severe bleaching may become less selective, causing additional cellulose and hemicellulose loss, reducing the recovered cellulose yield, and potentially altering the properties of the obtained cellulose [117,118,122]. Conversely, inadequate bleaching may leave more residual lignin and produce cellulose with a higher kappa number and poorer color [118].

Acid hydrolysis is the process of producing cellulose nanocrystals from cellulose-rich biomass by preferentially attacking the more accessible, less ordered regions of cellulose (the amorphous region) while largely preserving the crystalline domains [23]. The operational conditions employed during acid hydrolysis, particularly reaction time, temperature, acid type and concentration, directly affect the properties of the resulting CNCs, including CNC yield, crystallinity, particle dimensions, surface functionality, colloidal stability, and thermal behavior [123,124,125,126,127,128,129,130]. For example, regarding particle dimensions, more severe acid hydrolysis conditions have been reported to shorten CNCs by promoting further cleavage of cellulose fibrils; however, their effect on aspect ratio is not consistently monotonic because length and transverse dimensions may change at different rates [28,131,132,133]. In this regard, Jo et al. [41] found that increasing sulfuric acid concentration, temperature, and reaction time reduced CNC length, whereas width changed comparatively little after individual particles were liberated; consequently, the aspect ratio of paper-mulberry CNCs generally decreased. In contrast, Kassab et al. [28] reported that increasing the sulfuric acid hydrolysis time from 10 to 30 min reduced the mean CNC length from 410 to 367 nm and the diameter from 10.2 to 7.4 nm, thereby increasing the aspect ratio from 40 to 49. Thus, shortening alone does not necessarily imply a lower aspect ratio. Gallardo-Sánchez et al. [132] likewise demonstrated that CNC dimensions depended interactively on acid identity, acid concentration, temperature, and hydrolysis time. At 60 wt% H_2_SO_4_ and 40 °C, extending hydrolysis from 40 to 70 min reduced the length-to-height ratio from approximately 43 to 23; increasing H_2_SO_4_ concentration from 60 to 65 wt% at 40 °C and 40 min also reduced it from approximately 43 to 24. Nevertheless, other combinations produced non-monotonic responses, showing that the individual effects of concentration, temperature, and time cannot always be interpreted independently. CNCs produced with HCl displayed a more variable dimensional response and were generally longer and wider than those produced with H_2_SO_4_ [132].

The type of acid also determines the CNC surface chemistry. During sulfuric acid hydrolysis, esterification of cellulose surface hydroxyl groups introduces sulfate half-ester groups, thereby giving the CNCs a negative surface charge and improving the dispersion and stability of aqueous CNC suspensions [15,25,26]. However, at elevated temperatures, surface sulfate groups can dissociate, releasing sulfuric acid that catalyzes cellulose depolymerization [45], thereby reducing CNC thermal stability [45,48,134,135]. In comparison, phosphoric-acid hydrolysis introduces phosphate groups onto the CNC surface. The resulting phosphorylated CNCs generally exhibit lower surface charge and aqueous dispersibility than sulfuric-acid-derived CNCs but greater thermal stability [28,134].

Organic acid hydrolysis can generate CNCs with carboxyl- or other acid-derived surface functionalities [136,137,138]. These functionalities modify the surface charge, dispersion behavior, chemical reactivity, and thermal stability compared with conventionally sulfated CNCs (S-CNCs). Accordingly, in comparison with S-CNCs, organic-acid-derived CNCs generally exhibit lower surface charge but can retain satisfactory aqueous dispersibility and show improved thermal stability [45,136,139], yield, and crystallinity [140,141,142]. However, organic acid hydrolysis generally requires higher reaction temperatures and longer processing times owing to their lower acidity [136,139]. In all cases, isolation yield and the properties of the resulting CNCs depend on the cellulose source, acid concentration, reaction time, and reaction temperature [48,136]. Overall, the effects of individual hydrolysis variables cannot be reliably predicted independently because acid identity, concentration, temperature, and reaction time can interact to produce nonlinear and sometimes opposing changes in CNC characteristics. Consequently, CNC properties should be interpreted in relation to the complete set of hydrolysis conditions, together with cellulose source and pretreatment history, rather than attributed to any single operational variable.

Feedstock origin and the nature of the starting material represent additional sources of variability in CNC production. Lignocellulosic materials differ in their cellulose, hemicellulose, lignin, extractive and ash contents, as well as in their moisture content, volatile matter, fixed carbon and elemental composition. These characteristics may vary with species, age and growth conditions [143,144]. Such differences can influence both the pretreatment required to obtain sufficiently purified cellulose before hydrolysis and the yield and characteristics of the resulting CNCs [136,145,146]. The studies reviewed here used a wide range of starting materials, including herbaceous biomass and agricultural residues [25,28,126,134], woody biomass [6,129,147], fruit, seed, and shell residue [148,149,150], processed pulps [127,130,151], aquatic biomass [152,153,154], and cellulose and other purified cellulose sources [155,156,157]. Because these materials differ not only in their biological origin but also in their composition and prior processing history, it is difficult to isolate the effect of feedstock type from that of upstream treatment. Feedstock origin and pretreatment history should therefore be regarded as important contextual factors when interpreting relationships between process conditions and product properties. However, they were not included as penalty variables in the present scoring framework because the defined analytical boundary begins with alkaline treatment.

Together, these relationships indicate that process burden alone cannot provide a complete assessment of a CNC isolation protocol, because protocol suitability also depends on feedstock characteristics and the yield and quality of the resulting cellulose and CNCs. A lower-burden protocol is not necessarily the most suitable option if it produces insufficiently purified cellulose, low yields, unsuitable particle dimensions, inadequate surface charge, poor dispersion stability, or insufficient thermal stability. Additional processing may therefore be necessary to achieve the purity, morphology, aspect ratio, surface functionality, colloidal stability, and thermal properties required for a specific application. Moreover, differences among feedstocks may influence treatment requirements and the properties of the resulting cellulose and CNCs. The present rubric should consequently be regarded as a process-burden screening framework, while feedstock characteristics and cellulose and CNC yield and quality metrics represent complementary domains for future integration.

### 3.9. Limitations and Scope of Application

This study was designed as an analytical review and was not conducted as a prospectively registered systematic review; therefore, the search may not have identified every potentially eligible CNC-isolation study. Differences in database coverage, terminology, search rankings, full-text availability, and reporting quality may have introduced selection bias and resulted in some eligible studies being missed. Accordingly, the operational regimes, penalty distributions, and Tier boundaries should be interpreted as patterns derived from the defined 140-study analytical dataset rather than as exhaustive representations of the entire published literature.

The individual penalty values used in the scoring framework are dimensionless ordinal weights designed to represent progressively increasing operational burden across rubric components rather than values established through expert consensus or calibrated against measured labor requirements, waste quantities, or financial costs. Although the operational-regime boundaries and Tier cut-offs were derived from the reviewed dataset, the use of 0.5-point increments was a methodological design decision intended to provide consistent scoring resolution while preserving this ordinal progression. Sensitivity analysis indicated that the principal study rankings and Tier assignments were generally robust to plausible variations in category weights and component penalties, suggesting that the comparative patterns were not unduly dependent on the selected values. Nevertheless, future validation against measured labor, waste, and cost data would strengthen their quantitative interpretation. Because P_33_ and P_67_ were estimated separately from the empirical distributions of the stage-level, category-level, and overall-process scores, the resulting Tier boundaries are specific to the defined 140-study dataset rather than fixed or universal thresholds. These boundaries may shift with changes in dataset composition, particularly for process configurations with scores close to P_33_ or P_67_. Bootstrap analysis indicated that the boundaries were generally stable within the present dataset; however, independent validation using standardized process data remains necessary. The resulting scores and Tiers should therefore be interpreted as relative comparative screening indicators rather than as absolute measures of process performance.

The post-use environmental burden and toxicity rankings provide general comparative guidance rather than universally applicable or site-specific environmental-risk estimates. Their relevance may vary according to local water sources, wastewater treatment technologies, infrastructure, neutralization and biodegradation processes, effluent discharge or reuse practices, receiving environments, exposed organisms, and regulatory requirements. The proposed rankings and assigned scores should therefore be adapted and validated before application to a specific region or production system.

The influence of feedstock origin, composition, physical condition, and pretreatment history was not examined quantitatively. The scoring framework and system boundaries were intentionally defined from the alkaline treatment stage onward, while upstream feedstock-specific operations, such as grinding, dewaxing, pulping, and other pre-treatments, were excluded to reduce bias due to inconsistent starting materials and processing boundaries. Nevertheless, differences in cellulose, lignin, hemicellulose, extractive, and ash contents may affect the required processing severity and the properties of the resulting CNCs. A reliable assessment of these effects would require a separate feedstock-level extraction and analysis that incorporates composition, physical condition, and pretreatment history, which was beyond the scope of the present study.

A complete process-balance and scale-up assessment was not possible because key variables—including dry feedstock input, cellulose-intermediate and CNC yields, reagent and water consumption, washing and neutralization requirements, wastewater volume and composition, energy use, and reagent or water recovery—were inconsistently reported across the reviewed studies. Differences in the processes included within each study, the units and calculation methods used, and the definitions of yield made direct comparisons difficult. Combining separately reported values from these inconsistent studies could therefore have produced misleading results. The current framework evaluates the relative operational burden of reported processing conditions rather than providing a complete mass or energy balance, techno-economic assessment, life-cycle assessment, or industrial-efficiency evaluation. The cost estimates also reflect small-quantity laboratory supplier prices at a specific point in time and do not account for bulk purchasing, regional price variation, transportation, or chemical recovery. A comprehensive assessment would require protocol-level extraction and normalization of standardized material and resource inputs and outputs. Although beyond the scope of the present study, such an assessment represents an important direction for future research and could enable the calculation of process-mass intensity, reagent efficiency, water intensity, energy demand, waste generation per unit of CNC, and recovery efficiency, thereby supporting the further development of the rubric into an industrial scale-up tool.

The proposed rubric has not yet been externally validated using standardized laboratory-, pilot-, or industrial-scale process data. The three case studies presented here illustrate the scoring and classification procedure and should not be interpreted as validation exercises. Independent validation against experimentally measured process-burden indicators and relevant product outcomes is required before the rubric can be used for predictive industrial comparisons or definitive sustainability assessments.

The framework proposed in this study represents a preliminary process-based Tier and scoring system and should not be used alone to identify the optimal protocol, as the framework does not directly incorporate product-performance and quality indicators such as CNC yield, crystallinity, particle dimensions, morphology, surface characteristics, colloidal stability, or thermal properties. Future development could integrate these quality metrics with the Process Tier to balance operational efficiency with material performance, thereby providing a more comprehensive basis for comparing CNC isolation pathways and selecting protocols for specific applications.

## 4. Conclusions

Analysis of the compiled dataset revealed that waste generation represents the dominant contributor to operational burden across CNC isolation protocols, whereas labor usually plays a comparatively smaller role. Considerable stage-specific heterogeneity was also observed, with bleaching treatments exhibiting the greatest variability in operational burden, whereas alkaline treatments showed more standardized practices (i.e., lower variability). These findings identify areas that warrant closer attention in reducing the operational burden of CNC isolation, particularly those associated with waste-generating conditions through more effective waste management strategies.

While previous studies have largely focused on yield, crystallinity, or individual processing steps, structured comparisons of the integrated operational burden of CNC isolation protocols remain limited. In this study, “Stage-Level” and “Category-Level Total Tiers” were introduced to enable consistent cross-study comparison and benchmarking at process, stage, and category levels. Stage-Level Total Tiers reflect the combined Labor, Waste, and Cost burden within each processing stage, while Category-Level Total Tiers track a single burden type (Labor, Waste, or Cost) across stages, enabling identification of protocols dominated by specific operational dimensions. The Overall-Process Tier integrates the scores across the entire CNC isolation process.

Stage-Level Total Tiers aid researchers and practical end users in identifying relatively lower-burden options reported in the literature for each processing stage and prioritize them for further experimental evaluation. Where technically compatible, favorable Stage-Level options may be considered as candidates for developing a hybrid protocol. Similarly, Category-Level Total Tiers can help identify options with comparatively lower Waste, Cost, or Labor scores when a particular operational priority is emphasized.

However, Stage-Level options should not be combined automatically without evaluating their technical compatibility and experimentally testing the resulting process. The framework is therefore intended to support structured screening, comparison, and prioritization of CNC isolation pathways rather than independently identifying an optimal protocol. Its general structure may also provide a basis for evaluating other chemical processing systems, subject to appropriate adaptation.

The proposed framework provides a transparent, data-driven, semi-quantitative tool for preliminary screening and comparison of the relative operational burden of CNC isolation pathways and should be interpreted in light of the limitations discussed earlier. Accordingly, the resulting scores and Tiers represent comparative screening indicators within the reviewed literature and do not necessarily establish that a lower-scoring pathway is more sustainable, scalable, or industrially efficient. The relative rankings and Tier assignments may also vary if different penalty values or category weights are applied. Future research should validate and further develop the framework using independent laboratory-, pilot-, and industrial-scale measurements; comprehensive process-balance data; and feedstock-specific information. Integrating these data with CNC quality metrics, including yield, crystallinity, particle characteristics, surface properties, colloidal stability, and thermal performance, would support the selection of protocols that balance operational efficiency with CNC quality and performance.

## Figures and Tables

**Table 1:** The formula used for unit harmonization of chemical concentrations.

Unit Type	The Formula to Convert to Molarity (M) and Normality (N)
% *w*/*v*	M $=\frac{(\%w/v)\times 10}{MW}$
% *v*/*v*	M $=\frac{(\%v/v\times {\rho \;}_{pure})\times 10}{MW}$
% *w*/*w*	M $=\frac{(\%w/w/100)\times \rho_{\;solution}\times 1000}{MW}$
M	Molarity (mol/L)
N	Normality = M × acidity (number of replaceable H^+^ ions)

MW: molar mass/molecular weight (g); *ρ*: density (g/mL). The factors 10, 100, and 1000 arise from dimensional unit conversion rather than data scaling. Division by 100 converts percentage values to fractions, while multiplication by 1000 converts concentrations expressed on a milliliter basis to a liter basis. For % *w*/*v* and % *v*/*v* concentrations, these conversions simplify to a net factor of 10. These calculations were used to express concentrations reported in different units on a common molarity or normality basis and therefore did not alter the relative variation among observations.

**Table 2:** Integrated framework of literature-derived operational regimes, Scoring Rubric, and Tier classification for CNC isolation process.

Section 1 **: Literature-derived operational regimes of chemical concentrations and experimental variables**											
**CNC Processing Stage**	**Regime**	**Chemicals**		**Concentration Ranges** **(M or N)**		**Cycle Ranges ^1^**	**Concentration-derived Cost Ranges (USD $); Price Category ^2^**		**Temperature Ranges (°C)**	**Time Ranges** **(h or min)**	
Alkaline Treatment	Low	NaOH		<0.78		<2	<2.16; L1		<52.36	≤8.8	
	Moderate	NaOH		0.78–3.16		2–4	2.16–8.74; L2		52.36–89.17	–	
	High	NaOH		>3.16–6.25		>4	>8.74–17.28; L3		>89.17	>8.8	
	Low	KOH		<0.82		<2	<3.79; L1		<56.56	<2.75	
	Moderate	KOH		0.82–1.34		2–4	3.79–6.19; L2		56.56–90	2.75–4.75	
	High	KOH		>1.34–1.78		>4	>6.19–8.22; L2		>90	>4.75	
Bleaching Treatment	Low	NaClO_2_		<0.14		<3	<4.32; L1		<65.69	<1.70	
	Moderate	NaClO_2_		0.14–0.47		3–7	4.32–14.49; L2		65.69–88.22	1.70–3.09	
	High	NaClO_2_		>0.47–1.4		>7	>14.49–43.15; M2		>88.22	>3.09	
	Low	H_2_O_2_		<2.06		<3	<8.77; L2		<59.81	<1.84	
	Moderate	H_2_O_2_		2.06–6.04		3–4	8.77–25.71; L3		59.81–100	1.84–12.92	
	High	H_2_O_2_		>6.04–11.66		>4	>25.71–49.64; M2		>100	>12.92	
	Low	NaClO		<0.59		<2	<50.72; M3		<76.49	<1.72	
	Moderate	NaClO		0.59–0.97		2–4	50.72–83.39; H3		76.49–97.50	1.72–4.86	
	High	NaClO		>0.97–4		>4	>83.39–343.89; VH4		>97.50	>4.86	
Acid Hydrolysis	Low	H_2_SO_4_		<16.01		–	<26.00; L3		<48.59	≤156.25	
	Moderate	H_2_SO_4_		16.01–19.36		–	26.00–31.44; M1		48.59–70	–	
	High	H_2_SO_4_		>19.36–35.93		–	>31.44–58.35; M3		>70	>156.25	
	Low	H_3_PO_4_		<26.02		–	<69.36; H1		<73.75	<120	
	Moderate	H_3_PO_4_		26.02–27		–	69.36–71.97; H2		73.75–100	120–300	
	High	H_3_PO_4_		>27–32.1		–	>71.97–85.57; H3		>100	>300	
Acid Hydrolysis	Low	HCl		<5.42		–	<14.55; L2		<73.69	<146.55	
	Moderate	HCl		5.42–10.15		–	14.55–27.25; L3		73.69–103.57	146.55–493.42	
	High	HCl		>10.15–10.72		–	>27.25–28.78; L3		>103.57	>493.42	
	Low	Formic acid		<14.7		–	<49.83; M2		–	<360	
	Moderate	Formic acid		14.7–25.94		–	49.83–87.93; H3		95 or 100	360–720	
	High	Formic acid		–		–	–		–	> 720	
	Low	Lactic acid		<5.50		–	<46.60, M2		<89.37	<240	
	Moderate	Lactic acid		5.50–6.50		–	46.60–55.07; M3		89.37–109.37	240–300	
	High	Lactic acid		>6.50–7		–	>55.07–59.31; M3		>109.37	>300	
	Low	Citric acid		<7.69		–	<59.69; M3		<79.87	<150	
	Moderate	Citric acid		7.69–14.93		–	59.69–115.90; VH1		79.87–114.72	150–303.51	
	High	Citric acid		>14.93–18		–	>115.90–139.73; VH2		>114.72	>303.51	
Section 2 **: Scoring Framework**											
**Labor**				**Waste**				**Cost**			
**Alkaline Treatment**				**Alkaline Treatment**				**Alkaline Treatment**			
**Variable**	**Chemical**	**Regime**	**Penalty point**	**Variable**	**Chemical**	**Regime**	**Penalty point**	**Variable**	**Chemical**	**Price Category**	**Penalty point**
Time	NaOH/KOH	Low	+0.5	Concentration	KOH	Low	+0.5	Concentration	NaOH/KOH	L1	+0.5
	KOH	Moderate	+1		KOH	Moderate	+1		NaOH/KOH	L2	+1
	NaOH/KOH	High	+1.5		KOH	High	+1.5		NaOH	L3	+1.5
Cycle	NaOH/KOH	Low	+0.5		NaOH	Low	+2	Cycle	NaOH/KOH	Low	+0.5
	NaOH/KOH	Moderate	+1		NaOH	Moderate	+2.5		NaOH/KOH	Moderate	+1
	NaOH/KOH	High	+1.5		NaOH	High	+3		NaOH/KOH	High	+1.5
High pressure	-	-	+0.5	Cycle	NaOH/KOH	Low	+0.5	Temperature	NaOH/KOH	Low	+0.5
Time	H_2_O_2_/NaClO_2_/ NaClO	Low	+0.5	Cycle	NaOH/KOH	Moderate	+1	Temperature	NaOH/KOH	Moderate	+1
	H_2_O_2_/NaClO_2_/ NaClO	Moderate	+1		NaOH/KOH	High	+1.5		NaOH/KOH	High	+1.5
	H_2_O_2_/NaClO_2_/ NaClO	High	+1.5	Temperature	NaOH/KOH	Low	+0.5	High pressure	-	-	+0.5
Cycle	H_2_O_2_/NaClO_2_/ NaClO	Low	+0.5		NaOH/KOH	Moderate	+1	**Bleaching Treatment**			
	H_2_O_2_/NaClO_2_/ NaClO	Moderate	+1		NaOH/KOH	High	+1.5	**Variable**	**Chemical**	**Regime**	**Penalty point**
	H_2_O_2_/NaClO_2_/ NaClO	High	+1.5	High pressure	-	-	+0.5	Concentration	NaClO_2_	L1	+0.5
Post-Bleach Rinse	-	-	+0.5	**Bleaching Treatment**					H_2_O_2_/NaClO_2_	L2	+1
pH/acid	-	-	+0.25	**Variable**	**Chemical**	**Regime**	**Penalty point**		H_2_O_2_	L3	+1.5
pH/base	-	-	+0.25	Concentration	H_2_O_2_	Low	+0.5		-	M1	+2
Extra stage	-	-	+0.5		H_2_O_2_	Moderate	+1		H_2_O_2_/NaClO_2_	M2	+2.5
High pressure	-	-	+0.5		H_2_O_2_	High	+1.5				
**Acid hydrolysis**					NaClO	Low	+2				
**Variable**	**Chemical**	**Regime**	**Penalty point**		NaClO	Moderate	+2.5		NaClO	M3	+3
Time	H_2_SO_4_/H_3_PO_4_ /HCl/Formic/ Lactic/Citric	Low	+0.5		NaClO	High	+3		-	H1	+3.5
	H_2_SO_4_/H_3_PO_4_/HCl/Formic/ Lactic/Citric	Moderate	+1		NaClO_2_	Low	+3.5		-	H2	+4
	H_2_SO_4_/H_3_PO_4_/ HCl/Formic/ Lactic/Citric	High	+1.5		NaClO_2_	Moderate	+4		NaClO	H3	+4.5
High pressure	-	-	+0.5		NaClO_2_	High	+4.5		-	VH1	+5
				**Variable**	**Chemical**	**Regime**	**Penalty point**	**Variable**	**Chemical**	**Regime**	**Penalty point**
				Cycle	H_2_O_2_/NaClO_2_/NaClO	Low	+0.5	Concentration	-	VH2	+5.5
					H_2_O_2_/NaClO_2_/NaClO	Moderate	+1		-	VH3	+6
					H_2_O_2_/NaClO_2_/NaClO	High	+1.5		NaClO	VH4	+6.5
				Temperature	H_2_O_2_/NaClO_2_/NaClO	Low	+0.5	Cycle	H_2_O_2_/NaClO_2_/ NaClO	Low	+0.5
					H_2_O_2_/NaClO_2_/NaClO	Moderate	+1		H_2_O_2_/NaClO_2_/ NaClO	Moderate	+1
					H_2_O_2_/NaClO_2_/NaClO	High	+1.5		H_2_O_2_/NaClO_2_/ NaClO	High	+1.5
				Post-Bleach Rinse	-	-	+0.5	Temperature	H_2_O_2_/NaClO_2_/ NaClO	Low	+0.5
				pH/acid	-	-	+0.25		H_2_O_2_/NaClO_2_/ NaClO	Moderate	+1
				pH/base	-	-	+0.25		H_2_O_2_/NaClO_2_/ NaClO	High	+1.5
				Extra stage	-	-	+0.5	Post-Bleach Rinse	-	-	+0.5
				High pressure	-	-	+0.5	pH/acid	-	-	+0.25
				**Acid hydrolysis**				pH/base	-	-	+0.25
				**Variable**	**Chemical**	**Regime**	**Penalty point**	Extra stage	-	-	+0.5
				Concentration	Lactic acid	Low	+0.5	High pressure	-	-	+0.5
					Lactic acid	Moderate	+1				
					Lactic acid	High	+1.5				
Section 2 **: Scoring Framework**											
**Labor**				**Waste**				**Cost**			
				**Acid hydrolysis (continue)**				**Acid hydrolysis**			
				**Variable**	**Chemical**	**Regime**	**Penalty point**	**Variable**	**Chemical**	**Regime**	**Penalty point**
				Concentration	Formic acid	Low	+2	Concentration	-	L1	+0.5
					Formic acid	Moderate	+2.5		HCl	L2	+1
					Citric acid	Low	+3		HCl/H_2_SO_4_	L3	+1.5
					Citric acid	Moderate	+3.5		H_2_SO_4_	M1	+2
					Citric acid	High	+4		Lactic/Formic	M2	+2.5
					H_2_SO_4_/H_3_PO_4_	Low	+4.5		H_2_SO_4_/Lactic/ Citric	M3	+3
					H_2_SO_4_/H_3_PO_4_	Moderate	+5		H_3_PO_4_	H1	+3.5
					H_2_SO_4_/H_3_PO_4_	High	+5.5		H_3_PO_4_	H2	+4
					HCl	Low	+6		H_3_PO_4_/Formic	H3	+4.5
					HCl	Moderate	+6.5		Citric acid	VH1	+5
					HCl	High	+7		Citric acid	VH2	+5.5
				Temperature	H_2_SO_4_/H_3_PO_4_/HCl/Formic/ Lactic/Citric	Low	+0.5	Temperature	H_2_SO_4_/H_3_PO_4_/ HCl/Formic/ Lactic/Citric	Low	+0.5
					H_2_SO_4_/H_3_PO_4_/HCl/Formic/ Lactic/Citric	Moderate	+1		H_2_SO_4_/H_3_PO_4_ /HCl/Formic/ Lactic/Citric	Moderate	+1
					H_2_SO_4_/H_3_PO_4_/HCl/Formic/ Lactic/Citric	High	+1.5		H_2_SO_4_/H_3_PO_4_/ HCl/Formic/ Lactic/Citric	High	+1.5
				High pressure	-	-	+0.5	High pressure	-	-	+0.5
Section 3 **: Tiering Framework**											
**Stage-level Total Tiers**											
**Alkaline Total (TP_a_)**				**Bleaching Total (TP_b_)**				**Acid hydrolysis Total (TP_c_)**			
**Tiers**	**Percentile range**			**Tiers**	**Percentile range**			**Tiers**	**Percentile range**		
	**P_33_**	**P_67_**			**P_33_**	**P_67_**			**P_33_**	**P_67_**	
	**7.5**	**8.5**			**8.75**	**11.75**			**8.5**	**10**	
Tier 1	Alkaline Total Scores ≤ 7.5			Tier 1	Bleaching Total Scores ≤ 8.75			Tier 1	Acid hydrolysis Total Scores ≤ 8.5		
Tier 2	7.5 < Alkaline Total Scores ≤ 8.5			Tier 2	8.75 < Bleaching Total Scores ≤ 11.75			Tier 2	8.5 < Acid hydrolysis Total Scores ≤ 10		
Tier 3	Alkaline Total Scores > 8.5			Tier 3	Bleaching Total Scores > 11.75			Tier 3	Acid hydrolysis Total Scores > 10		
**Category-level Total Tiers**											
**Labor Total (L_total_)**				**Waste Total (W_total_)**				**Cost Total (C_total_)**			
**Tiers**	**Percentile range**			**Tiers**	**Percentile range**			**Tiers**	**Percentile range**		
	**P_33_**	**P_67_**			**P_33_**	**P_67_**			**P_33_**	**P_67_**	
	**3.25**	**4**			**14.25**	**16**			**9**	**10.25**	
Tier 1	Labor Total Scores ≤ 3.25			Tier 1	Waste Total Scores ≤ 14.25			Tier 1	Cost Total Scores ≤ 9		
Tier 2	3.25 < Labor Total Scores ≤ 4			Tier 2	14.25 < Waste Total Scores ≤ 16			Tier 2	9 < Cost Total Scores ≤ 10.25		
Tier 3	Labor Total Scores > 4			Tier 3	Waste Total Scores > 16			Tier 3	Cost Total Scores > 10.25		
**Overall Process Total Tier (TP_total_)**											
				**Tiers**	**Percentile range**						
					**P_33_**	**P_67_**					
					**26.25**	**29.75**					
				Tier 1	Overall Process Total Scores ≤ 26.25						
				Tier 2	26.25 < Overall Process Total Scores ≤ 29.75						
				Tier 3	Overall Process Total Scores > 29.75						

1: Acid hydrolysis stage applied using a single cycle throughout the dataset; 2: L1: Low 1 ($0–5); L2: Low 2 ($5–15); L3: Low 3 ($15–30); M1: Medium 1 ($30–40); M2: Medium 2 ($40–50); M3: Medium 3 ($50–60); H1: High 1 ($60–70); H2: High 2 ($70–80); H3: High 3 ($80–90); VH1: Very High 1 ($90–120); VH2: Very High 2 ($120–150); VH3: Very High 3 ($150–250); VH4: Very High 4 ($250–350); E: Extreme ($ > 350).

**Table 3:** Summary of the four sensitivity and robustness analyses.

Test	Perturbation	Overall-Process Tier Stability	Spearman ρ
Category weights (±25%, OAT)	Labor/Waste/Cost	91.5–100%	0.995–0.999
Penalty spacing—compressed	regime/conc/both	100%	1.00
Penalty spacing—expanded	regime/conc/both	86.7–93.6%	0.960–0.977
Monte-Carlo—category weights (10,000)	U (0.75, 1.25)	97.7% (mean)	0.997 (mean)
Monte-Carlo—per-penalty (5000)	U (0.75, 1.25)	83.5% (mean)	—
Bootstrap boundaries (2000)	resampling	medians = published	—

**Table 4:** Bootstrap medians and 95% intervals of the percentile-based Tier boundaries (2000 resamples).

Metric	Published P_33_	Bootstrap P_33_ [95% CI]	Published P_67_	Bootstrap P_67_ [95% CI]
TP_a_ (alkaline)	7.5	7.5 [7.5–7.5]	8.5	8.5 [8.5–8.5]
TP_b_ (bleaching)	8.75	8.75 [8.75–9.75]	11.75	11.75 [11.25–12.25]
TP_h_ (acid hydrolysis)	8.5	8.5 [8.5–8.5]	10	10 [10–10]
L_total_	3.25	3.25 [3.25–3.25]	4	4 [4–4]
W_total_	14.25	14.25 [13.5–14.48]	16	16 [15.75–16]
C_total_	9	8.75 [8.75–9.25]	10.25	10.25 [10–10.5]
TP_Overall_	26.25	26.25 [25.75–27.25]	29.75	29.75 [29.42–30.27]

**Table 5:** Illustrative application of the practical Rubric system to classify CNC isolation processing stages and the entire process from Labor, Waste, and Cost perspectives through representative examples of the reviewed literature. Refer to Table 2 for the regime or price category corresponding to each experimental variable (values) and the related assigned penalty points.

Experimental Variables	Case Study 1	Labor Points	Waste Points	Cost Points	Case Study 2	Labor Points	Waste Points	Cost Points	Case Study 3	Labor Points	Waste Points	Cost Points
Alkaline chemical	NaOH	-	-	-	NaOH	-	-	-	NaOH	-	-	-
Alkali concentration (M)	1	NA	+2.5	+1	5.214	NA	+3	+1.5	1.043	NA	+2.5	+1
Operational temperature (°C)	80	NA	+1	+1	80	NA	+1	+1	80	NA	+1	+1
Operational time (h)	1	+0.5	NA	NA	2	+0.5	NA	NA	2	+0.5	NA	NA
Number of cycles	1	+0.5	+0.5	+0.5	1	+0.5	+0.5	+0.5	4	+1	+1	+1
Utilizing high-pressure vessels	NO	0	0	0	NO	NO	0	0	NO	0	0	0
Stage-level (Total) Scores (Alkaline)	(TP_a_: 7.5)	L_a_: 1	W_a_: 4	C_a_: 2.5	(TP_a_: 8.5)	L_a_: 1	W_a_: 4.5	C_a_: 3	(TP_a_: 9)	L_a_: 1.5	W_a_: 4.5	C_a_: 3
Stage-level Total Tier (Alkaline)	Tier 1				Tier 2				Tier 3			
Bleaching chemical	H_2_O_2_	-	-	-	NaClO	-	-	-	NaClO_2_	-	-	-
Bleach concentration (M)	1	NA	+0.5	+1	0.273	NA	+2	+3	0.189	NA	+4	+1
Operational temperature (°C)	80	NA	+1	+1	70	NA	+0.5	+0.5	80	NA	+1	+1
Operational time (h)	1	+0.5	NA	NA	1	+0.5	NA	NA	4	+1.5	NA	NA
Number of cycles	1	+0.5	+0.5	+0.5	1	+0.5	+0.5	+0.5	2	+0.5	+0.5	+0.5
Post-bleach rinse using reducing salt	NO	0	0	0	NO	0	0	0	NO	0	0	0
pH adjustment using acidic additives	NO	0	0	0	NO	0	0	0	YES	+0.25	+0.25	+0.25
pH adjustment using basic additives	NO	0	0	0	NO	0	0	0	YES	+0.25	+0.25	+0.25
Extra stage using specific chemicals	NO	0	0	0	NO	0	0	0	NO	0	0	0
Utilizing high-pressure vessels	NO	0	0	0	NO	0	0	0	NO	0	0	0
Stage-level (Total) Scores (Bleaching)	(TP_b_: 5.5)	L_b_: 1	W_b_: 2	C_b_: 2.5	(TP_b_: 8)	L_b_: 1	W_b_: 3	C_b_: 4	(TP_b_: 11.5)	L_b_: 2.5	W_b_: 6	C_b_: 3
Stage-level Total Tier (Bleaching)	Tier 1				Tier 1				Tier 2			
Acid hydrolysis chemical	H_2_SO_4_	-	-	-	HCl	-	-	-	H_2_SO_4_	-	-	-
Acid concentration (N)	6	NA	+4.5	+1.5	1	NA	+6	+1	22.99	NA	+5.5	+3
Operational temperature (°C)	40	NA	+0.5	+0.5	110	NA	+1.5	+1.5	50	NA	+1	+1
Operational time (min)	60	+0.5	NA	NA	60	+0.5	NA	NA	60	+0.5	NA	NA
Utilizing high-pressure vessels	NO	0	0	0	YES	+0.5	+0.5	+0.5	NO	0	0	0
Stage-level (Total) Scores (Hydrolysis)	(TP_h_: 7.5)	L_h_: 0.5	W_h_: 5	C_h_: 2	(TP_h_: 12)	L_h_: 1	W_h_: 8	C_h_: 3	(TP_h_: 11)	L_h_: 0.5	W_h_: 6.5	C_h_: 4
Stage-level Total Tier (Hydrolysis)	Tier 1				Tier 3				Tier 3			
Category-level Total Scores		L_total_: 2.5	W_total_: 11	C_total_: 7		L_total_: 3	W_total_: 15.5	C_total_: 10		L_total_: 4.5	W_total_: 17	C_total_: 10
Category-level Total Tier		Tier 1	Tier 1	Tier 1		Tier 1	Tier 2	Tier 2		Tier 3	Tier 3	Tier 2
Overall Process Total Scores	TP_Overall_: 20.5				TP_Overall_: 28.5				TP_Overall_: 31.5			
Overall Process Total Tier	Tier 1				Tier 2				Tier 3			

## Data Availability

The original contributions reported in this study are provided within the article and the Supplementary Materials. Any further inquiries may be directed to the corresponding authors.

## Supplementary Materials

The following supporting information can be downloaded at: https://www.mdpi.com/article/10.3390/molecules31173042/s1, Supplementary Text: Methods: Study Selection Criteria; Dataset Preparation; Data Analyses and Operational Regimes Computation; Ranking Chemicals According to Price; Ranking Chemicals According to Environmental Friendliness; CNC Scoring and Tiering Rubric: A Practical Guide; Sensitivity Analysis and Robustness of the Scoring Framework; Illustrative Application of the Rubric; Figure S1: Literature identification and screening flow used to construct the analytical dataset; Figure S2: Literature-derived Gaussian Mixture Model (GMM) outputs for experimental parameters of alkaline treatments used during the CNC isolation process: (a–d) NaOH treatments. (e–h) KOH treatments, (a,e) alkaline concentration (molarity), (b,f) operational temperature (°C), (c,g) reaction time (h), (d,h) number of cycles; Figure S3: Literature-derived Gaussian Mixture Model (GMM) outputs for experimental parameters of the bleaching treatments used during the CNC isolation process: (a–d) H_2_O_2_ treatments, (e–h) NaClO treatments, (i–l) NaClO_2_ treatments, (a,e,i) bleach concentration (molarity), (b,f,j) operational temperature (°C), (c,g,k) reaction time (h), (d,h,l) number of cycles; Figure S4: Literature-derived Gaussian Mixture Model (GMM) outputs for acid concentration (expressed as normality) used in acid hydrolysis of the CNC isolation process. (a) Sulfuric acid. (b) Phosphoric acid. (c) Hydrochloric acid. (d) Formic acid. (e) Lactic acid (f) Citric acid; Figure S5: Literature-derived Gaussian Mixture Model (GMM) outputs for operational temperature (°C) used in acid hydrolysis of the CNC isolation process. (a) Sulfuric acid. (b) Phosphoric acid. (c) Hydrochloric acid. (d) Formic acid. (e) Lactic acid. (f) Citric acid; Figure S6: Literature-derived Gaussian Mixture Model (GMM) outputs for reaction time (min) used in acid hydrolysis of the CNC isolation process. (a) Sulfuric acid. (b) Phosphoric acid. (c) Hydrochloric acid. (d) Formic acid. (e) Lactic acid. (f) Citric acid; Table S1: List of studies included in the current review (n = 140); Table S2: Estimated (bold) and tabulated (normal font) density values correspond to different chemical concentrations. Tabulated density data were obtained from references 1 to 4 and the link provided below the table; Table S3: The price of the chemical from two reputable suppliers, Sigma-Aldrich and Fisher Scientific. Items in blue were chosen for the comparison and to select the lowest comparable quoted unit price (Table S5); Table S4: The selected chemicals with their corresponding prices (per kilogram or liter in US dollars) from the two reputable suppliers, Sigma-Aldrich and Fisher Scientific. The final selected chemicals based on the best prices (lowest comparable quoted unit price) are shown in bold; Table S5: Calculated prices for different concentration distribution ranges of each chemical across three stages of the CNC isolation process. Reagent purity was considered in all price calculations; Table S6: Price categories and corresponding penalty points for different concentration distribution ranges of each chemical across three stages of the CNC isolation process. Reagent purity was considered in all price calculations; Table S7: Post-use environmental burden of the acids in wastewater streams. Please refer to Tables S8–S10 for Representative SDS human hazard classes for each chemical; Table S8: Measured values from standard environmental and animal (in vivo) toxicological tests for alkaline chemicals. Toxicity endpoints compared between the two alkaline chemicals are shown in bold; Table S9: Measured values from standard environmental and animal (in vivo) toxicological tests for bleaching chemicals. Toxicity endpoints compared across different bleaching agents are shown in bold; Table S10: Measured values from standard environmental and animal (in vivo) toxicological tests for organic and inorganic acids. Toxicity endpoints compared across different acids are shown in bold; Table S11: Acute toxicity of acids to different fish species and relative toxicity ranking; Table S12: Results of ecotoxicity tests and regulatory guidelines for inorganic ions derived from alkaline and bleaching chemicals. Comparable values are shown in the same color. Data were obtained from references 55 to 63 and the related links provided below the table; Table S13: Results of ecotoxicity tests and regulatory guidelines for inorganic ions derived from mineral acids. Comparable values are shown in the same color. Data were obtained from references 64 to 69 provided below the table; Table S14: Summary of the chemical penalty points listed in Tables S7–S13 and the sum of the total penalty points used for the overall ranking of the chemicals for environmental friendliness from a waste perspective.
