# Supplementary material for: A Data-Driven Semi-Quantitative Framework for Comparative Screening and Tiering of Cellulose Nanocrystal Isolation Pathways: Implications for Process Assessment and Future Optimization
Source: Molecules. 2026 Aug 29;31(17):3042. doi: 10.3390/molecules31173042 (PMC13566977; doi:10.3390/molecules31173042)
Supplement: Supplementary file 1 [file molecules-31-03042-s001.zip › molecules-4448643-supplementary.pdf]

Supplementary Materials

A Data-Driven Semi-Quantitative Framework for Comparative Screening and Tiering of Cellulose Nanocrystal Isolation Pathways: Implications for Process Assessment and Future Optimization

Atefeh Esmaeili, Farzin Shabani, Mohammad A. Al-Ghouti*

Department of Biological and Environmental Sciences, College of Arts and Sciences, Qatar University, P.O. Box 2713, Doha, Qatar

*Corresponding author. Email: mohammad.alghouti@qu.edu.qa

List of contents:

Methodology details include:
1. Study Selection Criteria
2. Dataset Preparation
3. Data Analyses and Operational Regimes Computation
4. Ranking Chemicals According to Price, including examples of price calculations
5. Ranking Chemicals According to Environmental Friendliness
6. CNC Scoring and Tiering Rubric: A Practical Guide
7. Sensitivity Analysis and Robustness of the Scoring Framework
8. Illustrative Application of the Rubric

Figs. S1 to S6
Tables S1 to S14
References

Supplementary Text

2.2. Methods

2.2.1. Study Selection Criteria

This study was conducted as a structured analytical review rather than a systematic review. The literature-identification procedure was designed to construct a well-defined analytical dataset containing sufficiently detailed experimental information to characterize the operational conditions used during CNC isolation.

Relevant studies published between 2015 and 2025 were initially identified through Google Scholar using the following search terms individually and in combination: “cellulose nanocrystal extraction,” “nanocrystalline cellulose extraction,” “cellulose nanocrystal isolation,” “nanocrystalline cellulose isolation,” “CNC extraction,” “CNC isolation,” “NCC extraction,” and “NCC isolation.” These searches were supplemented using Harzing’s Publish or Perish software, software (Windows GUI version 8.12.4612.8838; Harzing.com), with Crossref as the underlying bibliographic source. The recovered Publish or Perish export comprised two batches of 1,000 records retrieved on 1st and 2nd October 2025. Studies previously identified through Google Scholar but not returned by the supplementary Crossref searches were retained in the candidate dataset.

The search outputs and previously identified studies were consolidated into a screening file containing 886 candidate records. Records were classified according to their relevance and the availability of the experimental information required for the analysis. Duplicate entries were identified through comparison of study titles, authors, and, where available, DOI information.

To be included, a study was generally required to report sufficiently detailed experimental procedures for the CNC-isolation workflow, including all three main stages of alkaline treatment, bleaching

treatment, and acid hydrolysis, and provide sufficient quantitative characterization of the resulting CNC product to demonstrate the protocol's effectiveness. Studies specifically designed to investigate or optimize an individual alkaline treatment, bleaching, or hydrolysis stage were eligible exceptions when they reported sufficient quantitative information for the target stage. Records were excluded when “CNC” referred to computer numerical control rather than cellulose nanocrystals; when the publication was unrelated to CNC extraction or isolation; when it focused on nanofibrillated cellulose, microfibrillated cellulose, or CNC-containing products rather than CNC isolation; when it employed a processing route outside the defined acid-hydrolysis-based analytical boundary; when it was a review rather than a primary experimental study; or when the full text or required quantitative procedural information was unavailable.

Each publication was treated as an individual study. A study could include multiple experimental trials when evaluating different feedstocks, chemicals, concentrations, temperatures, reaction times, treatment cycles, or optimization conditions. Following screening and consolidation of duplicate records, the final analytical dataset comprised 140 unique studies and 686 experimental trials. The included studies are listed in Supplementary Table S1, and the screening process is shown in Figure S1.

2.2.2. Dataset Preparation

For clarity, a stage refers to one of the three main experimental stages involved in the entire CNC isolation process: alkaline treatment, bleaching treatment, or acid hydrolysis. An operational variable is a reported process parameter or experimental condition, such as chemical concentration, temperature, processing time, or number of cycles. The distribution range describes the observed values of an operational variable across the reviewed dataset, whereas an operational regime is a literature-derived interval for an operational variable, classified as Low, Moderate, or High for each

chemical type. Operational intensity refers to the relative processing severity indicated by the Low, Moderate, or High regimes of individual operational variables or by their combined conditions. Operational burden refers to the aggregated penalty points within a category, stage, or overall CNC-isolation process. A penalty point is a dimensionless ordinal weight assigned within a scoring category, namely Labor, Waste, or Cost. A Tier is a final percentile-based class (Tier 1–3) derived from the operational burden at the category, stage, or overall-process level. These terms are used consistently throughout the manuscript.

A comprehensive dataset was prepared by extracting all data related to the CNC isolation process from the reviewed literature and classifying it into three experimental stages (alkaline treatment, bleaching treatment, and acid hydrolysis; referred to as "stages" throughout this manuscript). The data included detailed experimental conditions (variables), such as chemical type, chemical concentration, concentration unit, experimental temperature, operating time, and number of repeated cycles. After data extraction, attempts were made to harmonize the data across the dataset, in particular for chemical concentrations, for which the majority of values were inconsistently reported using a variety of % units, including mass percent (% w/w), weight/volume percent (% w/v), and volume percent (% v/v), with molarity (M), being reported occasionally for some acids. Therefore, to enable a more reasonable comparison of concentrations within each chemical type across the dataset, the concentrations of alkaline and bleaching chemicals were converted to molarity, and the acid concentrations were converted to normality using the reported % units. For cases where % units were reported as % w/w and % v/v, the density values of the operational working solution and the stock solution were needed, respectively, for calculation. The details of the concentration unit conversion formula are provided in Table 1. The factors 10, 100, and 1000 in Table 1 arise from dimensional unit conversion rather than data scaling. Division by 100 converts percentage values to fractions, while multiplication by 1000

converts concentrations expressed on a milliliter basis to a liter basis. For % w/v and % v/v concentrations, these conversions simplify to a net factor of 10. These calculations were used to express concentrations reported in different units on a common molarity or normality basis and therefore did not alter the relative variation among observations. As shown in Table 1, ρ_{pure} denotes the density of the pure chemical (i.e., the stock solution provided by suppliers — for example, sulfuric acid 98% or phosphoric acid 85%) and ρ_{solution} refers to the density of the prepared chemical solution used in the experiment (for example, sulfuric acid 64%, which was prepared by diluting sulfuric acid 98%). All density values were obtained from standard chemistry references [1,2]. Density figures for citric acid with concentrations greater than 30% w/w were taken from Żarska, *et al.* [3]. The density data for NaClO were obtained online [4], whereas $NaClO_2$ density values were provided by Occidenta Chemical Corporation OxyChem [5]. All the values were extracted at 20 °C, except H_2O_2 and $NaClO_2$, for which the values were only available at 18 °C and 25°C, respectively. When chemical concentrations (%w/w) in the dataset contained decimal values (e.g., sulfuric acid 62.4% w/w), which fall between the tabulated ranges of available density values, the density of the solution (ρ solution) was estimated by linear interpolation between the two nearest tabulated values (e.g., 62% and 63% w/w). If the value was outside the available range, linear extrapolation was applied from the closest tabulated data points. The same formula was employed for both interpolation and extrapolation as presented below:

$$\rho(x) = \rho(x_1) + (x - x_1)\frac{\rho(x_2) - \rho(x_1)}{x_2 - x_1} \quad (1)$$

Where $\rho(x)$ represents the estimated density of the desired concentration, and $\rho(x_1)$ and $\rho(x_2)$ are densities corresponding to the two nearest known concentrations.

When x lies between x_1 and x_2, the estimation corresponds to interpolation (within the known range).

When x lies outside the known range ($x < x_1$ or $x > x_2$), the estimation corresponds to extrapolation. The estimated density values corresponding to different chemical concentrations are listed in Table S2.

2.2.3. Data Analyses and Operational Regimes Computation

After normalizing chemical concentrations and harmonizing experimental durations (time), the data were cleaned prior to analysis. Statistical analyses, including tests of normality and skewness, were conducted, and numerical variables were visualized through histograms and density plots, followed by estimation of Kernel Density functions (Estimates; KDE). In addition, correlation analyses (correlation matrix) among the experimental parameters/variables (concentration, temperature, time, and cycle) were performed for each of the three CNC isolation stages: alkaline and bleaching treatments and acid hydrolysis, with $|r| = 0.8$ being used as a prespecified screening threshold for very high pairwise correlation [6]. The distribution ranges (operational regimes) of each experimental variable were extracted and analyzed using Gaussian Mixture Models (GMMs) implemented in the Mclust R package [7] to identify distinct distributional patterns (operational regimes) and potential subgroups within each chemical category in each stage. Hence, for each stage, data were categorized by chemical type, and mixture models were fitted separately for each variable within each category. Variables with inadequate sample size ($N < 3$) or negligible variability (standard deviation < 0.001) were excluded from mixture modelling to control for unstable or non-informative fits. GMMs with one to four components were fitted for each valid subset, with the Bayesian Information Criterion (BIC) being employed to select the optimum number of components and covariance structure. The BIC delivers a likelihood-based measure of model fit, penalizing extreme model complexity, with lower BIC values representing stronger support for a given model compared to alternatives. The number of mixture components (modes), component means, standard deviations, and mixing

proportions were extracted for each selected model. Mixing proportions denote the relative frequency (occurrence) of observations belonging to each detected component and thus indicate the prevalence of different operational regimes reported in the literature. For ease of interpretation, Kernel Density Estimates (KDEs) of the empirical distributions were produced for each variable and chemical type, with the estimated mixture-component means shown by vertical dashed lines. These visual features were used to verify that the detected modes corresponded to and reflected the real structure in the original data rather than to model artifacts. In particular, the identified mixture components were interpreted as operational regimes reflecting common experimental practices rather than as hidden physicochemical processes.

To obtain operational regimes (Low/Moderate/High) or distribution ranges for scoring purposes, thresholds derived from the mixture model were combined with robust distribution-based safety checks to ensure reliable classification. Cleaned and harmonized datasets were summarized to attain the first quartile (Q1), third quartile (Q3), and maximum values for each process variable (e.g., concentration, temperature, time, and number of cycles) within each stage (alkaline and bleaching treatments, and acid hydrolysis) and chemical type. At the same time, previously fitted GMMs were used to detect potential multimodal structure (multiple patterns) in each variable, and the selected model structure and component means were recorded as outputs. Mixture component means were initially converted into numeric vectors and sorted in ascending order, with candidate cut points being defined as midpoints between chosen component means. The first threshold (c_1) was set as the midpoint between the two lowest component means when at least two components were present. The second threshold (c_2) was set as the midpoint between the second and third component means when three components were available. When four or more components were recognized, c_2 was deliberately taken from the upper tail of the distribution (midpoint between the third and fourth means) to yield a

more conservative definition of the high-intensity regime and to avoid over-assigning observations to the high category.

When mixture separation was weak or poorly defined, fallback thresholds were applied to maintain stability. The empirical third quartile (Q3) was substituted for c_1 when c_1 could not be derived from the mixture model. When c_2 was not available, it was replaced by a robust upper-bound threshold defined as the minimum of the upper fence (Q3 + 1.5 × Interquartile Range (IQR), where IQR = Q3 − Q1) and the observed maximum. Lastly, threshold validity was imposed: when c_2 was not clearly larger than c_1, the variable was divided into two regimes (split at c_1) if the maximum exceeded c_1; otherwise, it was treated as a single fixed regime with no meaningful split.

2.2.4. Ranking Chemicals According to Price

Chemical prices were calculated based on the cost of preparing 1 L of solution for each chemical, corresponding to the literature-derived concentration range distributions (operational regimes). Initially, chemical market prices were extracted from two reputable and widely recognized suppliers: 1) Sigma-Aldrich [8] and 2) Fisher Scientific [9]. Among the various available options for each chemical in terms of purity and other specifications, the most commonly used options were selected and listed in Table S3. From these, comparable options in terms of purity and concentration were chosen from both suppliers for price comparison. Particular attention was given to products for which the price was available per liter or kilogram of chemical, as shown in blue in Table S3. Finally, the prices were unified in US dollars (Table S4), and the best deal for each chemical was selected and considered as the chemical's price for further calculation (shown in bold in Table S4). Thereafter, the final price of each chemical was calculated based on the cost of preparing one liter of solution according to previously identified concentration distribution ranges/operational regimes using the chosen chemicals' price (bold items in Table S4) and listed in Table S5. Reagent grade and stock

purity were incorporated into the calculations. For each supplier product, the amount required to prepare 1 L of working solution was corrected using the listed stock concentration or purity (for example, dividing the required pure-solute mass by the corresponding purity fraction). The resulting estimates therefore represent laboratory-scale catalog costs and should not be interpreted as bulk industrial prices. The calculated prices were then used as the basis for comparison among different chemicals within each stage (alkaline chemicals, bleaching agents, and acids). Chemicals belonging to each CNC stage were then categorized by calculated price. To do that, chemical costs were categorized using unified study-wide (cross-chemical-type) price categories defined based on the observed distribution of prices across all alkaline, bleaching, and acid treatments in the dataset: Low (US \$ 0–30), Medium (US \$ > 30–60), High (US \$ > 60–90), Very High (US \$ > 90–350), and Extreme (US \$ > 350)). Each category was then further divided into smaller sub-categories to differentiate the price span of the chemicals within the same study-wide price category (Table S6). To ensure consistent and conservative penalty assignment, each cost range was assigned to a single category using worst-case logic: for upper-only open ranges (< x), the upper bound (x) was used for categorization, representing the maximum possible cost within the range. For bounded ranges (a–b), the upper bound (b) was used as the worst-case figure. And finally, for lower-only open ranges (> x), to facilitate price categorization, the maximum concentration values for each chemical were extracted from the dataset's summary statistics to set the upper bound and determine the price with a specific ceiling. This approach ensures transparent, reproducible, and penalty-consistent cost classification across all chemicals, and the provided calculation formula and categories can be universally employed by researchers and industrial end users to categorize target chemicals in a price-wise manner. The resulting price categories and corresponding penalty points were presented in Table S6 and used later to develop the CNC Scoring and Tiering Rubric from a cost perspective. Examples of the price calculation for

different types of chemicals (solid chemicals, bleaching agents, solid acids, and liquid acids) according to the supplier's prices (per Kg or L) listed in Table S5 are provided below (reagent stock purity was considered in all calculations):

Performed calculation for solid chemicals: NaOH, KOH, and $NaClO_2$ (Example: NaOH):

NaOH (≥ 98% w/w): Target concentration: 3.16 M; Molar mass (Molecular weight: MW): 39.997 g/mol; Purity: 0.98; Price per Kg: $67.74

Required mass (g) to prepare 1 liter of NaOH 3.16 M = 3.16 × 39.997 = 126.391 g

Correcting for stock purity: = 126.391/0.98 = 128.97 g

Cost = 67.74 × (128.97/1000) = $8.74

Performed calculation for Solid acids (Example: Citric acid):

Citric acid (99% w/w): Target concentration: 7.69 N; MW: 192.124 g/mol, Equivalent weight = MW/3 = 64.041 g/eq; Purity: 0.99; Price per Kg: $120

Required mass (g) to prepare 1 liter of 7.69 N = 7.69 × 64.041 = 492.478 g

Correcting for stock purity: = 492.478/0.99 = 497.452 g

Cost = 120 × (497.452/1000) = $59.69

Performed calculation for liquid bleaching agents (H_2O_2 & NaCLO):

H_2O_2 (30 % w/w): Target concentration: 2.06 M; MW: 34.015 g/mol; Purity: 0.30; ρ: 1.1122 g/mL; Price per L: $41.76

Mass of 1 Liter stock = 1.1122 × 1000 = 1112.2 g

Mass of H_2O_2 in 1 Liter stock solution (Correcting for purity) = 0.30 × 1112.2 = 333.66 g

Moles of H_2O_2 in 1 Liter stock solution = 333.66/34.015 = 9.809 = Stock molarity (C_1)

Required volume (V_1 based on mL) to prepare 1 Liter of 2.055 M H_2O_2 solution using $C_1V_1 = C_2V_2$ equation: V_1 = (2.06 × 1000)/9.809 = 210.01 mL

Cost = 41.76 × (210.01/1000) = \$8.77

NaClO (10–15% w/w available chlorine): Target concentration: 0.59 M; MW: 74.442 g/mol; ρ: 1.194 g/mL; Price per L: \$180.96

The average chlorine content was considered for calculation (12.5 % w/w)

Converting 12.5 % w/w available chlorine to %w/w NaClO:

%w/w NaClO = % available chlorine × MW NaClO/MW available chlorine = 12.5 × 74.442/70.906 = 13.123%

Mass of 1 Liter stock = 1.194 × 1000 = 1194 g

Mass of NaClO in 1 Liter stock solution (Correcting for purity) = 0.13123 × 1194 = 156.689 g

Moles of NaClO in 1 Liter stock solution = 156.689 /74.442 = 2.105 = Stock molarity (C_1)

Required volume (V1 based on mL) to prepare 1 Liter of 0.59 M NaClO solution using $C_1V_1 = C_2V_2$ equation: V_1 = (0.59 × 1000)/2.105 = 280.306 mL

Cost = 180.96 × (280.306/1000) = \$50.72

Performed calculation for liquid acids (H_2SO_4, H_3PO_4, HCl, Formic acid, & lactic acid, Example: H_2SO_4):

H_2SO_4 (95–97% w/w): Target concentration: 16.01 N; MW: 98.079 g/mol; Number of replaceable Hydrogen (n): 2; Purity: 0.96 on average; ρ: 1.8355 g/mL; Price per Kg: \$58.35

Stock Normality = (ρ × purity × n × 1000) /MW = (0.96 × 1.8355 × 1000 × 2)/98.079 = 35.932 N

Required volume to prepare 1 Liter of 16.01 N using $C_1V_1 = C_2V_2$ equation: V_1 = (16.01 × 1000)/35.932 445.57 mL

Cost = 58.35 × (445.57/1000) = \$26.00

2.2.5. Ranking Chemicals According to Environmental Friendliness

Toxicity and ecotoxicity of the Chemicals

Animal and environmental toxicity (ecotoxicity; aquatic toxicity) values for each chemical or its related mineral ions were primarily extracted from Material Safety Data Sheets (MSDSs), which compile data from reliable sources such as: OECD (Organization for Economic Co-operation and Development) standard test guideline, the ECOTOX database of the U.S. Environmental Protection Agency (EPA), and the European Chemicals Agency (ECHA; EU REACH database). ECOTOX is a collection of peer-reviewed ecotoxicological test results conducted according to internationally recognized (e.g., OECD or ASTM) test methods. Where the source of reported values was not specified, the values were designated as "unverified data". When multiple values were available from various reliable sources (e.g., ECHA, ECOTOX, OECD-based), the lowest value was selected to ensure a conservative approach. If both reliable and unverified data were available, the value from the reliable source was prioritized, even if the unverified source reported a more conservative figure. Unverified data were used only when no reliable data were available. In addition, published studies reporting relevant standard toxicological tests were also referred to where available. Guideline threshold values were retrieved from reports and official websites of the Environmental Protection Agency (EPA) and the World Health Organization (WHO). The toxicity and ecotoxicity values of the chemicals are presented in Tables S8–S11. In each Table, similar toxicity tests or endpoints were compared across different chemicals for each stage (alkaline treatment, bleaching treatment, and acid hydrolysis), and penalty points were assigned to each chemical according to the relevant toxicity risk. Chemicals with lower toxicity values were assigned greater penalty points, indicating their relatively higher toxicity. If a specific benchmark value was not available for even one chemical within each group, that benchmark was excluded for the total penalty point calculation. Table S14 summarizes all penalty points and the total points assigned to each chemical.

2.2.6. CNC Scoring and Tiering Rubric: A Practical Guide

Scoring Rules

Preparing the Scoring Rubric presented here required some rules and assumptions which are detailed as follows:

- Penalty points related to Labor, Waste, and Cost arising from capital-intensive devices, including high-pressure vessels (autoclaves/steam guns/steam explosion digesters), were considered only when such facilities were directly utilized for any of the main stages of CNC isolation (alkaline and bleaching treatments and acid hydrolysis). Otherwise, they were excluded from the Scoring Rubric to avoid bias in the Tier evaluation. Including such equipment would shift the evaluation toward comparing methodologies in terms of the infrastructure available to researchers rather than the chemicals and experimental conditions involved in the main stages. Furthermore, specific devices such as high-shear homogenizers, spray dryers, freeze dryers, probe-type sonicators, and dialysis membranes (to neutralize CNC suspensions), which are typically utilized after acid hydrolysis to finalize the CNC isolation process, were not considered for additional penalty points, as these practical differences reflect the availability of facilities instead of methodological efficiency.
- Another critical factor was the type of feedstock, which often determines whether pre-treatments such as dewaxing (commonly carried out using a Soxhlet extractor and organic solvents) or kraft pulping (using a high-pressure digester for woody materials) are required. These pre-treatments, related consumables, and experimental conditions were excluded from the penalty-point calculation to avoid feedstock-related bias in selection metrics and because they are not part of the three main stages. Moreover, as these operations vary greatly across

raw biomass, agricultural residues, processed pulp, and commercial cellulose, including them without a separate, standardized feedstock-level analysis could introduce additional bias; therefore, they were excluded.

- If any acids (such as acetic acid) or bases (such as NaOH) were used during the bleaching stage, either for pH adjustment or in combination with the bleaching agent (for example, low-concentration NaOH used with H_2O_2), the related Labor, Cost and Waste penalty points were incorporated into the "pH Adjustment Buffer/Acidic Additive" or "pH Adjustment Basic Additive" sections in the Rubric under the bleaching stage.
- In a few cases across the reviewed papers (approximately 5 cases), an additional experimental stage was conducted between the alkaline and bleaching treatments, typically using organic acids such as formic and acetic acid in combination with H_2O_2. The Labor, Cost, and Waste points associated with this extra stage were reflected in the section "if Specific Chemicals such as acetic acid and formic acid were used together with H_2O_2 as an extra stage" under the bleaching stage in the Rubric. However, if this sort of treatment was performed before starting the main stages (alkaline and bleaching treatments and acid hydrolysis), it was considered as pre-treatment and therefore excluded from the penalty-point calculation to avoid introducing pre-treatment bias in selection metrics.
- The Rubric is designed to consider only one chemical per processing stage when assigning chemical-related penalty points; that is, only one alkaline, one bleaching agent, and one acid are evaluated in the scoring system. Therefore, for exceptional cases in which more than one chemical from the same family (alkaline, bleaching, or acid) was used within a single stage, the conditions below were defined to adapt such cases to the scoring framework:

- There were two unusual cases in the reviewed papers: in one instance, two bleaching agents (for example, $NaClO_2$ and NaClO) were used consecutively, with the latter being applied at a very low concentration at room temperature for an hour. Here, the other bleaching agent ($NaClO_2$), which was used at a higher concentration and under more specific operational conditions (e.g., at 75°C for 5 hours), was considered the primary bleaching treatment. In the second case, chlorinated (NaClO) and the green bleaching agent (H_2O_2) were both used under different specific operational conditions. Here, according to the worst-case scenario rule, the non-green agent and its corresponding operational conditions were considered as those of the bleaching stage. However, the number of bleaching cycles was increased by 1 to account for the bleaching agent/step that was ignored.
- In a few cases (n = 20 of 689), an organic acid (e.g., formic or citric acid) was used for acid hydrolysis in combination with a very low concentration of a mineral acid (e.g., HCl). In such cases, only the organic acid was considered as the chemical for this stage, consistent with its description as the primary acid in the original studies.

Scoring Details: Labor Points

Labor points (expressed as + followed by a number (e.g., +1)) were assigned according to parameters that contribute to the labor-intensiveness of the process, such as time, the number of stage cycles, the presence of additional adjustment steps (e.g., pH and extra stage), and the operation of specific devices such as high-pressure vessels for running the stage. Time-derived labor penalties in each stage were assigned based on chemical-specific time-based operational regimes (low, moderate, high) rather than on absolute treatment duration, to account for intrinsic kinetic variations among chemicals and to facilitate reasonable cross-chemical comparisons. The penalty points increased as the operational

regime progressed from low to high intensity. The detailed criteria for penalty point assignment to calculate the Labor points for each stage are provided below:

Alkali treatment (L_a)

+0.5 if "Alkaline Treatment Time" ≤ 8.8 h (for NaOH) or < 2.75 h (for KOH)

+1 if "Alkaline Treatment Time" = 2.75 – 4.75 h (for KOH)

+1.5 if "Alkaline Treatment Time" > 8.8 h (for NaOH) or > 4.75 h (for KOH)

+0.5 if "Number of Alkaline Treatment Cycle" < 2

+1 if "Number of Alkaline Treatment Cycle" = 2 – 4

+1.5 if "Number of Alkaline Treatment Cycle" > 4

+0.5 if "Alkaline Treatment was performed using high-pressure vessels (autoclaves/steam guns/steam explosion digesters)"

Bleaching treatment (L_b)

+0.5 if "Bleaching Treatment Time" < 1.84 h (for H_2O_2) or < 1.70 h (for $NaClO_2$) or < 1.72 h (for NaClO)

+1 if "Bleaching Treatment Time" 1.84 – 12.92 h (for H_2O_2) or 1.70 – 3.09 h (for $NaClO_2$) or 1.72 – 4.86 h (for NaClO)

+1.5 if "Bleaching Treatment Time" > 12.92 h (for H_2O_2) or > 3.09 h for ($NaClO_2$) or > 4.86 h (for NaClO)

+0.5 if "Number of Bleaching Treatment Cycle" < 3 (for H_2O_2 & $NaClO_2$) or < 2 (for NaClO)

+1 if "Number of Bleaching Treatment Cycle" = 3 – 4 (for H_2O_2) or 3 – 7 (for $NaClO_2$) or 2 – 4 (for NaClO)

+1.5 if "Number of Bleaching Treatment Cycle" > 4 (for H_2O_2 & NaClO) or > 7 (for $NaClO_2$)

+0.5 if "Post Bleach Reducing Rinse" was used (using reducing salts such as $Na_2S_2O_5$, Na_2SO_3, $NaHSO_3$, etc.)

+0.25 if "pH Adjustment Buffer/Acidic Additive" was used (acetic acid, HCl, etc.)

+0.25 if "pH Adjustment Basic Additive" was used (NaOH)

+0.5 if "Specific Chemicals such as acetic acid and formic acid were used together with H_2O_2 as an extra stage"

+0.5 if "Bleaching Treatment was performed using high-pressure vessels (autoclaves/steam guns/steam explosion digesters)"

Acid hydrolysis (L_h)

+0.5 if "Acid Hydrolysis Time" ≤ 156.25 min (for H_2SO_4) or < 150 min (for Citric acid) or < 360 min (for Formic acid) or < 120 min (for H_3PO_4) or < 146.55 min (for HCl) or < 240 min (for Lactic acid)

+1 if "Acid Hydrolysis Time" = 150 – 303.51 (for Citric acid) or 360 – 720 min (for Formic acid) or 120 – 300 min (for H_3PO_4) or 146.55 – 493.42 min (for HCl) or 240 – 300 min (for Lactic acid)

+1.5 if "Acid Hydrolysis Time" > 156.25 (for H_2SO_4) or > 303.51 min (for Citric acid) or > 720 min (for Formic acid) or > 300 min (for H_3PO_4 & Lactic acid) or > 493.42 min (for HCl)

+0.5 if "Acid hydrolysis was performed using high-pressure vessels (autoclaves/steam explosion digesters)"

Scoring Details: Waste Points

Waste points (expressed as + followed by a number (e.g., +1)) were assigned relative to the environmental friendliness of the chemicals as discussed in the "Ranking Chemicals According to Environmental Friendliness" section, increasing from the most environmentally friendly to the least environmentally friendly one. The core parameters contributing to waste were chemical concentration,

experimental temperature, number of stage cycles, and the presence of additional adjustment steps (e.g., pH and extra stage) or high-pressure vessels. Temperature-derived waste penalties in each stage were assigned based on chemical-specific operational temperature regimes (low, moderate, high) rather than absolute temperature values, to account for intrinsic differences in effective treatment conditions among chemicals and facilitate reasonable cross-chemical comparison. In addition to the environmental friendliness of the chemicals within each stage, the literature-derived concentration distribution ranges (operational regimes) for each chemical were also used to assign concentration-derived waste points, which, in all cases, increased as the operational regime progressed from low to high intensity. The detailed criteria for penalty point assignment to calculate the Waste points for each stage are provided below:

Alkali treatment (W_a)

Environmental friendliness order: KOH > NaOH

+0.5 if "KOH concentration" < 0.82 M

+1 if "KOH concentration" = 0.82 – 1.34 M

+1.5 "KOH concentration" > 1.34 – 1.78 M

+2 if "NaOH concentration" < 0.78 M

+2.5 if "NaOH Concentration" = 0.78 – 3.16 M

+3 if "NaOH Concentration" > 3.16 – 6.25 M

+0.5 if "Number of Alkaline Treatment Cycle" < 2

+1 if "Number of Alkaline Treatment Cycle" = 2 – 4

+1.5 if "Number of Alkaline Treatment Cycle" > 4

+0.5 if "Alkaline Treatment Temperature" < 56.56 °C (for KOH) or < 52.36 °C (for NaOH)

+1 if "Alkaline Treatment Temperature" = 56.56 – 90 °C (for KOH) or = 52.36 – 89.17 °C (for NaOH)

+1.5 if "Alkaline Treatment Temperature" > 90 °C (for KOH) or > 89.17 °C (for NaOH)

+0.5 if "Alkaline Treatment was performed using high-pressure vessels (autoclaves/steam guns/steam explosion digesters"

Bleaching treatment (W_b)

Environmental friendliness order: H_2O_2 >> NaClO > $NaClO_2$

+0.5 if "H_2O_2 concentration" < 2.06 M

+1 if "H_2O_2 concentration" = 2.06 – 6.04 M

+1.5 if "H_2O_2 concentration" > 6.04 – 11.66 M

+2 if "NaClO Concentration" < 0.59 M

+2.5 if "NaClO Concentration" = 0.59 – 0.97 M

+3 if "NaClO Concentration" > 0.97 – 4 M

+3.5 if "$NaClO_2$ Concentration" < 0.14 M

+4 if "$NaClO_2$ Concentration" 0.14 – 0.47 M

+4.5 if "$NaClO_2$ Concentration" > 0.47 – 1.4 M

+0.5 if "Number of Bleaching Treatment Cycle" < 3 (for H_2O_2 & $NaClO_2$) or < 2 (for NaClO)

+1 if "Number of Bleaching Treatment Cycle" = 3 – 4 (for H_2O_2) or 3 – 7 (for $NaClO_2$) or 2- 4 (for NaClO)

+**1.5** if "Number of Bleaching Treatment Cycle" > 4 (for H_2O_2 & NaClO) or > 7 (for $NaClO_2$)

+0.5 if "Bleaching Treatment Temperature" < 59.81 °C (for H_2O_2**)** or < 65.69 °C
(for $NaCLO_2$) or < 76.49 °C (for NaCLO)

+1 if "Bleaching Treatment Temperature" = 59.81 – 100 °C (for H_2O_2) or = 65.69 – 88.22 °C (for $NaCLO_2$) or = 76.49 – 97.50 °C (for NaCLO)

+1.5 if "Bleaching Treatment Temperature" > 100 °C (for H_2O_2) or > 88.22 °C (for $NaClO_2$) or > 97.50 °C (for NaCLO)

+0.5 if "Post Bleach Reducing Rinse" was used (using reducing salts such as $Na_2S_2O_5$, Na_2SO_3, $NaHSO_3$, etc)

+0.25 if "pH Adjustment Buffer/Acidic Additive" was used (acetic acid, HCl, etc.)

+0.25 if "pH Adjustment Basic Additive" was used (NaOH)

+0.5 if "if Specific Chemicals such as acetic acid and formic acid were used together with H_2O_2 as an extra stage"

+0.5 if "Bleaching Treatment was performed using high-pressure vessels (autoclaves/steam guns/steam explosion digesters)"

Acid hydrolysis (W_h)

Environmental friendliness order: Lactic acid > Formic acid > Citric acid >> Phosphoric acid = Sulfuric acid > Hydrochloric acid

+0.5 if "Lactic acid Concentration" < 5.50 N

+1 if "Lactic acid Concentration" = 5.50 – 6.50 N

+1.5 if "Lactic acid Concentration" **>** 6.50 N

+2 if "Formic acid Concentration" < 14.7 N

+2.5 if "Formic acid Concentration" = 14.7 – 25.94 N

+3 if "Citric acid Concentration" < 7.69 N

+3.5 if "Citric acid Concentration" = 7.69 – 14.93 N

+4 if "Citric acid Concentration" **>** 14.93 N

+4.5 if "H_3PO_4 Concentration" **<** 26.02 N or if "H_2SO_4 Concentration < 16.1 N

+5 if "H_3PO_4 Concentration" = 26.02 – 27 N or if "H_2SO_4 Concentration" = 16.01 – 19.36 N

+5.5 if "H_3PO_4 Concentration" > 27 – 32.1 N or if "H_2SO_4 Concentration" > 19.36 – 35.93 N

+6 if "HCl Concentration" < 5.42 N

+6.5 if "HCl Concentration" = 5.42 –10.15 N

+7 if "HCl Concentration" > 10.15 – 10.72 N

+0.5 if "Hydrolysis Temperature" < 89.37 °C (for Lactic acid) or < 79.87 °C (for Citric acid) or < 48.59 °C (for H_2SO_4) or < 73.75 °C (for H_3PO_4) or < 73.69 °C (for HCl)

+1 if "Hydrolysis Temperature" = 89.37 – 109.37 °C (for Lactic acid) or = 95 or 100 (for Formic acid) or = 79.87 – 114.72 °C (for Citric acid) or = 48.59 – 70 °C (for H_2SO_4) or = 73.75 – 100 °C (for H_3PO_4) or = 73.69 – 103.57 °C (for HCl)

+1.5 if "Hydrolysis Temperature" > 109.37 °C (for Lactic acid) or > 114.72 °C (for Citric acid) or > 70 °C (for H_2SO_4) or > 100 °C (for H_3PO_4) or > 103.57 °C (for HCl)

+0.5 if "Acid hydrolysis was performed using high-pressure vessels (autoclaves/steam guns/steam explosion digesters)"

Scoring Details: Cost Points

Cost points (expressed as + followed by a number (e.g., +1)) were assigned relative to the final calculated price and corresponding price categories of the chemicals, as explained in the "Ranking Chemicals According to Price" section, increasing from the cheapest to the most expensive one. Concentration-derive cost penalties were assigned using unified (study-wide) price categories according to available sub-categories within each stage (as shown in Table S6), with the penalty points being increased for chemicals by progressing from the lowest subcategory (Low 1, with +0.5 penalty point) to the highest subcategory (Extreme, with +7 penalty points), with +0.5 penalty increment to move between the two adjacent sub-categories to ensure harmonized cross-stage penalty calibration

(Table S6). This standardized penalty scoring was applied consistently to all three chemical classes (alkaline chemicals, bleaching chemicals, and acids) across their respective processing stages (alkaline treatment, bleaching treatment, and acid hydrolysis). When certain sub-categories were missing within a given stage, the pre-defined penalty sequence was maintained and applied to the available sub-categories. For example, if Low 2 was the lowest detected alkaline price category, the initial penalty was set to +1.0, suggesting the absence of the Low 1 sub-category (+0.5), thereby protecting the integrity of the standardized penalty scoring framework. Apart from chemical concentration, experimental temperature, number of experimental cycles, and the presence of additional adjustment steps (e.g., pH and extra stage), and high-pressure vessels were also considered contributors to cost and were consequently assigned corresponding penalty points, similar to their contributions to waste. The detailed criteria for penalty point assignment to calculate the Cost points for each stage are provided below:

Alkali treatment (C_a)

+0.5 if "NaOH Concentration" < 0.78 M (< $ 2.16) or if "KOH Concentration" < 0.82 M (< $ 3.79)

+1 if "NaOH Concentration" = 0.78 – 3.16 M ($ 2.16 – 8.74) or if "KOH Concentration" = 0.82 – 1.34 M ($ 3.79 – 6.19) or if "KOH Concentration" > 1.34 – 1.78 M ($ > 6.19 – 8.22)

+1.5 if "NaOH Concentration" > 3.16 - 6.25 M ($ > 8.74 – 17.28)

+0.5 if "Number of Alkaline Treatment Cycle" < 2

+1 if "Number of Alkaline Treatment Cycle" = 2 – 4

+1.5 if "Number of Alkaline Treatment Cycle" > 4

+0.5 if "Alkaline Treatment Temperature" < 56.56 °C (for KOH) or < 52.36 °C (for NaOH)

+1 if "Alkaline Treatment Temperature" = 56.56 – 90 °C (for KOH) or = 52.36 – 89.17 °C (for NaOH)

+1.5 if "Alkaline Treatment Temperature" > 90 °C (for KOH) or > 89.17 °C (for NaOH)

+0.5 if "Alkaline Treatment was performed using high-pressure vessels (autoclaves/steam guns/steam explosion digesters)"

Bleaching treatment (C_b)

+0.5 if "$NaClO_2$ Concentration" < 0.14 M (< $ 4.32)

+1 if "H_2O_2 Concentration" < 2.06 M ($ < 8.77) or if "$NaClO_2$ Concentration" = 0.14 – 0.47 M ($ 4.32 – 14.49)

+1.5 if "H_2O_2 Concentration" = 2.06 – 6.04 M ($ 8.77 – 25.71)

+2 if "Bleach Concentration" = Medium 1

+2.5 if "H_2O_2 Concentration" > 6.04 – 11.66 M ($ > 25.71 – 49.64) or if "$NaClO_2$ Concentration" > 0.47 – 1.4 M (> $ 14.49 – 43.15)

+3 if "NaClO Concentration" < 0.59 M (< $ 50.72)

+3.5 if "Bleach Concentration" = High 1

+4 if "Bleach Concentration" = High 2

+4.5 if "NaClO Concentration" = 0.59 – 0.97 M ($ 50.72 – 83.39)

+5 if "Bleach Concentration" = Very High 1

+5.5 if "Bleach Concentration" = Very High 2

+6 if "Bleach Concentration" = Very High 3

+6.5 if "NaClO Concentration" > 0.97 – 4 M ($ > 83.39 – 343.89)

+0.5 if "Number of Bleaching Treatment Cycle" < 3 (for H_2O_2 & $NaClO_2$) or < 2 (for NaClO)

+1 if "Number of Bleaching Treatment Cycle" = 3 – 4 (for H_2O_2) or 3 – 7 (for $NaClO_2$) or 2- 4 (for NaClO)

+1.5 if "Number of Bleaching Treatment Cycle" > 4 (for H_2O_2 & NaClO) or > 7 (for $NaClO_2$)

+0.5 if "Bleaching Treatment Temperature" < 59.81 °C (for H_2O_2) or < 65.69 °C (for $NaCLO_2$) or < 76.49 °C (for NaCLO)

+1 if "Bleaching Treatment Temperature" = 59.81 – 100 °C (for H_2O_2) or = 65.69 – 88.22 °C (for $NaCLO_2$) or = 76.49 – 97.50 °C (for NaCLO)

+1.5 if "Bleaching Treatment Temperature" > 100 °C (for H_2O_2) or > 88.22 °C (for $NaCLO_2$) or > 97.50 °C (for NaCLO)

+0.5 if "Post Bleach Reducing Rinse" was used (using reducing salts such as $Na_2S_2O_5$, Na_2SO_3, $NaHSO_3$, etc)

+0.25 if "pH Adjustment Buffer/Acidic Additive" was used (acetic acid, HCl, etc.)

+0.25 if "pH Adjustment Basic Additive" was used (NaOH)

+0.5 if "if Specific Chemicals such as acetic acid and formic acid were used together with H_2O_2 as an extra stage"

+0.5 if "Bleaching Treatment was performed using high-pressure vessels (autoclaves/steam guns/steam explosion digesters)"

Acid hydrolysis (C_h)

+0.5 if "Acid Concentration" = Low 1

+1 if "HCl Concentration" < 5.42 N (< $ 14.55)

+1.5 if “HCl Concentration” = 5.42 – 10.15 N (< $ 14.55 – 27.25) or if “HCl Concentration” > 10.15 – 10.72 N ($ > 27.25 – 28.78) or if “H_2SO_4 Concentration” < 16.01 N ($ < 26.00)

+**2** if “H_2SO_4 Concentration” = 16.01 – 19.36 N ($ 26.00 – 31.44)

+2.5 if “Lactic acid Concentration” < 5.50 N ($ < 46.60) or if “Formic acid Concentration” < 14.7 N ($ < 49.83)

+3 if “H_2SO_4 Concentration” > 19.36 – 35.93 N ($ > 31.44 – 58.35) or if “Citric acid Concentration” < 7.69 N (< $ 59.69) or if “Lactic acid Concentration” = 5.5 – 6.5 N ($ 46.60 – 55.07) or if “Lactic acid Concentration” > 6.5 – 7 N ($ > 55.07 – 59.31)

+3.5 if **“**H_3PO_4 Concentration” < 26.02 N ($ < 69.36)

+4 if **“**H_3PO_4 Concentration” = 26.02 – 27 N ($ 69.36 – 71.97)

+4.5 if **“**H_3PO_4 Concentration” > 27 – 32.1 N ($ > 71.97 – 85.57) or if “Formic acid Concentration” = 14.7 – 25.94 N ($ 49.83 – 87.93)

+5 if **“**Citric acid Concentration” = 7.69 – 14.93 N ($ 59.69 – 115.90)

+5.5 if **“**Citric acid Concentration” > 14.93 – 18 N ($ > 115.90 – 139.73)

+0.5 if “Hydrolysis Temperature” **<** 89.37 °C (for Lactic acid) or **<** 79.87 °C (for Citric acid) or **<** 48.59 °C (for H_2SO_4) or **<** 73.75 °C (for H_3PO_4) or **<** 73.69 °C (for HCl)

+1 if “Hydrolysis Temperature” = 89.37 – 109.37 °C (for Lactic acid) or = 95 or 100 (for Formic acid) or = 79.87 – 114.72 °C (for Citric acid) or = 48.59 – 70 °C (for H_2SO_4) or = 73.75 – 100 °C (for H_3PO_4) or = 73.69 – 103.57 °C (for HCl)

+1.5 if “Hydrolysis Temperature” **>** 109.37 °C (for Lactic acid) or **>** 114.72 °C (for Citric acid) or **>** 70 °C (for H_2SO_4) or **>** 100 °C (for H_3PO_4) or **>** 103.57 °C (for HCl)

+0.5 if "Acid_hydrolysis was performed using high-pressure vessels (autoclaves/steam guns/steam explosion digesters)"

2.2.7. Sensitivity Analysis and Robustness of the Scoring Framework

All sensitivity analyses were performed in R using the *readxl*, *dplyr*, *tidyr*, and *purrr* packages. The analyses included category-weight sensitivity, alternative penalty spacing, Monte Carlo perturbation, and bootstrap evaluation of Tier-boundary stability

Category-weight sensitivity test: a one-at-a-time sensitivity analysis was conducted by independently increasing or decreasing the Labor, Waste, and Cost category weights by 25% relative to the baseline weight of 1.0. For each scenario, stage-level scores, category totals, overall process scores, percentile thresholds, and Tier assignments were recalculated.

Alternative penalty spacing: The sensitivity of the framework to the numerical spacing of the ordinal Low–Moderate–High penalties was evaluated by comparing the original scheme (0.5/1.0/1.5) with a compressed scheme (1.0/1.5/2.0) and an expanded scheme (1.0/2.0/3.0). The transformations were applied to regime-based penalties for time, temperature, and cycles; to concentration penalties; and to both groups simultaneously. Material-specific baseline penalties representing the cross-chemical toxicity or cost ordering were preserved, while only the within-chemical spacing among operational regimes was modified. Binary surcharges were held constant because they did not represent graded operational regimes.

Monte Carlo perturbation: Two Monte Carlo analyses were performed. First, 10,000 simulations were conducted in which the Labor, Waste, and Cost category weights were independently multiplied by random factors drawn from a uniform distribution, U (0.75, 1.25). Second, a more stringent analysis of 5,000 simulations independently perturbed every non-zero component penalty using the same factor range. In every iteration, all scores, P33 and P67 thresholds, and Tier assignments were recalculated.

Bootstrap stability of Tier boundaries: The sampling stability of the percentile thresholds was evaluated using 2,000 non-parametric bootstrap resamples of the process configurations, sampled with replacement. P33 and P67 were recalculated for every metric in each bootstrap replicate, and the bootstrap medians and 95% percentile intervals were obtained. For each scenario, all relevant scores were recalculated, and Tier boundaries were re-estimated from the empirical P33 and P67 percentiles of the modified score distributions. The unperturbed percentile-based classification was used as the baseline reference. Robustness was assessed using the percentage of process configurations retaining their baseline Tier and the Spearman rank correlation between the perturbed and baseline continuous scores.

2.2.8. Illustrative Application of the Rubric

Illustrative application of the proposed CNC isolation Rubric system to classify each of the three processing stages and the entire isolation process from Labor intensiveness, Waste generation, and Cost perspectives, along with identifying the total burden of a specific category across the process, is provided in this section through representative examples of the reviewed studies. The step-by-step procedure for obtaining scores and Tier classifications by following the Rubric system is as follows:

First, chemicals and experimental conditions (operational variables) of the target CNC isolation process are listed and sorted by stage. The parameters includes chemical type, chemical concentration (based on molarity for alkaline and bleaching chemicals and normality for acids), treatment duration (operational time, hours for alkaline and bleaching treatments and minutes for acid hydrolysis), operational temperature (°C), number of the repeating cycle, utilization of high pressure vessels such as steam explosion digesters or autoclave for performing the stage treatment, post bleach rinse using reducing salts, pH adjustments using acidic and/or basic additives during the bleaching stage, and

performing an additional stage between alkaline and bleaching treatments using specific chemicals such as formic and acetic acid with H_2O_2.

Next, using Table 2, Section 1, the corresponding operational regime of chemical concentration and experimental variables (e.g., temperature, time, and number of cycles), along with the corresponding concentration-derived price category (the same row as the concentration within the price category row), is obtained for each stage based on the chemical used for that stage. For instance, if alkaline treatment was performed using 2.5 M NaOH at 90 °C for 1.5 h in a single cycle, the corresponding regimes for concentration, time, temperature, and cycles, and the corresponding price category would be Moderate, Low, High, Low, and L2, respectively.

Then, using Table 2, Section 2 (Scoring Framework), the corresponding penalty points (scores) for Labor, Waste, and Cost for each parameter and stage are determined. For instance, the assigned penalty points for alkaline treatment mentioned above would be as follows: Labor: +0.5 for time, +0.5 for cycle; Waste: +2.5 for concentration, +0.5 for cycle, +1.5 for temperature; and Cost: +1 for concentration, +0.5 for cycle, and +1.5 for temperature.

After that, the sum of the penalty scores for Labor, Waste, and Cost for each stage is computed to obtain Category-level Total Scores ($L_{total} = L_a + L_b + L_h$, $W_{total} = W_a + W_b + W_h$, and $C_{total} = C_a + C_b + C_h$). Similarly, the Stage-level Total Scores are calculated by summing the scores from three categories ($TP_a = L_a + W_a + C_a$, $TP_b = L_b + W_b + C_b$, and $TP_h = L_h + W_h + C_h$) in each stage. The cumulative score for the entire CNC isolation process (Overall-Process Total Scores, TP_{total}) can be calculated either as the Category-level Total Scores ($TP_{total} = L_{total} + W_{total} + C_{total}$) or as the sum of the Stage-level Total Scores ($TP_{total} = TP_a + TP_b + TP_h$).

Eventually, referring to Table 2, Section 3 (Tiering framework), the Category-level Total Tier, the Stage-level Total Tiers, and the Overall Process Total Tier are obtained by mapping the Category-

level Total Scores (L_{total}, W_{total}, and C_{total}), the Stage-level Total Scores (TP_a, TP_b, and TP_h), and the Overall Process Total Scores (TP_{total}), respectively, onto the specified percentile ranges.

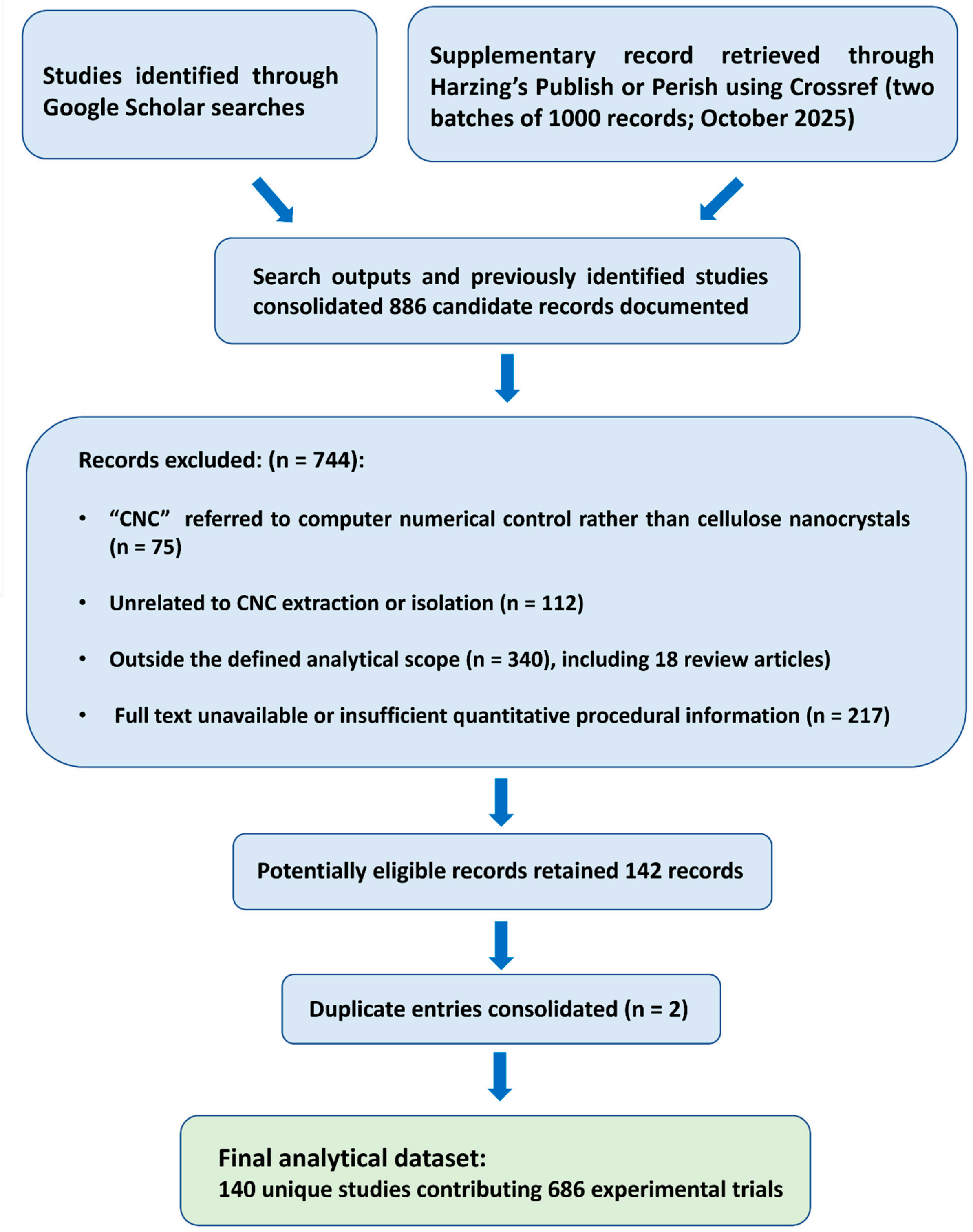

Figure S1. Literature identification and screening flow used to construct the analytical dataset.

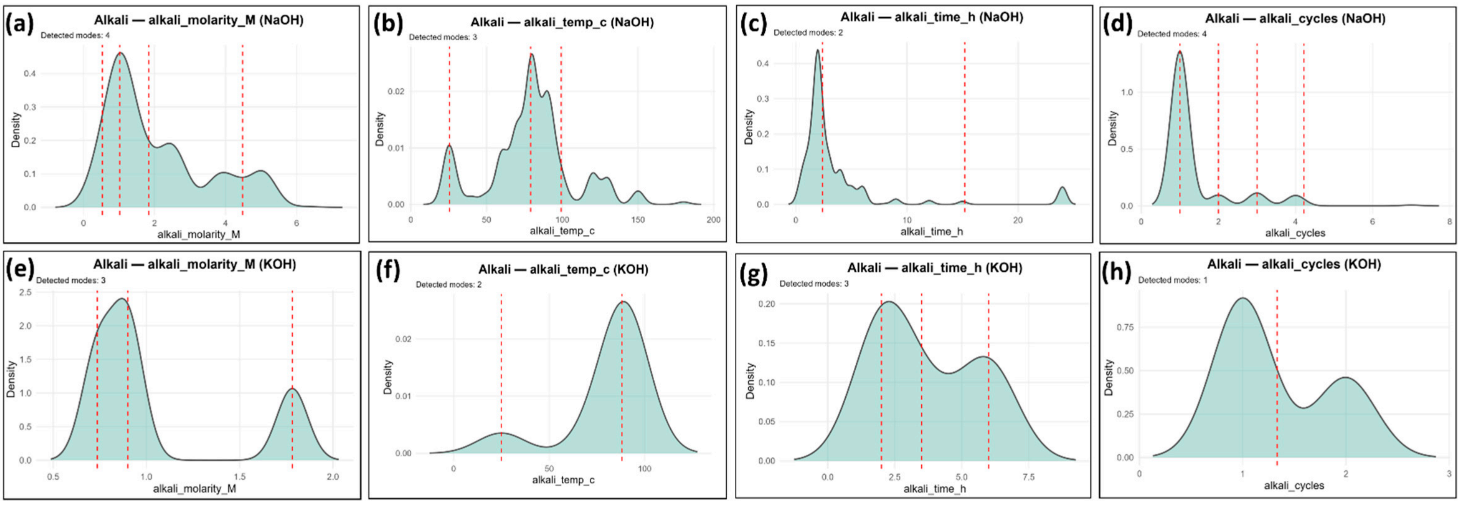

Figure S2. Literature-derived Gaussian Mixture Model (GMM) outputs for experimental parameters of alkaline treatments used during the CNC isolation process. **a–d)** NaOH treatments. **e–h)** KOH treatments. **a & e)** alkaline concentration (molarity). **b & f)** operational temperature (°C). **c & g)** reaction time (h). **d & h)** number of cycles.

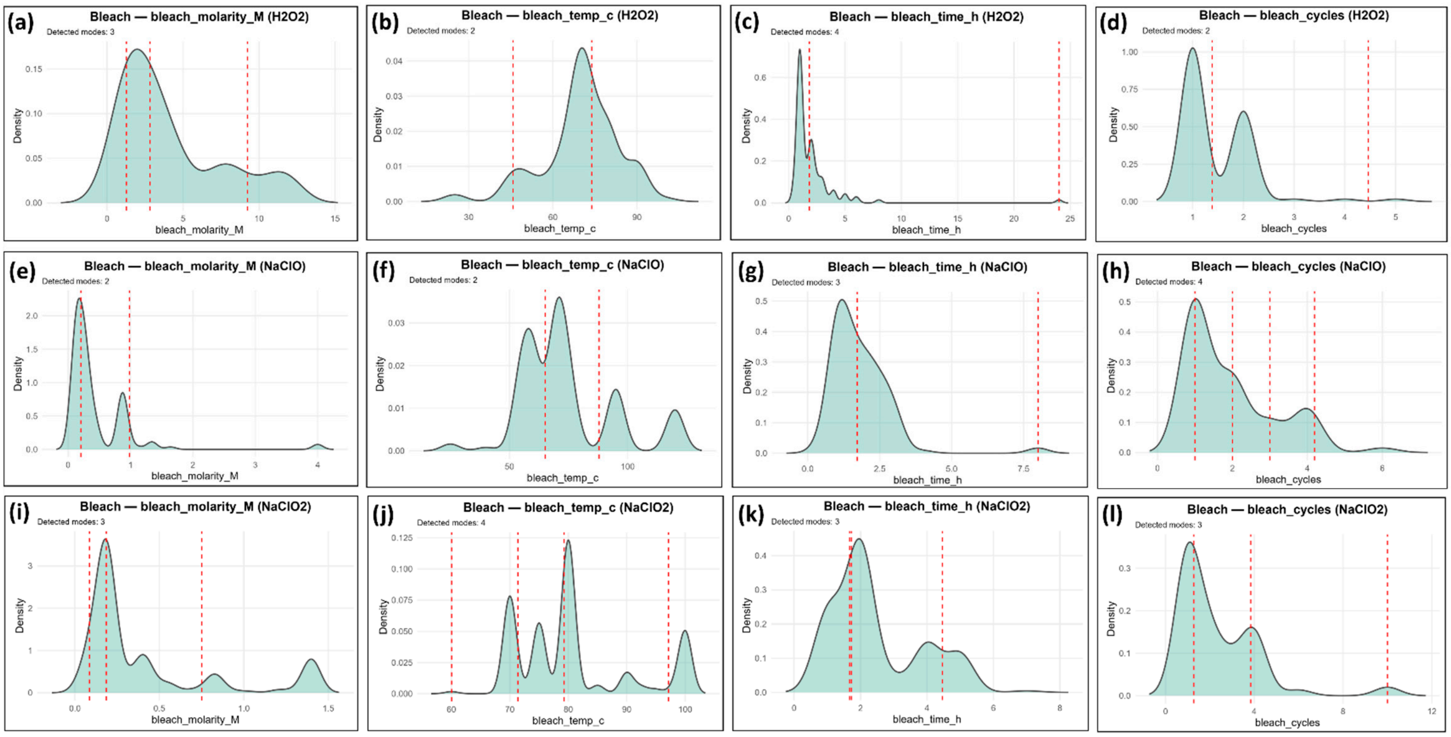

Figure S3. Literature-derived Gaussian Mixture Model (GMM) outputs for experimental parameters of the bleaching treatments used during the CNC isolation process. **a–d)** H_2O_2 treatments. **e–h)** NaClO treatments. **i–l)** $NaClO_2$ treatments. **a**, **e**, & **i)** bleach concentration (molarity). **b**, **f**, & **j)** operational temperature (°C). **c**, **g**, & **k)** reaction time (h). **d**, **h**, & **l)** number of cycles.

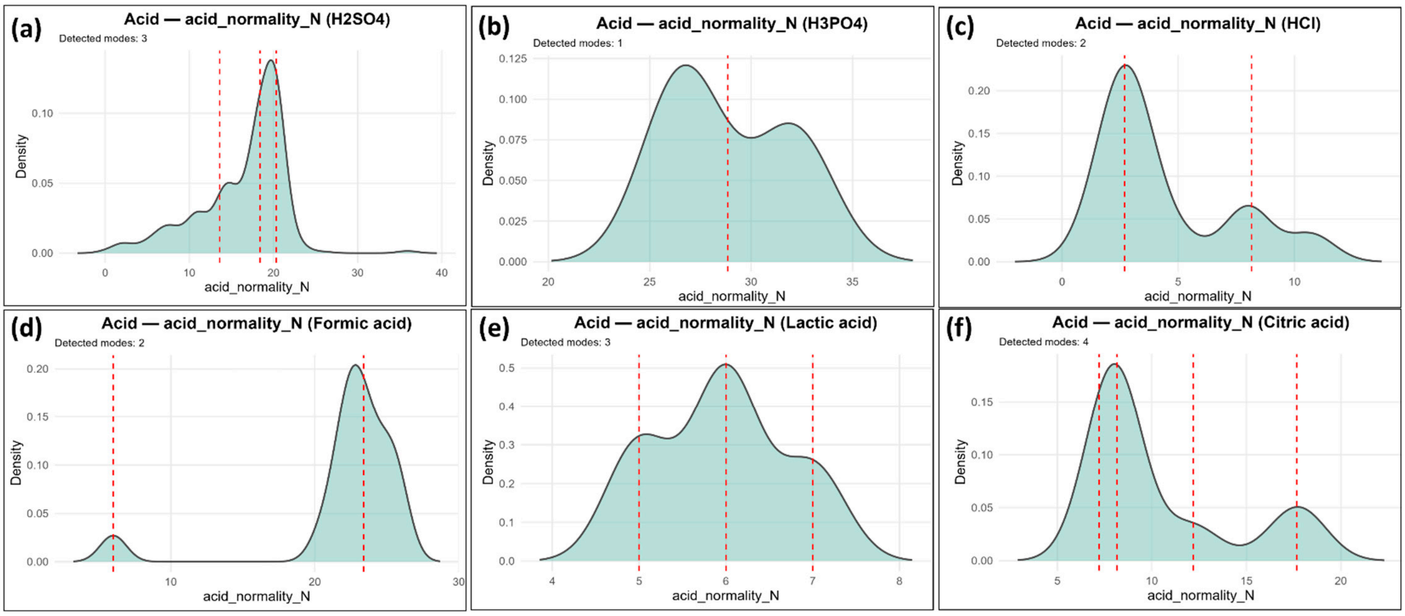

Figure S4. Literature-derived Gaussian Mixture Model (GMM) outputs for acid concentration (expressed as normality) used in acid hydrolysis of the CNC isolation process. **a)** Sulfuric acid. **b)** Phosphoric acid. **c)** Hydrochloric acid. **d)** Formic acid. **e)** Lactic acid **f)** Citric acid.

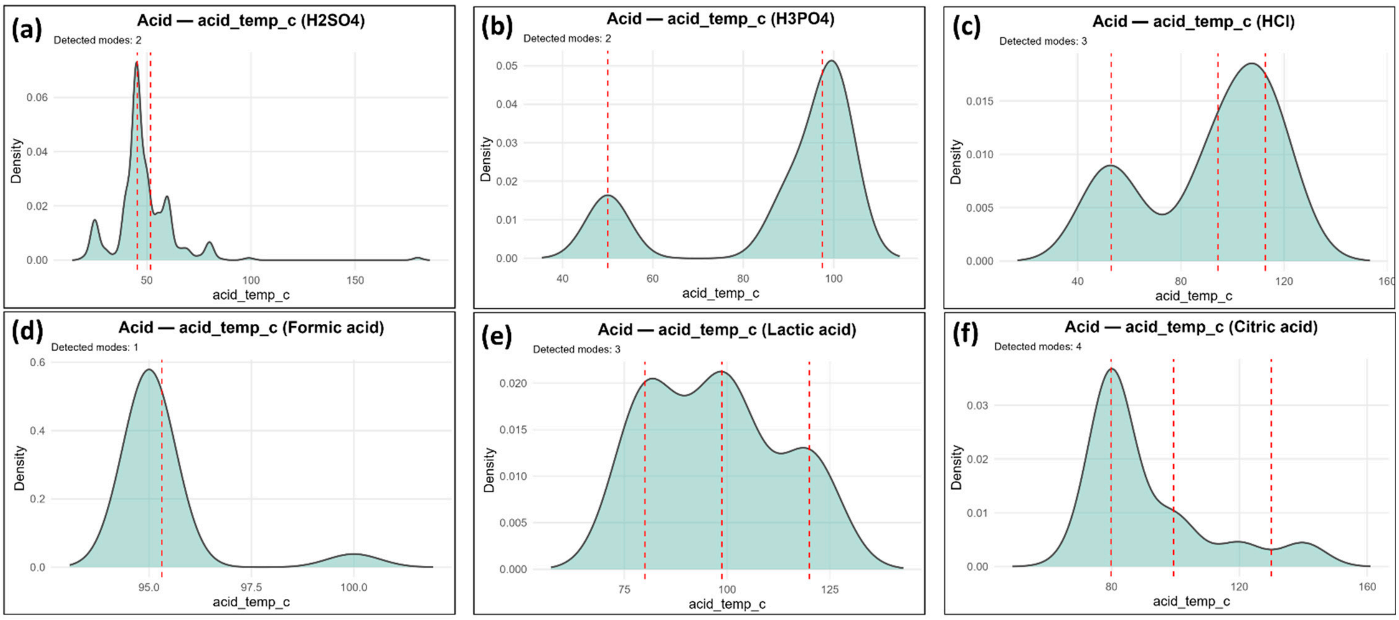

Figure S5. Literature-derived Gaussian Mixture Model (GMM) outputs for operational temperature (°C) used in acid hydrolysis of the CNC isolation process. **a**) Sulfuric acid. **b**) Phosphoric acid. **c)** Hydrochloric acid. **d**) Formic acid. **e**) Lactic acid. **f**) Citric acid.

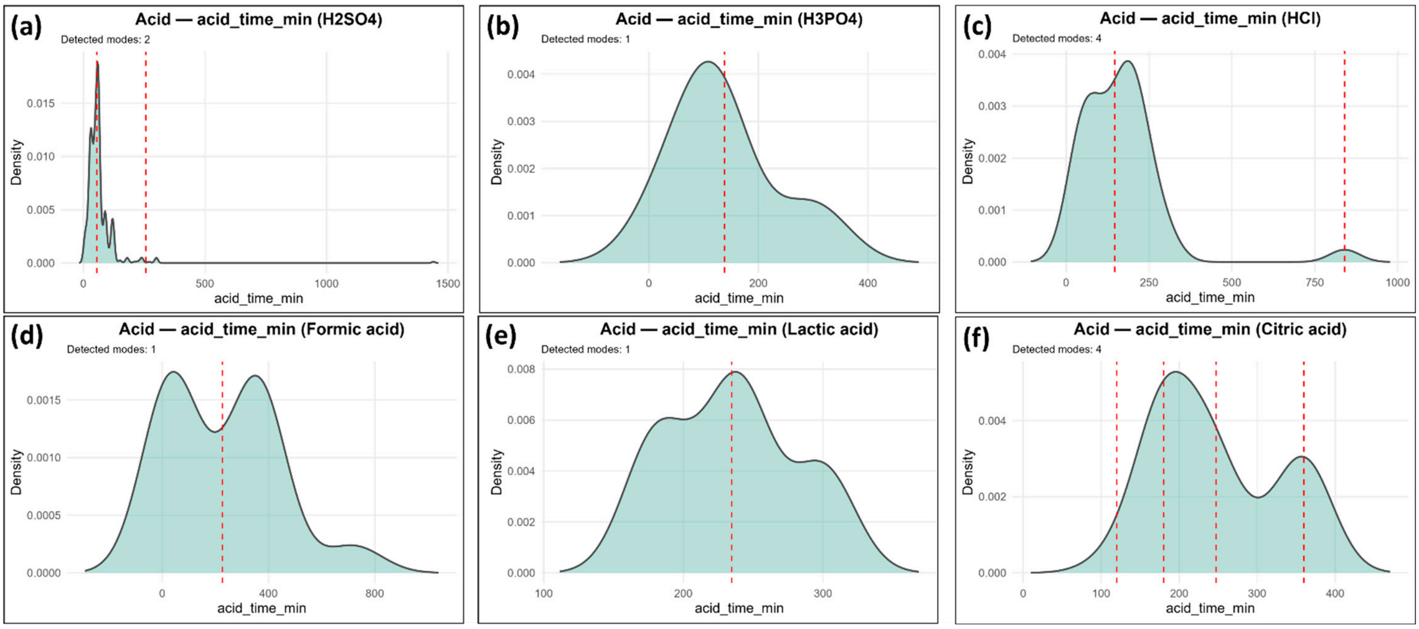

Figure S6. Literature-derived Gaussian Mixture Model (GMM) outputs for reaction time (min) used in acid hydrolysis of the CNC isolation process. **a)** Sulfuric acid. **b)** Phosphoric acid. **c)** Hydrochloric acid. **d)** Formic acid. **e)** Lactic acid. **f)** Citric acid.

Table S1. List of studies included in the current review (*n* = 140).

No	First Author	Study Title	DOI
1	[10]	Insight into the extraction and characterization of cellulose nanocrystals from date pits	https://doi.org/10.1016/j.arabjc.2021.103650
2	[11]	Banana peel waste: an emerging cellulosic material to extract nanocrystalline cellulose	https://doi.org/10.1021/acsomega.2c06571
3	[12]	Production of nanocellulose crystal derived from Enset fiber using acid hydrolysis coupled with ultrasonication, isolation, statistical modeling, optimization, and characterizations	https://doi.org/10.1155/2021/7492532
4	[13]	Isolation of high crystalline nanocellulose from Mimosa pudica plant fibres with potential in packaging applications	https://doi.org/10.1002/pts.2617
5	[14]	Starch bio-nanocomposites based on phosphorylated and sulphated cellulose nanocrystals extracted from pepper plant residue: effect of surface functionality on property improvements	https://doi.org/10.1007/s10570-023-05199-4
6	[15]	Tomato plant residue as new renewable source for cellulose production: extraction of cellulose nanocrystals with different surface functionalities	https://doi.org/10.1007/s10570-020-03097-7
7	[16]	Development of polyvinyl alcohol/chitosan bio-nanocomposite films reinforced with cellulose nanocrystals isolated from rice straw	https://doi.org/10.1016/j.apsusc.2018.01.022
8	[17]	Extraction of cellulose nanocrystals from areca waste and its application in eco-friendly biocomposite film	https://doi.org/10.1016/j.chemosphere.2021.132084
9	[18]	Isolation and Characterization of Cellulose Nanocrystals from Date Palm Waste	https://doi.org/10.1021/acsomega.2c02333
10	[19]	Characteristics of sulfated and carboxylated cellulose nanocrystals extracted from Juncus plant stems	https://doi.org/10.1016/j.ijbiomac.2019.11.023
11	[20]	Isolation and characterization of nanocrystalline cellulose from cocoa pod husk (CPH) biomass wastes	https://doi.org/10.1016/j.heliyon.2021.e06680
12	[21]	Valorization of Eichhornia crassipes for the production of cellulose nanocrystals further investigation of plethoric biobased resource	https://doi.org/10.1038/s41598-024-62159-z
13	[22]	Facile extraction and characterization of cellulose nanocrystals from agricultural waste sugarcane straw	https://doi.org/10.1002/jsfa.11360
14	[23]	Extraction and characterization of cellulose nanocrystals from tea leaf waste fibers	https://doi.org/10.3390/polym9110588
15	[24]	Property investigation of nanocellulose I and II from different non-wood fibers using chemical acid hydrolysis method	https://doi.org/10.1016/j.indcrop.2024.119534
16	[25]	Production of nanocellulose from pineapple leaf fibers via high-Shear homogenization and ultrasonication	https://doi.org/10.3390/fib6020028
17	[26]	Processing of PLA nanocomposites with cellulose nanocrystals extracted from Posidonia oceanica waste: Innovative reuse of coastal plant	http://dx.doi.org/10.1016/j.indcrop.2015.01.075
18	[27]	Extraction of cellulose nanocrystals from red banana peduncle agro-waste and application in environmentally friendly biocomposite film	http://dx.doi.org/10.1002/pc.26755
19	[28]	Exploring cellulose nanocrystals obtained from olive tree wastes as sustainable crop protection tool against bacterial diseases	https://doi.org/10.1038/s41598-022-10225-9
20	[29]	Isolation of cellulose nanocrystals from different waste bio-mass collating their liquid crystal ordering with morphological exploration	https://doi.org/10.1016/j.ijbiomac.2021.02.038

No	First Author	Study Title	DOI
21	[30]	Production of CNC from agro-waste biomass (maize shells) as a potential reinforcement in bio-nanocomposites: Extraction, modification, and characterization study	https://doi.org/10.1016/j.carpta.2025.100671
22	[31]	Production of cellulose nanocrystals from the waste banana (M. oranta) tree rachis fiber as a reinforcement to fabricate useful bionanocomposite	https://doi.org/10.1016/j.carpta.2024.100607
23	[32]	Cellulose nanocrystal (CNC) from okra plant (Abelmoschus esculentus L.) stalks as a reinforcement in bionanocomposite fabrication: Extraction, processing, and characterization study	https://doi.org/10.1016/j.carpta.2024.100581
24	[33]	Isolation and characterization of cellulose nanocrystals from different lignocellulosic residues: A comparative study	https://doi.org/10.1007/s10924-021-02089-3
25	[34]	Optimization of homogenization-sonication technique for the production of cellulose nanocrystals from cotton linter	https://doi.org/10.1016/j.ijbiomac.2019.06.241
26	[35]	Isolation and characterization of cellulose nanocrystals from pineapple crown waste and their potential uses	https://doi.org/10.1016/j.ijbiomac.2018.10.187
27	[36]	Nanolignocellulose extracted from environmentally undesired *Prosopis juliflora*	https://doi.org/10.1021/acsomega.8b02685
28	[37]	Extraction of high crystalline nanocellulose from biorenewable Sources of vietnamese agricultural wastes	https://doi.org/10.1007/s10924-020-01695-x
29	[38]	Cellulose nanocrystal isolation from tomato peels and assembled nanofibers	http://dx.doi.org/10.1016/j.carbpol.2014.12.064
30	[39]	Valorization of khat (Catha edulis) waste for the production of cellulose fibers and nanocrystals	https://doi.org/10.1371/journal.pone.0246794
31	[40]	Highly-efficient isolation of microcrystalline cellulose and nanocellulose from sunflower seed waste via environmentally benign method	https://doi.org/10.1007/s10570-022-04527-4
32	[41]	Isolation of mixed compositions of cellulose nanocrystals, microcrystalline cellulose, and lignin nanoparticles from wood pulps	https://doi.org/10.1021/acsomega.3c00295
33	[42]	Optimization of cellulose nanocrystal isolation from Ayous sawdust using response surface methodology	https://doi.org/10.4236/msce.2024.121004
34	[43]	Walnut shells as cellulose nanocrystal source: isolation procedure and properties characterization	https://doi.org/10.1007/s13399-023-03996-2
35	[44]	Extraction of nanocellulose from pineapple leaves by acid-hydrolysis and pressurized acid hydrolysis for reinforcement in natural rubber composites	https://doi.org/10.1088/1757-899X/526/1/012019
36	[45]	Tailoring the yield and characteristics of wood cellulose nanocrystals (CNC) using concentrated acid hydrolysis	https://doi.org/10.1007/s10570-015-0615-1
37	[46]	Isolation and characterization of CNC from waste maize cob available in Bangladesh as a potential candidate for the fabrication of multifunctional bio-nanocomposites: A new approach	https://doi.org/10.1016/j.sajce.2024.12.007
38	[47]	High yield production of nanocrystalline cellulose from corn cob through a chemical-mechanical treatment under mild conditions	https://doi.org/10.1016/j.ijbiomac.2023.124327
39	[48]	Acid-hydrolysis-assisted cellulose nanocrystal isolation from *Acacia mearnsii* de wild. wood kraft pulp	https://doi.org/10.3390/polym16233371
40	[49]	Evaluation of steam explosion pretreatment on the cellulose nanocrystals (CNCs) yield from poplar wood	https://doi.org/10.1016/j.carbpol.2023.121460

No	First Author	Study Title	DOI
41	[50]	Maximizing production of cellulose nanocrystals and nanofibers from pre-extracted loblolly pine kraft pulp: a response surface approach	https://doi.org/10.1186/s40643-020-00302-0
42	[51]	Impact of species-based wood feedstock variability on physicochemical properties of cellulose nanocrystals	https:// doi.org/ 10. 1007/ s10570- 022-04762-9
43	[52]	Cellulose nanocrystal isolation from hardwood pulp using various hydrolysis conditions	https:// doi.org/ 10.3390/molecules24203724
44	[53]	Isolation, characterization, and comparison of nanocrystalline cellulose from solid wastes of horse chestnut and chestnut seed shell	https://doi.org/10.1007/s10570-022-04682-8
45	[54]	Production of cellulose nanocrystals sSuspension with high yields from water hyacinth	https://doi.org/10.1080/15440478.2022.2134266
46	[55]	Preparation and characterization of cellulose nanocrystal extracted from *Calotropis procera* biomass	https://doi.org/10.1186/s40643-019-0279-z
47	[56]	Isolation and characterization of cellulose nanocrystals from Ensete ventricosum pseudo-stem fiber using acid hydrolysis	https://doi.org/10.1007/s13399-022-02987-z
48	[57]	Chitosan/nanocrystalline cellulose biocomposites based on date palm (*Phoenix Dactylifera* L.) sheath fibers	https://doi.org/10.32604/jrm.2019.00034
49	[58]	Preparation and characterization nanocrystalline cellulose as a food additive to produce healthy biscuit cream	https://doi.org/10.1002/star.202000033
50	[59]	Cellulose isolation from oil palm empty fruit bunch using different pretreatment processes	http://dx.doi.org/10.6180/jase.202311_26(11).0001
51	[60]	Extraction of nanocrystalline cellulose from oil palm empty fruit bunches using sulfuric acid hydrolysis method	http://dx.doi.org/10.1088/1755-1315/1460/1/012022
52	[61]	Optimization of extraction conditions for cellulose from jackfruit peel using RSM, its characterization and comparative studies to commercial cellulose	https://doi.org/10.1016/j.meafoo.2023.100130
53	[62]	Effect of alkaline pretreatment on properties of lignocellulosic oil palm waste	https://doi.org/10.1016/j.proche.2015.12.036
54	[63]	Optimization of sulfuric acid hydrolysis conditions for preparation of nanocrystalline cellulose from enzymatically pretreated fibers	https://doi.org/10.1007/s10570-016-0897-y
55	[64]	Cellulose nanocrystals from natural fiber of the macrophyte Typha domingensis: extraction and characterization	https://doi.org/10.1007/s10570-014-0533-7
56	[65]	Facile one-step extraction and oxidative carboxylation of cellulose nanocrystals through hydrothermal reaction by using mixed inorganic acids	https://doi.org/10.1007/s10570-017-1339-1
57	[66]	Extraction and characterization of cellulose nanocrystals from post-consumer wood fiberboard waste	https://doi.org/10.1007/s10570-017-1252-7
58	[67]	Enhancing mechanical properties of polyvinyl alcohol films through cellulose nanocrystals derived from corncob	https://doi.org/10.1007/s13399-024-06128-6
59	[68]	Sustainable preparation and characterization of thermally stable and functional cellulose nanocrystals and nanofibrils via formic acid hydrolysis	https://doi.org/10.21967/jbb.v2i1.68

No	First Author	Study Title	DOI
60	[69]	Preparation and characterization of thermally stable cellulose nanocrystals via a sustainable approach of FeCl3-catalyzed formic acid hydrolysis	https://doi.org/10.1007/s10570-016-0963-5
61	[70]	Cellulose nanocrystals prepared via formic acid hydrolysis followed by TEMPO-mediated oxidation	http://dx.doi.org/10.1016/j.carbpol.2015.07.033
62	[71]	Nanocrystalline cellulose extracted from pine wood and corncob	http://dx.doi.org/10.1016/j.carbpol.2016.11.036
63	[72]	Optimized isolation and characterization of cellulose for extraction of cellulose nanocrystals from *Ensete ventricosum* pseudo-stem fibre using a two-stage extraction method	https://doi.org/10.1080/17458080.2023.2199989
64	[73]	Extraction and characterization of nanocrystalline cellulose from doum (*Chamaerops humilis*) leaves: a potential reinforcing biomaterial	https://doi.org/10.1007/s10924-016-0784-5
65	[74]	Isolation and characterization of nanocrystalline cellulose isolated from pineapple crown leaf fiber agricultural wastes using acid hydrolysis	https://doi.org/10.3390/polym13234188
66	[75]	Isolation of thermally stable cellulose nanocrystals from spent coffee grounds via phosphoric acid hydrolysis	https://doi.org/10.32604/jrm.2020.07940
67	[76]	Extraction and characterization of cellulose and microcrystalline cellulose from teff straw and evaluation of the microcrystalline cellulose as tablet excipient	https://doi.org/10.1080/15440478.2023.2245565
68	[77]	Cellulose nanocrystal-based films produced by more sustainable extraction protocols from *Posidonia oceanica* waste biomass	https://doi.org/10.1007/s10570-019-02641-4
69	[78]	Isolation of nanocrystalline cellulose from tunicates	https://doi.org/10.1016/j.jece.2018.06.056
70	[79]	Extraction and preparation of cellulose nanocrystals from dealginate kelp residue: structures and morphological characterization	https:// doi.org/10.1007/s10570-015-0617-z
71	[80]	Nanocrystalline cellulose from microcrystalline cellulose of date palm fibers as a promising candidate for bio-nanocomposites: isolation and characterization	https://doi.org/10.3390/ma14185313
72	[81]	Physical and bio-composite properties of nanocrystalline cellulose from wood, cotton linters, cattail, and red algae	https://doi.org/10.1007/s10570-015-0633-z
73	[82]	Optimization and characterization of cellulose extraction from olive pomace	https://doi.org/10.1007/s10570-023-05195-8
74	[83]	Isolation and characterization of nanocrystalline cellulose from sugar palm fibres (*Arenga Pinnata*)	https://doi.org/10.1016/j.carbpol.2017.11.045
75	[84]	Optimization of production process of nanocrystalline cellulose and its composites	https://doi.org/10.1134/S106816202207007X
76	[85]	Optimization and modelling of delignification process for nanocrystalline cellulose production from rice husk biomass	https://doi.org/10.1063/1.5016692
77	[86]	An investigation between high and low pressure processes for nanocrystalline cellulose production from agro-waste biomass	https://doi.org/10.1063/1.5016729
78	[87]	Extraction and characterization of cellulose nanocrystals (CNCs) from jute fibers and other by-products towards a circular economy approach	https://doi.org/10.1007/s42824-024-00149-2
79	[88]	Extraction and characterization of nanocellulose crystals from cotton gin motes and cotton gin waste	https://doi.org/10.1007/s10570-019-02533-7

No	First Author	Study Title	DOI
80	[89]	Sugarcane trash-derived nanocrystalline cellulose: a sustainable approach with mild chemical pretreatment and comprehensive characterization	https://doi.org/10.1007/s10570-024-05906-9
81	[90]	Preparation of nanocrystalliin cellulose from tomato stem and commercial microcrystalline cellulose: A comparison between two starting materials	https://doi.org/10.35812/CelluloseChemTechnol.2019.53.97
82	[91]	Extraction optimization and characterization of cellulose nanocrystals from apricot pomace	https://doi.org/10.3390/foods12040746
83	[92]	Isolation and production of nanocrystalline cellulose from conocarpus fiber	https://doi.org/10.3390/polym13111835
84	[93]	Isolation and characterization of nanocrystalline cellulose from roselle-derived microcrystalline cellulose	https://doi.org/10.1016/j.ijbiomac.2018.03.065
85	[94]	Properties and characteristics of nanocrystalline cellulose isolated from olive fiber	https://doi.org/10.1016/j.carbpol.2020.116423
86	[95]	Nanocrystalline cellulose derived from spruce wood: Influence of process parameters	https://doi.org/10.1016/j.ijbiomac.2022.09.017
87	[96]	Isolation and characterization of nanocrystalline cellulose from different precursor materials	https://doi.org/10.1007/s12221-017-6548-6
88	[97]	Extraction of cellulose nanocrystalline from Camellia oleifera Abel waste shell: Study of critical processes, properties and enhanced emulsion performance	https://doi.org/10.1016/j.ijbiomac.2023.127890
89	[98]	Simultaneous extraction of carboxylated cellulose nanocrystals and nanofibrils via citric acid hydrolysis—a sustainable route	https://doi.org/10.26599/PBM.2017.9260024
90	[99]	Extraction, isolation and characterization of nanocrystalline cellulose from industrial kelp (Laminaria japonica) waste	http://dx.doi.org/10.1016/j.carbpol.2017.05.079
91	[100]	Modulation of acid hydrolysis reaction time for the extraction of cellulose nanocrystals from *Posidonia oceanica* leaves	http://dx.doi.org/10.7569/JRM.2015.634134
92	[101]	Extraction, preparation and characterization of nanocrystalline cellulose from lignocellulosic Simpor leaf residue	https://doi.org/10.3390/molecules30071622
93	[102]	Nanocrystalline cellulose from Calophyllum inophyllum shells waste by adjusting organic acid hydrolysis and optimization of reaction parameters using response surface methodology	https://doi.org/10.1016/j.ijbiomac.2024.135705
94	[103]	Optimum alkaline treatment parameters for the extraction of cellulose and production of cellulose nanocrystals from apple pomace	https://doi.org/10.1016/j.carbpol.2019.03.103
95	[104]	Isolation and characterization of nanocrystalline cellulose From corn husk	http://dx.doi.org/10.1016/j.matlet.2015.02.047
96	[105]	New excipient for oral drug delivery: CNC derived from sugarcane bagasse-derived microcrystalline cellulose	https://doi.org/10.1021/acsomega.4c00497
97	[106]	Acid hydrolysis-mediated preparation of nanocrystalline cellulose from rce straw	http://doi.org/10.17352/2455-3492.000021
98	[107]	Nanocrystalline cellulose of African beans (*Pentaclethra macrophylla*) benth pod: extraction and characterization	https://doi.org/10.46602/jcsn.v48i4.905
99	[108]	Optimization of the product of nanocrystalline cellulose from coconut husks	https://doi.org/10.1088/1757-899X/429/1/012041
100	[109]	Synthesis of barium hexaferrite templated by nanocrystalline cellulose extracted from luffa acutangula fiber	https://doi.org/10.1088/2053-1591/abe5fe

No	First Author	Study Title	DOI
101	[110]	Extraction and characterization of nanocrystalline cellulose from agricultural residue-oil palm petiole fiber	https://doi.org/10.29294/IJASE.9.2.2022.2751-2757
102	[111]	Preparation and characterization of high yield cellulose nanocrystals (CNC) derived from ball mill pretreatment and maleic acid hydrolysis	https://doi.org/10.1016/j.carbpol.2020.115942
103	[112]	Synthesis of cellulose nanocrystals (CNCs) from cotton using ultrasound assisted acid hydrolysis	https://doi.org/10.1016/j.ultsonch.2020.105353
104	[113]	Production of cellulose nanocrystal (CNC) from papaya tree stem as a potential reinforcement in bionanocomposite manufacturing	https://doi.org/10.1016/j.biteb.2025.102284
105	[114]	Production of cellulose nanocrystals from the residual mass of P. chaba stem after methanol extraction as potential reinforcement in sustainable nanocomposite manufacturing: A cutting-edge approach in waste management	https://doi.org/10.1016/j.biteb.2025.102273
106	[115]	Extraction and characterization of cellulose and cellulose nanowhiskers from almond shell biomass, metal removal and toxicity analysis	https://doi.org/10.33263/BRIAC122.17051720
107	[116]	Extraction and characterization of cellulose from agricultural residue-oil palm fronds	https://doi.org/10.17576/mjas-2017-2105-08
108	[117]	Extraction, isolation, and characterization of nanocrystalline cellulose from Barangan banana (*Musa acuminata* L.) peduncles waste	https://doi.org/10.22146/ijc.74718
109	[118]	Characterization of cellulose nanocrystalline isolated from banana peduncles using acid hydrolysis	https://doi.org/10.1088/1755-1315/922/1/012072
110	[119]	Extraction and characterization of nanocrystalline cellulose from sugar palm fibres (*Arenga Pinnata*)	https://doi.org/10.33394/j-ps.v11i2.7935
111	[120]	Extraction of high-quality grade cellulose and cellulose nanocrystals from different lignocellulosic agri-food wastes	https://doi.org/10.3389/fsufs.2022.1087867
112	[121]	The optimization of cellulose content in tobacco stems (*Nicotiana tabacum* L.) with acid extraction method and alkaline extraction method	https://doi.org/10.46676/ij-fanres.v2i2.28
113	[122]	Comparison of extraction methods and characterization of cellulose from sugarcane bagasse (*Saccharum officinarum* L.) for bioplastic food packaging	https://doi.org/10.26538/tjnpr/v8i12.42
114	[123]	Extraction and analysis of cellulose nanocrystals from cotton balls by acid hydrolysis	http://doi.org/10.4038/josuk.v16i1.8075
115	[124]	Comparative analysis of nanocellulose extraction from Cocos nucifera shell by ultrasonication versus acid hydrolysis for methylene blue dye removal	https://doi.org/10.1007/s13726-025-01462-6
116	[125]	Preparation and characterization of cellulose nanocrystal extracted from ramie fibers by sulfuric acid hydrolysis	https://doi.org/10.1016/j.heliyon.2020.e05486
117	[126]	Preparation and characterizations of nanocellulose from wood pulp using hydrolysis method	https://jst.vn/index.php/old/article/view/649
118	[127]	Extraction and characterization of nanocellulose from cattail leaves: Morphological, microstructural and thermal properties	https://doi.org/10.1016/j.ijbiomac.2023.128123
119	[128]	New approach for single-step extraction of carboxylated cellulose nanocrystals for their use as adsorbents and flocculants	https://doi.org/10.1021/acssuschemeng.6b00126

No	First Author	Study Title	DOI
120	[129]	Effect of acid hydrolysis conditions on the extraction of cellulose nanocrystals	https://doi.org/10.3390/polym17101313
121	[130]	Extraction and characterization of cellulose nanocrystals from brown seaweed *Dictyota Bartayresiana*, J.V. *Lamouroux*	https://doi.org/10.1007/s10876-024-02721-x
122	[131]	Sustainable synthesis and characterization of Enset cellulose nanocrystals (E-CNp) from Enset ventricosum biomass and its application in the fabrication of Enset cellulose nanocomposite (E-CNc)	https://doi.org/10.1007/s13399-022-02682-z
123	[132]	Extraction and characterization of nanocrystalline cellulose from cassava bagasse	https://doi.org/10.1007/s10924-017-0983-8
124	[133]	Conversion of sugarcane trash to nanocrystalline cellulose and its life cycle assessment	https://doi.org/10.3390/catal12101215
125	[134]	Isolation and surface modification of cellulose nanocrystals from sugarcane bagasse waste: From a micro- to a nano-scale view	https://doi.org/10.1016/j.apsusc.2017.12.137
126	[135]	Optimization of cellulose nanocrystals extraction from *teff* straw using acid hydrolysis followed by ultrasound sonication	https://doi.org/10.1016/j.carpta.2025.100707
127	[136]	Sustainable extraction of cellulose nanocrystals from empty palm oil bunches via low-acid hydrolysis	https://doi.org/10.1016/j.rineng.2024.103012
128	[137]	Preparation and characterization of cellulose nanocrystal extraction from *Pennisetum hydridum* fertilized by municipal sewage sludge via sulfuric acid hydrolysis	https://doi.org/10.3389/fenrg.2021.774783
129	[138]	Development of nanocomposite films with durian peel nanocrystalline cellulose	10.22487/j24775185.2021.v10.i4.pp230-236
130	[139]	Extraction and comparison of cellulose nanocrystals from lemon (*Citrus limon*) seeds using sulfuric acid hydrolysis and oxidation methods	https://doi.org/10.1016/j.carbpol.2020.116180
131	[140]	Further characterization of cellulose nanocrystal (CNC) preparation from sulfuric acid hydrolysis of cotton fibers	https://doi.org/10.1007/s10570-015-0803-z
132	[141]	Carboxylated nanocrystalline cellulose from *Salix psammophila* prepared by sulfuric acid hydrolysis combined with 2,2,6,6-tetramethylpiperidine-1-oxyl (TEMPO)-oxidation	https://doi.org/10.15376/biores.17.1.1373-1384
133	[142]	Easy way to prepare dispersible CNC dry powder by precipitation and conventional evaporation	https://doi.org/10.1007/s10570-021-04123-y
134	[143]	Effect of hydrochloric acid hydrolysis under sonication-hydrothermal process to produce cellulose nanocrystal from OPEFB	https://doi.org/10.21203/rs.3.rs-3062011/v1
135	[144]	optimization of the obtaining of cellulose nanocrystals from *Agave tequilana* weber Var. Azul bagasse by acid hydrolysis	https://doi.org/10.3390/nano11020520
136	[145]	Preparation and characterization of corn husk nanocellulose coating on electrospun polyamide 6	https://doi.org/10.1016/j.aej.2021.10.011
137	[146]	Cytocompatible cellulose nanofibers from invasive plant species *Agave americana* L. and *Ricinus communis* L.: a renewable green source of highly crystalline nanocellulose	https://doi.org/10.1631/jzus.B2000683
138	[147]	Production of new cellulose nanomaterial from red algae marinebiomass *Gelidium elegans*	http://dx.doi.org/10.1016/j.carbpol.2016.06.083
139	[148]	Extraction and characterization of α-cellulose-rich residue from maize (*Zea mays* L.) husk	http://localhost:8080/xmlui/handle/123456789/1021

No	First Author	Study Title	DOI
140	[149]	Cellulose fibers extracted from rice and oat husks and their application in hydrogel	http://dx.doi.org/10.1016/j.foodchem.2016.10.048

Table S2. Estimated (bold) and tabulated (normal font) density values correspond to different chemical concentrations. Tabulated density data were obtained from references 1 to 4 and the link provided below the Table.

Sulfuric acid

Concentration (% w/w)	Density (g/cm^3) at 20 °C [1]
28	1.2023
**28.8**	**1.2088**
50	1.3951
**50.5**	**1.4000**
51	1.4049
**51.5**	**1.4099**
**51.6**	**1.4108**
52	1.4148
**52.5**	**1.4198**
53	1.4248
59	1.4875
**59.5**	**1.4929**
60	1.4983
61	1.5091
62	1.5200
**62.4**	**1.5244**
**62.5**	**1.5255**
63	1.5310
**63.5**	**1.5366**
**63.8**	**1.5399**
**63.9**	**1.5410**
64	1.5421
**64.61**	**1.5489**
65	1.5533
70	1.6105
76	1.6810
**76.2**	**1.6833**
77	1.6927

Formic acid

Concentration (% w/w)	Density (g/cm^3) at 20 °C [1]
83	1.1914
**83.55**	**1.1922**
84	1.1929
**84.93**	**1.1951**
86	1.1976
**86.07**	**1.1977**
89	1.2028
**89.36**	**1.2034**
90	1.2044

Formic acid

Concentration (% w/w)	Density (g/cm^3) at 20 °C [1]
**92.62**	**1.2091**
94	1.2117
**94.35**	**1.2125**
96	1.2158
**96.14**	**1.2160**
97	1.217
**97.06**	**1.2171**

Citric acid

Concentration (% w/w)	Density (g/cm^3) at 20 °C [1, 2]
59.52	1.2980
**60**	**1.3010**
62.44	1.3159
64.99	1.3314
65.31	1.3336
66.50	1.3431
70.32	1.3670
**76**	**1.4045**
**80**	**1.4312**

Sodium hydroxide

Concentration (% w/w)	Density (g/cm^3) at 20 °C [1]
7	1.0758
**7.5**	**1.0814**
8	1.0869
17	1.1861
**17.5**	**1.1916**
18	1.1971

Hydrogen peroxide

Concentration (% w/w)	Density (g/cm^3) at 18 °C [1]
2	1.0058
**3**	**1.0095**
4	1.0131
**4.5**	**1.0149**
**5**	**1.0168**
6	1.0204
**7.5**	**1.0259**
8	1.0277
**9.25**	**1.0323**

Hydrogen peroxide

Concentration (% w/w)	Density (g/cm^3) at 18 °C [1]
**12.34**	**1.0438**
**13.5**	**1.0481**
14	1.0499
**15**	**1.0537**
**15.42**	**1.0552**
**16**	1.0574

Sodium hypochlorite

Concentration (% w/w)	Density (g/cm^3) at 20 °C [3]
0	0.9980
**0.5**	**0.9995**
1	1.0010
1.5	1.0100
**1.7**	**1.0128**
2	1.0170
0	0.9980
3	1.0330
3.1	1.0350
**3.2**	**1.0362**
6.3	1.0850
**6.5**	**1.0880**
6.7	1.0910
10	1.1430
10.4	1.1500
**10.6**	**1.1530**
10.8	1.1560

Sodium chlorite

Concentration (% w/w)	Density (g/cm^3) at 25 °C [4]
**0.7**	**1.0005**
**1.4**	**1.0040**
**1.5**	**1.0045**
**1.7**	**1.0055**
2	1.0070
7	1.0668
8	1.0768
**8.5**	**1.0818**
9	1.0868

**90.96**	**1.2058**	10	1.0351
92	1.2078	12	1.0425

1: Tabulated (normal, non-bold) density data were obtained from the chemistry reference books [1,2]; 2: Tabulated (normal, non-bold) density data were obtained from the chemistry reference books [1,2] and [3]; 3: Tabulated (normal, non-bold) density data were obtained online [4]; 4: Tabulated (normal, non-bold) density data were obtained from Occidenta Chemical Corporation OxyChem [5].

Table S3. The price of the chemical from two reputable suppliers, Sigma-Aldrich and Fisher Scientific. Items in blue were chosen for the comparison and selecting the best deal (Table S5).

**[8]:** https://www.sigmaaldrich.com/ (Prices in Euro)			**[9]:** https://www.fishersci.com/ (Prices in US. Dollars)		
**Chemicals**	**Price**		**Chemicals**	**Price**	
	**1 L or Kg**	**2.5 L or Kg**		**1 L or Kg**	**2.5 L or Kg**
**Sodium hydroxide**					
221465/ Sodium hydroxide ACS reagent, ≥97.0%, pellets	171	278	Thermo Scientific Chemicals Sodium hydroxide, 97+%, ACS reagent, pellets	184.80	251
S5881/ Sodium hydroxide reagent grade, ≥98%, pellets (anhydrous)	58.40		Thermo Scientific Chemicals Sodium hydroxide, 98%, extra pure, pellets	128.2	269
			Sodium Hydroxide (Pellets/Certified ACS), Fisher Chemical™	322.50	
			Thermo Scientific Chemicals Sodium hydroxide, pellets, 98%	45.99 (500 g)	
**Potassium hydroxide**					
221473/ Potassium hydroxide ACS reagent, ≥85%, pellets	60.30	156	Thermo Scientific Chemicals Potassium hydroxide, ca. 85%, ACS reagent, pellets	74.70 (500 g)	204
484016/ Potassium hydroxide reagent grade, 90%, flakes	73.60		LabChem, Inc. Potassium Hydroxide, ACS Grade, >85.0%, LabChem™	132.3	
			Spectrum Chemical Manufacturing Corporation Potassium Hydroxide, Pellets, ACS, 85%, Spectrum™ Chemical	249.5	431
			Thermo Scientific Chemicals Potassium hydroxide, ca. 85%, for analysis, pellets	112.6	268
			Potassium Hydroxide (Pellets/NF/FCC), Fisher Chemical™	221 (500 g)	
			Fisher Scientific Potassium Hydroxide (Pellets/Certified ACS), Fisher Chemical™	307.50	
**Hydrogen peroxide**					
216763/ Hydrogen peroxide solution contains inhibitor, 30 wt. % in H_2O, ACS reagent	85.30 (500 mL)		Fisher Chemical Hydrogen Peroxide, 30% (Certified ACS), Thermo Scientific™	328 (500 mL)	

[8]: https://www.sigmaaldrich.com/ (Prices in Euro)			[9]: https://www.fishersci.com/ (Prices in US. Dollars)		
Chemicals	Price		Chemicals	Price	
	1 L or Kg	2.5 L or Kg		1 L or Kg	2.5 L or Kg
1.07210/ Hydrogen peroxide 30%, (Perhydrol™) (stabilized for higher storage temp.) for analysis EMSURE ISO	102	201	Ricca Chemical Company Hydrogen Peroxide, 30% (w/w), ACS Reagent Grade, Ricca Chemical	554.5	
H1009/ Hydrogen peroxide solution 30 % (w/w) in H_2O, contains stabilizer	194 (500 mL)		Reagents Holdings Llc Hydrogen Peroxide, 30% w/w, ACS Reagent, Reagents	301	
1.08597/Hydrogen peroxide 30%, stabilized, EMPROVE® ESSENTIAL, Ph. Eur., BP, USP	36	70.9	Hydrogen Peroxide, 30% w/w, ACS Guaranteed Reagent), MilliporeSigma™	350.5	
**Sodium hypochlorite**					
425044/ Sodium hypochlorite solution reagent grade, available chlorine 10-15 %	156		Thermo Scientific Chemicals Sodium hypochlorite, 10-15% active chlorine	103.50 (500 mL)	158.10
13440/ Sodium hypochlorite solution 6-14% active chlorine basis	82 (500 ml)		Honeywell Chemicals Sodium hypochlorite solution, Reagent grade, Available chlorine 10 to 15%, Honeywell	237	
**Sodium chlorite**					
244155/ Sodium chlorite technical grade, 80%	247		Honeywell Chemicals Sodium Chlorite, puriss p.a, 80% (RT), Honeywell Fluka™	306.53	
71388/ Sodium chlorite puriss. p.a., 80% (RT)	235		LabChem, Inc. Sodium Chlorite, Technical Grade, 80%, LabChem™	314.50 (500 g)	
			Ricca Chemical Company Sodium Chlorite, 80%, Technical Grade, Ricca Chemical	419 (500 g)	
**Hydrochloric acid**					
258148/ Hydrochloric acid 37%, ACS reagent	-	107, 147	Hydrochloric Acid, Certified ACS Plus, 36.5 to 38.0%, Fisher Chemical™	146.40 (500 mL)	
320331/ Hydrochloric acid 37%, ACS reagent	-	270	LabChem, Inc. Hydrochloric Acid, ACS Grade, 36.5 to 38.0%, LabChem™	100.60	140.40, 164.80
1.00317/ Hydrochloric acid, 37%, for analysis EMSURE® ACS, ISO, Reag. Ph Eur	27.80, 30.60	60, 62.80			
**Sulfuric acid**					
258105/ Sulfuric acid, ACS reagent, 95.0-98.0%	120	167, 198	Honeywell Chemicals Sulfuric Acid, ACS Reagent, 95.0 to 98.0%, Honeywell Fluka™	156.01, 117.84	167.68
30743/ Sulfuric acid, puriss. p.a., for determination of Hg, ACS reagent, reag. ISO, reag. Ph. Eur., 95.0-97.0%	71.80, 73.20	145	Sulfuric Acid ACS, 95 to 98%, MilliporeSigma™		162.10

[8]: https://www.sigmaaldrich.com/ (Prices in Euro)			[9]: https://www.fishersci.com/ (Prices in US. Dollars)		
Chemicals	Price		Chemicals	Price	
	1 L or Kg	2.5 L or Kg		1 L or Kg	2.5 L or Kg
1.00731/ Sulfuric acid, 95-97%, for analysis EMPARTA® ISO	50.30, 55.30	98.60, 108, 113			
1.00732/ Sulfuric acid, 95-97%, for analysis (max. 0.005 ppm Hg) EMSURE® ACS,ISO,Reag. Ph Eur	77.40	151, 158			
**Phosphoric acid**					
695017/ Phosphoric acid, ACS reagent, ≥85 wt. % in H_2O	-	238	LabChem, Inc. Phosphoric Acid, ACS Grade, ≥ 85.0% (w/w), LabChem™	128 (500 mL)	240, 259.50
1.00573/ Phosphoric acid,85%, for analysis EMSURE® ACS,ISO,Reag. Ph Eur	101	199, 228	Ricca Chemical Company Phosphoric Acid, ACS Reagent Grade, Ricca Chemical	167.3	464
438081/ Phosphoric acid, ACS reagent, ≥85 wt. % in H_2O	-	329	Thermo Scientific Chemicals Phosphoric acid, extra pure, 85% solution in water	125	264
30417/ Phosphoric acid, puriss. p.a., ACS reagent, reag. ISO, reag. Ph. Eur., ≥85%	120	276	Thermo Scientific Chemicals Phosphoric acid, for analysis, 85 wt% solution in water	135.5	234
30417-M/ Phosphoric acid, puriss. p.a., ACS reagent, reag. ISO, reag. Ph. Eur., ≥85%	107	247			
**Citric acid**					
251275/ Citric acid, ACS reagent grade, ≥99.5%, crystals	163	295	Thermo Scientific Chemicals Citric acid, anhydrous, ACS, 99.5+%	87.60 (500 g)	
791725/ Citric acid, anhydrous grade, ACS reagent grade, ≥99.5%, Redi-Dri™, free-flowing, crystals	175	367	Thermo Scientific Chemicals Citric acid, 99%, pure, anhydrous	120	
			Fisher Chemical Citric Acid Anhydrous (Crystalline), Fisher BioReagents	103.90 (500 g)	
			Thermo Scientific Chemicals Citric acid, anhydrous, ACS, 99.5+%	175.2	
**Lactic acid**					
W261114/ Lactic acid, natural, ≥85%	83.40		Thermo Scientific Chemicals L(+)-Lactic acid, 90% solution in water	195.60	
252476/Lactic acid ≥85% in H_2O, solution, ACS reagent grade	176	355	Thermo Scientific Chemicals Lactic acid, ACS, 85.0-90.0% aq. soln.	105.90 (500 g)	
			Sigma Aldrich Fine Chemicals Biosciences Lactic acid natural, >=85% | 50-21-5 | MFCD00004520 | 1KG	106.01	

[8]: https://www.sigmaaldrich.com/ (Prices in Euro)			[9]: https://www.fishersci.com/ (Prices in US. Dollars)		
**Chemicals**	**Price**		**Chemicals**	**Price**	
	**1 L or Kg**	**2.5 L or Kg**		**1 L or Kg**	**2.5 L or Kg**
			Lactic Acid (85% w/w/Certified ACS), Fisher Chemical™	800	
**Formic acid**					
F0507/ Formic acid reagent grade, ≥95%	115		Thermo Scientific Chemicals Formic acid, 88%, ACS reagent		333
33015/ Formic acid puriss. p.a., ACS reagent, reag. Ph. Eur., ≥98%	75.80	128	Formic Acid ACS, 98%, MilliporeSigma™	492.50	
695076/ Formic acid ACS reagent, ≥96%	57.10 (500 ml)		Thermo Scientific Chemicals Formic acid, ACS, 96+%	125.50 (500 mL)	
			Honeywell Chemicals Formic acid, Puriss. p.a., ACS Reagent, Reag. Ph. Eur., ≥98%, Honeywell Fluka™	282.17	587.69
			Formic Acid, 88% (Certified ACS), Fisher Chemical™	347 (500 mL)	

Table S4. The selected chemicals with their corresponding prices (per kilogram or liter in US dollars) from the two reputable suppliers, Sigma-Aldrich and Fisher Scientific. The final selected chemicals based on the best prices (best deal) are shown in bold.

**Sigma Aldrich:** https://www.sigmaaldrich.com/ (Prices in US. Dollars)		**Fisher Scientific:** https://www.fishersci.com/ (Prices in US. Dollars)	
**Chemicals**	**Price (Kg or L)**	**Chemicals**	**Price (Kg or L)**
**Sodium hydroxide**			
221465/ Sodium hydroxide ACS reagent, ≥97.0%, pellets	198.36	Thermo Scientific Chemicals Sodium hydroxide, 97+%, ACS reagent, pellets	184.80
**S5881/ Sodium hydroxide reagent grade, ≥98%, pellets (anhydrous)**	**67.74**	Thermo Scientific Chemicals Sodium hydroxide, 98%, extra pure, pellets	128.2
**Potassium hydroxide**			
**221473/ Potassium hydroxide ACS reagent, ≥85%, pellets**	**69.95**	LabChem, Inc. Potassium Hydroxide, ACS Grade, >85.0%, LabChem™	132.3
		Spectrum Chemical Manufacturing Corporation Potassium Hydroxide, Pellets, ACS, 85%, Spectrum™ Chemical	249.5
		Thermo Scientific Chemicals Potassium hydroxide, ca. 85%, for analysis, pellets	112.6
**Hydrogen peroxide**			
1.07210/ Hydrogen peroxide 30%, (Perhydrol™) (stabilized for higher storage temp.) for analysis EMSURE ISO	118.32	Ricca Chemical Company Hydrogen Peroxide, 30% (w/w), ACS Reagent Grade, Ricca Chemical	554.5
**1.08597/Hydrogen peroxide 30%, stabilized, EMPROVE® ESSENTIAL, Ph. Eur., BP, USP**	**41.76**	Reagents Holdings Llc Hydrogen Peroxide, 30% w/w, ACS Reagent, Reagents	301
		Hydrogen Peroxide, 30% w/w, ACS Guaranteed Reagent), MilliporeSigma™	350.5
**Sodium hypochlorite**			
**425044/ Sodium hypochlorite solution reagent grade, available chlorine 10-15 %**	**180.96**	Honeywell Chemicals Sodium hypochlorite solution, Reagent grade, Available chlorine 10 to 15%, Honeywell	237
**Sodium chlorite**			
244155/ Sodium chlorite technical grade, 80%	286.52	Honeywell Chemicals Sodium Chlorite, puriss p.a, 80% (RT), Honeywell Fluka™	306.53
**71388/ Sodium chlorite puriss. p.a., 80% (RT)**	**272.6**		
**Hydrochloric acid**			
**1.00317/ Hydrochloric acid, 37%, for analysis EMSURE® ACS, ISO, Reag. Ph Eur**	**32.25**	LabChem, Inc. Hydrochloric Acid, ACS Grade, 36.5 to 38.0%, LabChem™	100.60

**Sigma Aldrich:** https://www.sigmaaldrich.com/ (Prices in US. Dollars)		**Fisher Scientific:** https://www.fishersci.com/ (Prices in US. Dollars)	
**Chemicals**	**Price (Kg or L)**	**Chemicals**	**Price (Kg or L)**
1.00317/ Hydrochloric acid, 37%, for analysis EMSURE® ACS, ISO, Reag. Ph Eur	35.50		
**Sulfuric acid**			
258105/ Sulfuric acid, ACS reagent, 95.0-98.0%	139.20	Honeywell Chemicals Sulfuric Acid, ACS Reagent, 95.0 to 98.0%, Honeywell Fluka™	156.01
30743/ Sulfuric acid, puriss. p.a., for determination of Hg, ACS reagent, reag. ISO, reag. Ph. Eur., 95.0-97.0%	83.29	Honeywell Chemicals Sulfuric Acid, ACS Reagent, 95.0 to 98.0%, Honeywell Fluka™	117.84
30743/ Sulfuric acid, puriss. p.a., for determination of Hg, ACS reagent, reag. ISO, reag. Ph. Eur., 95.0-97.0%	84.91		
**1.00731/ Sulfuric acid, 95-97%, for analysis EMPARTA® ISO**	**58.35**		
1.00731/ Sulfuric acid, 95-97%, for analysis EMPARTA® ISO	64.15		
1.00732/ Sulfuric acid, 95-97%, for analysis (max. 0.005 ppm Hg) EMSURE® ACS,ISO,Reag. Ph Eur	89.78		
**Phosphoric acid**			
**1.00573/ Phosphoric acid,85%, for analysis EMSURE® ACS,ISO,Reag. Ph Eur**	**117.16**	Thermo Scientific Chemicals Phosphoric acid, extra pure, 85% solution in water	125
30417/ Phosphoric acid, puriss. p.a., ACS reagent, reag. ISO, reag. Ph. Eur., ≥85%	139.20	Thermo Scientific Chemicals Phosphoric acid, for analysis, 85 wt% solution in water	135.5
30417-M/ Phosphoric acid, puriss. p.a., ACS reagent, reag. ISO, reag. Ph. Eur., ≥85%	124.12		
**Citric acid**			
251275/ Citric acid, ACS reagent grade, ≥99.5%, crystals	189.08	**Thermo Scientific Chemicals Citric acid, 99%, pure, anhydrous**	**120**
791725/ Citric acid, anhydrous grade, ACS reagent grade, ≥99.5%, Redi-Dri™, free-flowing, crystals	203.00	Thermo Scientific Chemicals Citric acid, anhydrous, ACS, 99.5+%	175.2
**Lactic acid**			
**W261114/ Lactic acid, natural, ≥85%**	**96.74**	Sigma Aldrich Fine Chemicals Biosciences Lactic acid natural, >=85% | 50-21-5 | MFCD00004520 | 1KG	106.01
252476 /Lactic acid ≥85% in H_2O, solution, ACS reagent grade	204.16		
**Formic acid**			

**Sigma Aldrich:** https://www.sigmaaldrich.com/ (Prices in US. Dollars)		**Fisher Scientific:** https://www.fishersci.com/ (Prices in US. Dollars)	
**Chemicals**	**Price (Kg or L)**	**Chemicals**	**Price (Kg or L)**
F0507/ Formic acid reagent grade, ≥95%	133.40	Honeywell Chemicals Formic acid, Puriss. p.a., ACS Reagent, Reag. Ph. Eur., ≥98%, Honeywell Fluka™	282.17
**33015/ Formic acid puriss. p.a., ACS reagent, reag. Ph. Eur., ≥98%**	**87.93**		

Table S5. Calculated prices for different concentration distribution ranges of each chemical across three stages of the CNC isolation process. Reagent purity was considered in all price calculations.

Chemical (purity)	Price per L or Kg	Density (g/mL)	Concentration Range (M or N)	Required Chemical (g or mL)	Cost Range (USD $)
**NaOH (≥98% w/w)**	67.74 (1 Kg)	NR [1]	< 0.78 M<br>0.78 – 3.16 M<br>> 3.16 – 6.25 M	< 31.83 g<br>31.83 – 128.97 g<br>> 128.97 – 255.08 g	< 2.16<br>2.16 – 8.74<br>> 8.74 – 17.28
**KOH (≥85% w/w)**	69.95 (1 Kg)	NR [1]	< 0.82 M<br>0.82 – 1.34 M<br>> 1.34 – 1.78 M	< 54.13 g<br>54.13 – 88.45 g<br>> 88.45 – 117.49 g	< 3.79<br>3.79 – 6.19<br>> 6.19 – 8.22
**H_2O_2 (30% w/w)**	41.76 (1 L)	1.112	< 2.06 M<br>2.06 - 6.04 M<br>> 6.04 - 11.66	< 210.01 mL<br>210.01 – 615.75 mL<br>> 615.75 – 1188.68 mL	< 8.77<br>8.77 – 25.71<br>> 25.71 – 49.64
**NaClO (10-15% w/w Cl_2)**	180.96 (1 L)	1.194	< 0.59 M<br>0.59 - 0.97 M<br>> 0.97 – 4 M	< 280.31 mL<br>280.31– 460.84 mL<br>> 460.84 – 1900.38 mL	< 50.72<br>50.72 – 83.39<br>> 83.39 – 343.89
**$NaClO_2$ (80% w/w)**	272.6 (1 Kg)	NR [1]	< 0.14 M<br>0.14 - 0.47 M<br>> 0.47 – 1.4 M	< 15.83 g<br>15.83 – 53.14 g<br>> 53.14 – 158.27 g	< 4.32<br>4.32 – 14.49<br>> 14.49 – 43.15
**H_2SO_4 (95-97% w/w)**	58.35 (1 L)	1.835	< 16.01 N<br>16.01 – 19.36 N<br>> 19.36 - 35.93 N	< 455.57 mL<br>455.57– 538.80 mL<br>> 538.80 – 1000 mL	< 26.00<br>26.00 – 31.44<br>> 31.44 – 58.35
**HCl (37% w/w)**	32.25 (1 L)	1.184	< 5.42 N<br>5.42 – 10.15 N<br>> 10.15 – 10.72 N	< 451.16 mL<br>451.16 – 844.88 mL<br>> 844.88 – 892.33 mL	< 14.55<br>14.55 – 27.25<br>> 27.25 – 28.78
**H_3PO_4 (85% w/w)**	117.16 (1 L)	1.689	< 26.02 N<br>26.02 – 27 N<br>> 27 – 32.1 N	< 592.03 mL<br>592.03 – 614.32 mL<br>> 614.32 – 730.36 mL	< 69.36<br>69.36 – 71.97<br>> 71.97 – 85.57
**Citric acid (99% w/w)**	120 (1 Kg)	NR [1]	< 7.69 N<br>7.69 – 14.93 N<br>> 14.93 - 18 N	< 497.45 g<br>497.45 – 965.80 g<br>> 965.80 – 1164.39 g	< 59.69<br>59.69 – 115.90<br>> 115.90 – 139.73
**Lactic acid (≥85% w/w)**	96.74 (1 L)	1.210	< 5.50 N<br>5.50 – 6.50 N<br>> 6.50 – 7.00 N	< 481.70 mL<br>481.70 – 569.28 mL<br>> 569.28 – 613.07 mL	< 46.60<br>46.60 – 55.07<br>> 55.07 – 59.31
**Formic acid (≥98% w/w)**	87.93 (1 L)	1.218	< 14.70 N<br>14.70 – 25.94 N	< 566.68 mL<br>566.68 – 999.98 mL	< 49.83<br>49.83 – 87.93

Table S6. Price categories and corresponding penalty points for different concentration distribution ranges of each chemical across three stages of the CNC isolation process. Reagent purity was considered in all price calculations.

Chemical (purity)	Price per L or Kg	Cost Range (USD $)	Unified (cross-chemical type) price categories	Within the chemical-type price category: penalty point to be assigned
		< 2.16	LOW ($ 0–30)	Low 1 ($ 0–5): +0.5
**NaOH (≥ 98% w/w)**	67.74 (1 Kg)	2.16 – 8.74	LOW ($ 0–30)	Low 2 ($ > 5–15): +1
		> 8.74 - 17.28	LOW ($ 0–30)	Low 3 ($ > 15–30): +1.5
		< 3.79	LOW ($ 0–30)	Low 1 ($ 0–5): 0.5
**KOH (≥ 85% w/w)**	69.95 (1 Kg)	3.79 – 6.19	LOW ($ 0–30)	Low 2 ($ > 5–15): +1
		> 6.19 – 8.22	LOW ($ 0–30)	Low 2 ($ > 5–15): +1
		< 8.77	LOW ($ 0–30)	Low 2 ($ > 5–15): +1
**H_2O_2 (30% w/w)**	41.76 (1 L)	8.77 – 25.71	LOW ($ 0–30)	Low 3 ($ > 15–30): +1.5
		> 25.71 - 49.64	MEDIUM ($ >30–60)	Medium 2 (> 40–50): +2.5
		< 50.72	MEDIUM ($ >30–60)	Medium 3 (> 50–60): +3
**NaClO (10-15% w/w Cl_2)**	180.96 (1 L)	50.72 – 83.39	HIGH ($ >60–90)	High 3 (> 80–90): +4.5
		> 83.39 - 343.89	VERY HIGH ($ >90–350)	Very High 4 (> 250–350): +6.5
		< 4.32	LOW ($ 0–30)	Low 1 ($ 0–5): +0.5
**$NaClO_2$ (80% w/w)**	272.6 (1 Kg)	4.32 – 14.49	LOW ($0–30)	Low 2 ($ > 5–15): +1
		> 14.49 - 43.15	MEDIUM ($ >30–60)	Medium 2 (> 40–50): +2.5
		< 26.00	LOW ($0–30)	Low 3 ($ > 15–30): +1.5
**H_2SO_4 (95-97% w/w)**	58.35 (1 L)	26.00 – 31.44	MEDIUM ($>30–60)	Medium 1 (> 30–40): +2
		> 31.44 - 58.35	MEDIUM ($ >30–60)	Medium 3 (> 50–60): +3
		< 14.55	LOW ($ 0–30)	Low 2 ($ > 5–15): +1
**HCl (37% w/w)**	32.25 (1 L)	14.55 – 27.25	LOW ($ 0–30)	Low 3($ > 15–30): +1.5
		> 27.25 - 28.78	LOW ($0–30)	Low 3 ($ > 15–30): +1.5
		< 69.36	HIGH ($>60–90)	High 1 (> 60–70): +3.5
**H_3PO_4 (85% w/w)**	117.16 (1 L)	69.36 – 71.97	HIGH ($ >60–90)	High 2 (> 70–80): +4
		> 71.97 - 85.57	HIGH ($ >60–90)	High 3 (> 80–90): +4.5
		< 59.69	MEDIUM ($>30–60)	Medium 3 (> 50–60): +3
**Citric acid (99% w/w)**	120 (1 Kg)	59.69 – 115.90	VERY HIGH ($ >90–350)	Very High 1 (> 90–120): +5
		> 115.90 - 139.73	VERY HIGH ($ >90–350)	Very High 2 (> 120–150): +5.5
		< 46.60	MEDIUM ($>30–60)	Medium 2 (> 40–50): +2.5
**Lactic acid (≥ 85% w/w)**	96.74 (1 L)	46.60 – 55.07	MEDIUM ($>30–60)	Medium 3 (> 50–60): +3
		> 55.07 - 59.31	MEDIUM ($ >30–60)	Medium 3 (> 50–60): +3
**Formic acid (≥ 98% w/w)**	87.93 (1 L)	< 49.83	MEDIUM ($ >30–60)	Medium 2 (> 40–50): +2.5
		49.83 – 87.93	HIGH ($ >60–90)	High 3 (> 80–90): +4.5

Table S7. Post-use environmental burden of the chemicals used in CNC isolation in wastewater streams. Please refer to Table S8-S10 for Representative SDS human hazard classes for each chemical.

Chemicals	Main residual ion(s)/by-products	Major post-use burden(s)	Relative severity rank [1]
**Alkali agents**			
Potassium hydroxide	Potassium cation (K^+), Hydroxyl anion (OH^-)	• Increasing wastewater pH → affecting the nitrogen cycle<br>• Scaling issue in wastewater treatment plants by the formation of metal-OH precipitates<br>• Nutrient concern affecting surface water quality if the water is potassium-rich<br>• The second most abundant nutrient for plants, promoting their growth	2- Low (increasing alkalinity, scaling issue, but a favorable nutrient for plant growth)
Sodium hydroxide	Sodium cation (Na^+), Hydroxyl anion (OH^-)	• Increasing wastewater pH → affecting the nitrogen cycle<br>• Scaling issue in wastewater treatment plants by the formation of metal-OH precipitates<br>• The core cation responsible for soil structure disturbance → affects plants adversely if irrigated with reclaimed water containing high sodium ions	4- Moderate (increasing alkalinity, scaling issue, affecting irrigation water quality, soil structure, and crop yield)
**Bleaching agents**			
Hydrogen peroxide	Decomposes to H_2O & O_2 rather than ionization	• Non-chlorinated bleach<br>• Decomposes safely (to oxygen and water)	1- Very low (no persistent by-product)
Sodium chlorite	Chlorine dioxide (CLO_2) Chlorite (ClO_2^-) and Chlorate (ClO_3^-)	• May contain chlorate (regulated compound) as an impurity, and form chlorate after dissociation<br>• Form CLO_2 gas, which oxidizes the target materials, and chlorite → both can turn into chlorate<br>• Does not chlorinate organic matter (limited haloacetic acid (HAAs), trihalomethanes (THM4) production)	4- Moderate (chlorinated by-product (chlorate and chlorite are regulatory substances, but generate limited carcinogenic/toxic DBPS)

Sodium hypochlorite	Chlorine (Cl_2) hypochlorous acid (HOCl), Hypochlorite (OCl^-), and Chlorate (ClO_3^-)	• May contain chlorate (regulated compound) as an impurity, and form chlorate after dissociation<br>• Age to chlorate and perchlorate (persistent compounds) in storage (their level increases with time and temperature)<br>• Form disinfection byproducts (DBPs), such as haloacetic acid (HAAs), trihalomethanes (THM4), in reaction with natural organic matter. DBPs are regulated compounds with toxic and carcinogenic effects.	5- High (formation of recalcitrant chlorinated by-product with aging and after use (with chlorate as a regulated ion), formation of toxic carcinogenic DBPs production)
**Organic acids**			
Formic acid	Formate ion ($HCOO^-$)	• Monotropic<br>• Simplest organic acid, readily biodegradable (biologically consumed)	1- Very low (Readily biodegradable, minimal residue, lowest environmental burden)
Lactic acid	Lactate ion ($CH_3CH(OH)COO^-$)	• Monotropic<br>• Second simple organic acid, readily biodegradable (biologically consumed)	1- Very low: Comparable to formic acid
Citric acid	Citrate ions: H_2Cit^-, $HCit^{2-}$, Cit^{3-}	• Triprotic<br>• Readily biodegradable (biologically consumed, but the hardest compared to other acids listed above)<br>• Chelating agent → mobilized heavy metals	3- Mild (Slightly higher oxygen demand for biological reactions, chelating behavior, but still a mild burden)
**Inorganic acids**			
Hydrochloric acid (HCl)	Chloride (Cl^-)	• Corrosion of metals in pipelines & concrete deterioration<br>• No effluent guideline limit → possible accumulation in reuse water → salinity stress on plants irrigated with reclaimed water	4- Moderate (persistent & corrosive, but not eutrophic or odorous, salinity stress on crops).
Sulfuric acid (H_2SO_4)	Sulfate (SO_4^{2-})	• Odor via H_2S formation<br>• Concrete corrosion through the biogenic sulfuric acid cycle<br>• Sulfate scaling in pipes/membranes	5- High (persistent, corrosive, odorous, & scaling risk).

Phosphoric acid (H_3PO_4)	Orthophosphate (PO_4^{3-} / HPO_4^{2-} / $H_2PO_4^{-}$)	• Eutrophication of surface waters<br>• Struvite scaling in pipes and wastewater treatment infrastructures<br>• Strict global effluent regulations limit	6- Very high (persistent, strictly regulated nutrient pollutant & scaling risk).

1: The lower the rank, the lower the post-use environmental burden (larger values = more burden).

Table S8. Measured values from standard environmental and animal (in vivo) toxicological tests for alkaline chemicals. Toxicity endpoints compared between the two alkaline chemicals are shown in bold.

Chemical	Key aquatic endpoints [1]	Environmental toxicity severity (penalty point/rank) [2]	Key human hazard classes [3] and Toxicity endpoints derived from animal studies [1]	Animal toxicity severity (penalty point/rank) [2]	Reference
**Sodium hydroxide**	Acute aquatic toxicity: 3 (harmful)<br><br>**Fish (Mosquito fish: *Gambusia affinis*, LC50, 96 h, mg/L): 125** [4]<br>Fish (Rainbow trout: *Oncorhynchus mykiss*, LC50, 96 h, mg/L): 45.4 [7]<br>**Water flea microcrustaceans (*Ceriodaphnia,* LC50, 48 h, mg/L): 40.4** [5]<br>**Water flea microcrustaceans (*Ceriodaphnia pulchella,* LC50, 48 h, mg/L): 40** [7]<br><br>**Algae (IC50**, **72 h, mg/L): 40.4** [5, 7]<br>Bacteria (*Photobacterium phosphoreum*, EC50, 15 min, mg/L): 22 [7]	**Fish: 1**<br>**Water flea (Daphnia): 2**	Corrosive to metal: 1<br>Skin corrosion: 1A<br>Eye damage: 1 (serious)<br>Specific target organ toxicity (repeated exposure): 3<br>Acute toxicity (oral): Severe burns<br>**Acute toxicity (oral, rat, LD50, mg/kg): 40** [7]**, 140**-340 [7]**, 325** [7]<br>Acute toxicity (dermal, rabbit, LD50, mg/kg): 1350 [7]	**Oral: 2**	[150-154]
**Potassium hydroxide**	**Fish (Mosquito fish: *Gambusia affinis*, LC50, 96 h, mg/L): 80** [4, 7]<br><br>**Water flea microcrustaceans (*Ceriodaphnia dubia,* LC50, 48 h, mg/L): 56** [7]	**Fish: 2**<br>**Water flea (Daphnia): 1**	Corrosive to metal: 1<br>Skin corrosion: 1A<br>Eye damage: 1 (serious)<br>Acute toxicity (oral): 3 (Harmful)<br>Specific target organ toxicity (single & repeated exposure, respiratory system): 2<br>Aspiration hazard: 1<br>**Acute toxicity (oral, rat, LD50, mg/kg): 333** [5,6]	**Oral: 1**	[155-160]

1: Data obtained from MSDS according to available sources for toxicological endpoint values. When multiple values were available from various reliable sources (e.g., ECHA, ECOTOX, OECD-based), the lowest value was selected to ensure a conservative approach. If both reliable and unverified (source not specified) data were available, the value from the reliable source was prioritized, even if the unverified source reported a more conservative figure. Values from unverified sources were used only when no reliable data were available; 2: The lower the rank/penalty point, the lower the toxicity (larger points = more toxicity); 3: Hazard classifications summarize the representative SDS sources used in this review and concern handling hazards. The numbers are MSDS-derived hazard categories, the lower the category number, the more toxic/hazardous the chemical is; 4: ECOTOX: U.S. EPA (U.S. Environmental Protection Agency Ecotoxicology Database): a collection of peer-reviewed ecotoxicological test results conducted according to internationally recognized (e.g., OECD or ASTM) test methods; 5: ECHA (European Chemicals Agency (EU REACH database) of OECD-based studies; 6: OECD (The Organization for Economic Co-operation and Development) standard test guideline; 7: Source from which the toxicological values are retrieved, not provided; LC50: Lethal Concentration 50%; LD50: Lethal Dose, 50%.

Table S9. Measured values from standard environmental and animal (in vivo) toxicological tests for bleaching chemicals. Toxicity endpoints compared across different bleaching agents are shown in bold.

Chemical	Key aquatic endpoints [1]	Environmental toxicity severity (penalty point/rank) [2]	Key human hazard classes [3] and Toxicity endpoints derived from animal studies [1]	Animal toxicity severity (penalty point/rank) [2]	Reference
**Sodium chlorite**	Acute aquatic toxicity: 1 (very toxic)<br>Chronic aquatic toxicity: 1 (very toxic)<br><br>**Fish (Sheepshead Minnow: *Cyprinodon variegatus,* LC50, 96 h, mg/L): 105** [4, 5]<br>**Fish (Rainbow Trout: *Oncorhynchus mykiss,* LC50, 96 h, mg/L): 100** [7]<br>**Daphnia (*Daphnia magna*, EC50, 48 h, mg/L): < 1** [5, 6]<br>**Algae (*Pseudokirchneriella subcapitata*, IC50, 72 h,** 96 h, **mg/L): 21.5** [6], 1 [5]<br>Bacteria (activated sludge, 3 h, mg/L): > 100 [5, 6]	**Fish: 1**<br>**Daphnia: 2**<br>**Algae: 1**<br>Total points: 4	Oxidizing solid: 1<br>Skin corrosion: 1B<br>Eye damage: 1 (serious)<br>Specific target organ toxicity (repeated exposure): 2<br>Acute toxicity (oral): 3 (50-300 [7] mg/kg)<br>Acute toxicity (dermal): 2 (50-200 [7] mg/kg)<br>**Acute toxicity (oral, rat, LD50, mg/kg): 284** [5, 6]<br>**Acute toxicity (dermal, rabbit, LD50, mg/kg): 134** [4, 5]<br>**Acute toxicity (inhalation, rat, LC50, 4 h, mg/L): 0.230** [7]	**Oral: 3**<br>**Dermal: 3**<br>**Inhalation: 3**<br>Total points: 9	[161-165]
**Sodium hypochlorite**	Acute aquatic toxicity: 1 (very toxic)<br>Chronic aquatic toxicity: 1 (toxic)<br><br>**Fish (Fathead minnow: *Pimephales promelas*, LC50, 96 h, mg/L): 0.08** [4]<br>**Fish (Bluegill: *Lepomis macrochirus*, LC50, 96 h, mg/L): 0.4-0.8** [7], **0.28-1** [7]<br>**Fish (Rainbow trout: *Oncorhynchus mykiss*, LC50, 96 h, mg/L): 0.03-0.19** [7]<br>**Daphnia (*Daphnia magna*, EC50, 48 h, mg/L): 0.04** [4]<br>Water flea (*Ceriodaphnia cf. dubia*, EC50, 24 h, ug/L): 5 [7]	**Fish: 3**<br>**Daphnia: 3**<br>**Algae: 3**<br>Total points: 9	Corrosive to metal: 1<br>Skin corrosion: 1B<br>Eye damage: 1 (serious)<br>Specific target organ toxicity (single & repeated exposure): 3<br>**Acute toxicity (oral, rat, LD50, mg/kg): 1100** [6]<br>**Acute toxicity (dermal, rabbit, LD50, mg/kg): > 20000** [6, 7]<br>**Acute toxicity (inhalation, rat, LC50, 4 h,** 1 h, **mg/L): 2.3** [6]	**Oral: 1**<br>**Dermal: 1**<br>**Inhalation: 1**<br>Total points: 3	[166-169]

Chemical	Key aquatic endpoints [1]	Environmental toxicity severity (penalty point/rank) [2]	Key human hazard classes [3] and Toxicity endpoints derived from animal studies [1]	Animal toxicity severity (penalty point/rank) [2]	Reference
**Hydrogen peroxide**	**Algae (*Pseudokirchneriella subcapitata*, ErC50, 72 h, mg/L): 0.036 [6]**<br><br>Bacteria (activated sludge, 3 h, mg/L): 77.1 [5]<br>Chronic aquatic toxicity: 3 (harmful)<br><br>**Fish (Fathead minnow: *Pimephales promelas*, LC50, 96 h, mg/L): 16.4 [4, 5]**<br>**Fish (Rainbow Trout: *Oncorhynchus mykiss*, LC50, 96 h, mg/L): 10-32 [7]**<br>**Daphnia (*Daphnia pulex*, LC50, 48 h, mg/L): 2.4 [4]**<br>**Algae (Marine diatom: *Skeletonema costatum*, ErC50, 72 h, mg/L): 1.38 [5]**<br>**Algae (Nitzschia sp., ErC50, 72 h, mg/L): 0.85 [7]**<br>Bacteria (EC50, activated sludge, 30 min, 3 h, mg/L): 466 [5, 6], > 1000 [6]<br><br>Algae (Marine diatom: *Skeletonema costatum*, NOEC, 72 h, mg/L): 0.63 [5]<br>Chronic Daphnia (*Daphnia magna*, NOEC, 21 d, mg/L): 0.63 [5]	**Fish: 2**<br>**Daphnia: 1**<br>**Algae: 2**<br>Total points: 5	Oxidizing liquid: 2<br>Skin corrosion: 1A<br>Eye damage: 1 (serious)<br>Specific target organ toxicity (single & repeated exposure, respiratory system): 1<br>Acute toxicity (oral): 4 (500 [5] mg/kg);<br>Acute toxicity (dermal): 4<br>Acute toxicity (inhalation): 4 (11[5] mg/L, 4 h)<br>Acute toxicity (inhalation, Dusts, mists): 2<br>**Acute toxicity (oral, rat, LD50, mg/kg): 693.7 [5, 6]**<br>**Acute toxicity (dermal, rabbit, LD50, mg/kg): > 2000 [5, 6]**<br>**Acute toxicity (inhalation, rat, LD50, 4 h, mg/L): 2 [7]**	**Oral: 2**<br>**Dermal: 2**<br>**Inhalation: 2**<br>Total points: 6	[170-173]

1: Data obtained from MSDS according to available sources for toxicological endpoint values. When multiple values were available from various reliable sources (e.g., ECHA, ECOTOX, OECD-based), the lowest value was selected to ensure a conservative approach. If both reliable and unverified (source not specified) data were available, the value from the reliable source was prioritized, even if the unverified source reported a more conservative figure. Values from unverified sources were used only when no reliable data were available; 2: The lower the rank/penalty point, the lower the toxicity (larger points = more toxicity); 3: Hazard classifications summarize the representative SDS sources used in this review and concern handling hazards. The numbers are MSDS-derived hazard categories, the lower the category number, the more toxic/hazardous the chemical is; 4: ECOTOX: U.S. EPA (U.S. Environmental Protection Agency Ecotoxicology Database): a collection of peer-reviewed ecotoxicological test results conducted according to internationally recognized (e.g., OECD or ASTM) test methods; 5: ECHA (European Chemicals Agency (EU REACH database) of OECD-based studies; 6: OECD (The Organization for Economic Co-operation and Development) standard test guideline; 7: Source from which the toxicological values are retrieved, not provided; LC50: Lethal Concentration 50%; EC50: Effective Concentration 50%;

LD50: Lethal Dose, 50%; IC50: Inhibitory Concentration 50%; ErC50: Effective concentration (E_rC_{50}) that reduces the rate of growth by 50%; NOEC: No-Observed-Effect Concentration.

Table S10. Measured values from standard environmental and animal (in vivo) toxicological tests for organic and inorganic acids. Toxicity endpoints compared across different acids are shown in bold.

Chemical	Key aquatic endpoints [1]	Biodegradability	Environmental toxicity severity (penalty point/rank) [2]	Key human hazard classes [3] and Toxicity endpoints derived from animal studies [1]	Animal toxicity severity (penalty point/rank) [2]	Reference
			**Organic acids**			
**Formic acid**	Acute aquatic toxicity: 3<br><br>**Fish (Zebra fish: *Danio rerio*), LC50, 96 h, mg/L): 130** [5, 6]<br>**Daphnia (*Daphnia magna*, EC50, 48 h, mg/L): 365** [5, 6]<br>**Algae (*Pseudokirchneriella subcapitata,* ErC50, 72 h, mg/L): 1240** [5, 6]<br><br>Chronic Bacteria (activated sludge, NOEC, 13 d, mg/L): 72 [5]<br>Chronic Daphnia (*Daphnia magna*, NOEC, 21 d, mg/L): ≥100 [5, 6]	Readily biodegradable: 100% in 14 d (OECD 301C); BOD: 86 mg/g<br><br>No bioaccumulation (not persistent)	**Fish: 1**<br>**Daphnia: 1**<br>**Algae: 2**<br>Total points: 4	Corrosive to metals: 1<br>Skin Corrosion: 1A<br>Eye Damage: 1 (serious)<br>Acute Toxicity (oral): 4<br>Acute Toxicity (inhalation): 3<br>Specific target organ toxicity (single exposure & repeated exposure: respiratory system): 1 & 2<br>Corrosive to the respiratory tract<br>Flammable Liquid: 3<br>**Acute toxicity (oral, rat, LD50, mg/kg): 730** [5, 6]<br>**Acute toxicity (inhalation, rat, LC50, 4 h, mg/L): 7.85** [5, 6]	**Oral: 5**<br>**Inhalation: 2**<br>Total points: 7<br>Dermal: NA	[174-177]
**Lactic acid**	**Fish (Rainbow trout: *Oncorhynchus mykiss*, LC50, 96 h, mg/L): 130** [7]<br>**Daphnia (*Daphnia magna*, EC50, 48 h, mg/L): 130** [6]	Readily biodegradable: 80% in 28 d (OECD 301); BOD: 86 mg/g	**Fish: 1**<br>**Daphnia: 2**<br>**Algae: 1**<br>Total points: 4	Skin Corrosion: 1C<br>Eye Damage: 1<br>Corrosive to the respiratory tract<br>**Acute toxicity (oral, rat, LD50): mg/kg): 3543** [4], 4936 [4]<br>**Acute toxicity (inhalation, rat, LC50, 4 h, mg/L): 7.94** [6]	**Oral: 2**<br>**Inhalation: 1**<br>Total points: 3<br>Dermal: 2	[178-180]

Chemical	Key aquatic endpoints [1]	Biodegradability	Environmental toxicity severity (penalty point/rank) [2]	Key human hazard classes [3] and Toxicity endpoints derived from animal studies [1]	Animal toxicity severity (penalty point/rank) [2]	Reference
	**Algae (*Pseudokirchneriella subcapitata*, ErC50, 72 h, mg/L): 3500** [6]<br>Chronic Fish (*Oreochromus mossambica,* LOEC, 90 d, mg/L): 2.18 [7]<br>Bacteria (EC50, activated sludge, 3 h, mg/L): > 88.2 mg/L [6]	No bioaccumulation (not persistent)		Acute toxicity (dermal, rabbit, LD50, mg/kg): 2000 [4]		
**Citric acid**	Fish (Golden orfe: *Leuciscus idus*, LC50, 96 h, mg/L): 440–760 [5, 6]<br>**Fish (Rainbow trout, Oncorhynchus *mykiss*, LC50, 96 h, mg/L): > 100** [5, 6]<br>**Fish (Bluegill, *Lepomis macrochirus*, LC50, 96 h, mg/L) = 1516** [4]<br>**Fish (Fathead minnow: *Lepomis macrochirus*, LC50, 96 h, mg/L): > 100** [5]<br>**Daphnia: (*Daphnia magna,* EC50, 48 h,** 24 h un-neutralized, 24 h neutralized, **mg/L): 34** [5, 6], 85 [6], 1535 [6]<br>**Algae (*Desmodesmus subspicatus*, EC50, 72 h, mg/L): 1.9** [5, 6]<br>Algae (*Scenedesmus quadricauda,* NOEC, 72 h, 8	Readily biodegradable: 97% in 28 d (OECD 301B); BOD: 526 mg/g<br><br>No bioaccumulation (not persistent)	**Fish: 2**<br>**Daphnia: 4**<br>**Algae: 4**<br>Total points: 10	Skin corrosion: 2<br>Eye damage: 2<br>Specific target organ toxicity (single exposure, respiratory irritation): 3<br>**Acute toxicity (oral, rat, LD50, mg/kg): ≥ 5400** [5, 6], 11700 [6]<br>**Acute toxicity (inhalation, rat, LC50, 4 h, mg/L): 1.70** [6]<br>Acute toxicity (dermal, rat, LD50, mg/kg): > 2000 [5, 6]	**Oral: 1**<br>**Inhalation: 4**<br>Total points: 5<br>Dermal: 2	[168,175,181-184]

Chemical	Key aquatic endpoints [1]	Biodegradability	Environmental toxicity severity (penalty point/rank) [2]	Key human hazard classes [3] and Toxicity endpoints derived from animal studies [1]	Animal toxicity severity (penalty point/rank) [2]	Reference
	h, 8 d, mg/L): 1.4 [5, 6], 425 [5, 6], 640 [5, 6]<br>Bacteria (*Photobacterium Phosphoreum,* EC50, 15 min, 16 h, mg/L): 14 [7], > 10000 [6]					
			**Inorganic acids**			
**Hydrochloric acid**	Acute aquatic toxicity: 1 (very toxic)<br><br>Fish (Mosquitoo fish: *Gambusia affinis*, LC50, 96 h, mg/L): 282 [7]<br>**Fish (Bluegill: *Lepomis macrochirus*, LC50, 96 h, mg/L): 20.5** [5, 6]<br>**Daphnia (*Daphnia magna,* EC50, 48 h,** 76 h**, mg/L): 0.45** [6]<br>**Algae (*Chlorella vulgaris*, EC50, 72 h, mg/L): 0.73** [5, 6]<br>Bacteria (activated sludge, EC50, 3 h, mg/L): 0.23 [5,6]	Not applicable for inorganic acids (NA)	**Fish: 4**<br>**Daphnia: 5**<br>**Algae: 5**<br>Total points: 14	Metal corrosion: 1<br>Skin corrosion: 1A<br>Eye damage: 1<br>Acute Toxicity (oral): 4<br>Acute toxicity (inhalation): 1<br>Specific target organ toxicity (single &repeated exposure, respiratory irritation, teeth): 1<br>**Acute toxicity (oral, rat, LC50, mg/kg): 238-277** [7]<br>**Acute toxicity (inhalation, rat, 4 h,** 1 h**, mg/L): 2.10** [7], 1.68 [7]<br>Acute toxicity (dermal, LD50, rabbit, mg/kg): > 5010 [7]	**Oral: 6**<br>**Inhalation: 3**<br>Total points: 9<br>Dermal: 1	[185-188]
**Sulfuric acid**	**Fish (Zebra fish: *Brachydanio rerio,* LC50, 96 h, mg/L): > 500** [7]<br>**Fish (Bluegill: *Lepomis macrochirus*, LC50, 96 h, mg/L): 16-28** [7]<br>**Daphnia (*Daphnia magna*, EC50, 48 h,** 24 h, **mg/L): > 100** [5, 6], 29 [7]	NA	**Fish: 5**<br>**Daphnia: 3**<br>**Algae: 3**<br>Total points: 11	Metal corrosion: 1<br>Skin corrosion: 1A<br>Eye damage: 1<br>Acute toxicity (inhalation): 2<br>Specific target organ toxicity (single & repeated exposure, respiratory irritation): 1<br>**Acute toxicity (oral, rat, LD50, mg/kg): 2140** [5]	**Oral: 3**<br>**Inhalation: 6**<br>Total points: 9<br>Dermal: NA	[189-193]

Chemical	Key aquatic endpoints [1]	Biodegradability	Environmental toxicity severity (penalty point/rank) [2]	Key human hazard classes [3] and Toxicity endpoints derived from animal studies [1]	Animal toxicity severity (penalty point/rank) [2]	Reference
	**Algae (*Desmodesmus subspicatus*, ErC50, 72 h, mg/L): > 100** [5, 6]<br>Chronic Fish (NOEC, 65 d, mg/L): 0.025 [5]			**Acute toxicity (inhalation, rat,** 1 h**, 4 h, mg/L):** 0.085-0.103 [7], **0.375** [7]<br>Acute toxicity (dermal, rabbit): extremely corrosive		
**Phosphoric acid**	Acute aquatic toxicity: 3<br><br>**Fish (Rainbow trout: *Oncorhynchus mykiss*, LC50, 96 h, mg/L): 87** [7]<br>**Fish (Bluegill: *Lepomis macrochirus*, LC50, 96 h, mg/L): 60** [7]<br>**Daphnia (*Daphnia,magna*, EC50, 48 h, mg/L): > 100** [6]<br>**Algae (*Desmodesmus subspicatus*, ErC50, 72 h, mg/L): > 100** [6]<br>Algae (NOEC, *Desmodesmus subspicatus*, 72 h): 100 [6]<br>Bacteria (activated sludge, EC50, 3 h, mg/L): > 1000 [6]<br>Bacteria (activated sludge, NOEC, 3 h, mg/L): 1000 [7]	NA	**Fish: 3**<br>**Daphnia: 3**<br>**Algae: 3**<br>Total points: 9	Metal corrosion: 1<br>Skin corrosion: 1B<br>Eye damage: 1<br>Specific target organ toxicity (single exposure, respiratory irritation): 1<br>Acute toxicity (oral): 4, Harmful (severe burn damage), > 300 [5] mg/kg<br>Acute toxicity (dermal): 4<br>Acute toxicity (inhalation): 3<br>**Acute toxicity (oral, rat, LD50, mg/kg): 2000** [6]<br>**Acute toxicity (inhalation, rat, 4 h, mg/L): 0.9615** [7]<br>Acute toxicity (dermal, rabbit, LD50, mg/kg): 1071 [7]	**Oral: 4**<br>**Inhalation: 5**<br>Total points: 9<br>Dermal: 3	[194-198]

1: Data obtained from MSDS according to available sources for toxicological endpoint values. When multiple values were available from various reliable sources (e.g., ECHA, ECOTOX, OECD-based), the lowest value was selected to ensure a conservative approach. If both reliable and unverified (source not specified) data were available, the value from the reliable source was prioritized, even if the unverified source reported a more conservative figure. Values from unverified sources were used only when no reliable data were available; 2: The lower the rank/penalty point, the lower the toxicity (larger points = more toxicity); 3: Hazard classifications summarize the representative SDS sources used in this review and concern handling hazards. The numbers are MSDS-derived hazard categories, the lower the category number, the more toxic/hazardous the chemical is; 4: ECOTOX: U.S. EPA (U.S. Environmental Protection Agency Ecotoxicology Database): a collection of peer-reviewed ecotoxicological test results conducted according to internationally recognized (e.g., OECD or ASTM) test methods; 5: ECHA

(European Chemicals Agency (EU REACH database) of OECD-based studies; 6: OECD (The Organization for Economic Co-operation and Development) standard test guideline; 7: Source from which the toxicological values are retrieved, not provided; LC50: Lethal Concentration 50%; EC50: Effective Concentration 50%; LD50: Lethal Dose, 50%; IC50: Inhibitory Concentration 50%; ErC50: Effective concentration (E_rC_{50}) that reduces the rate of growth by 50%; NOEC: No-Observed-Effect Concentration.

Table S11. Acute toxicity of acids to different fish species and relative toxicity ranking.

Acid	Fish species	Acute toxicity (mg/L)	Endpoint	Toxicity Rank (severity) [1] within the same species (Intra-species)	Interpretation
Formic acid	Zebrafish	130	LC50	2 out of 2	More toxic than sulfuric acid
Lactic acid	Rainbow trout	130	LC50	1 out of 3	Lowest toxicity in Rainbow trout
Citric acid	Goldfish	400	LC50	Not comparable	No internal comparison
	Rainbow trout	> 100	LC50	2 out of 3	Intermediate toxicity; more toxic than lactic acid, less toxic than phosphoric acid
	Bluegill	1516	LC50	1 out of 4	Lowest toxicity in Bluegill
	Fathead minnow	100	LC50	Not comparable	No internal comparison
Hydrochloric acid	Mosquito fish	282	LC50	Not comparable	No internal comparison
	Bluegill	20.5	LC50	3 out of 4	Second most toxic in Bluegill; more toxic than phosphoric acid and citric acid, less toxic than sulfuric acid
Sulfuric acid	Zebrafish	500	LC50	1 out of 2	Less toxic than formic acid
	Bluegill	16-28	LC50	4 out of 4	Highest toxicity in Bluegill; more toxic than hydrochloric acid, phosphoric acid, and citric acid
Phosphoric acid	Rainbow trout	87	LC50	3 out of 3	Highest toxicity in Rainbow trout; more toxic than citric acid and lactic acid
	Bluegill	60	LC50	2 out of 4	Intermediate toxicity; more toxic than citric acid, and less toxic than hydrochloric acid and sulfuric acid

1: The lower the rank, the lower the toxicity (larger values = more toxicity).

Table S12. Results of ecotoxicity tests and regulatory guidelines for inorganic ions derived from alkaline and bleaching chemicals. Comparable values are shown in the same color. Data were obtained from references 55 to 63 and the related links provided below the Table.

**Ecotoxicity tests/guideline values**	**Ions**							**Severity order and Rank** [1]		
	**Na^+**	**K^+**	**Chlorite (ClO_2^-)**	**Chlorine dioxide (ClO_2)**	**Chlorate (ClO_3^-)**	**Chlorine (Cl_2)**	**Free Chlorine (ClO^- /HClO)**	**Acute/ short-term benchmark**	**Chronic/ protective benchmarks**	**Drinking water guidelines**
Guideline value for drinking water taste [2]	≥ 200							K^+ (3–6 times more) > Na^+		
Acute toxicity values (48 h LC_{50}) for Ceriodaphnia dubia (mg/L) across different background waters [3]	907-2420	153-752						Na^+: 1<br>K^+: 2		
MCLG [4] in drinking water (mg/L) [4; 5]			0.8	0.8		4				MCLG<br>ClO_2^- = ClO_2 > Cl_2<br>Cl_2: 1<br>ClO_2^-: 2<br>ClO_2: 2<br>NaCLO: 1<br>$NaClO_2$: 2
Acute toxicity for freshwater species: fish & invertebrates (Daphnidae: 0.022; Chironomidae; 90.67) (mg/L) [6]			5.79-209 & 0.022-90.67							
Acute oral toxicity (LD 50, rats, mg/L) [7]			105-165	292	1200			ClO_2^- > ClO_2 > ClO_3^-<br>ClO_3^-: 1<br>ClO_2: 2<br>ClO_2^-: 3<br>NaCLO: 1<br>$NaClO_2$: 2		
NOAEL[8] (for stomach lesions and increases in spleen and adrenal weights in rats, mg/kg/day) [7]			7.4							
NOAEL (for hematology in rats, mg/kg/day) [7]			1						ClO_2^- > ClO_2 >ClO_3^-<br>ClO_3^-: 1<br>ClO_2: 2<br>ClO_2^-: 3<br>NaCLO: 1<br>$NaClO_2$: 2	
NOAEL (for hematology in Sprague-Dawley rats, mg/kg/day) [7]					100					
NOAEL (for Thyroid & Pituitary lesions in Sprague-Dawley rats, mg/kg/day) [7]					30					

Ecotoxicity tests/guideline values	Ions							Severity order and Rank [1]		
	Na^+	K^+	Chlorite (ClO_2^-)	Chlorine dioxide (ClO_2)	Chlorate (ClO_3^-)	Chlorine (Cl_2)	Free Chlorine (ClO^- /HClO)	Acute/ short-term benchmark	Chronic/ protective benchmarks	Drinking water guidelines
NOAEL (for Serum chemistry, blood count and urinalysis parameters in humans, mg/kg/day) [7]			0.034	0.34	0.034				ClO_2^- = ClO_3^- > ClO_2	*MAC:*<br>ClO_2 >ClO_2^- > ClO_3^-
LOAEL[9] (for Nasal histopathology in Sprague-Dawley rats, mg/kg/day) [7]				2					ClO_2: 1<br>ClO_3^-: 2<br>ClO_2^-: 2	ClO_3^-: 1<br>ClO_2^-: 2<br>ClO_2: 3
MAC [10] for drinking water (mg/L) [7]			1.083	0.4 [10]	1.12				$NaClO_2$ = NaCLO	NaCLO: 1<br>$NaClO_2$: 2
LOAEL (for erythrocyte membrane damage in rats, mg/kg/day) [11]			19							
NOAEL (for blood parameters in mice, mg/kg/day) [11]				15						
Taste and odor threshold (mg/L) [11]				0.2-0.4						
EPA guideline values for drinking water (mg/L) [11]			0.7		0.7					EPA:<br>ClO_2^- = ClO_3^-
Health-based values for drinking water (mg/L) [11]					0.3					
LOAEL (for follicular cell hyperplasia in rats, mg/kg/day) [11]					75					
Acute toxicity (LC50, 96h, fish (larvae and adults, mg/L) [12]			106 & 149	2.2 & 8.3				ClO^- /HClO > ClO_2 >> ClO_2^-		
Chronic toxicity (MATC [13]), fish, mg/L) [12]			3.3	0.21				ClO_2^-: 1<br>ClO_2: 2	ClO^- /HClO > ClO_2 > ClO_2^-	
Acute toxicity (LC50, 30 min, 60 min & 4 d, *Duphnia mugnu*) (mg/L) [14]							0.063, 0.097 & 0.017	ClO^- /HClO: 3<br>$NaClO_2$: 1<br>NaCLO: 2	ClO_2^-: 1<br>ClO_2: 2<br>ClO^-/HClO:3	
Survival (24 h, *Cerioduphniu dubiu*) at 0.1 mg/L ion concentration with food [14]							30%/100%	ClO^- > HClO	$NaClO_2$: 1<br>NaCLO: 2	
Survival (24 h, *Cerioduphniu dubiu*) at 0.02 & 0.04 mg/L ion concentration without food [14]							100% & 80% / 100% & 30%	HClO > ClO^-		

Ecotoxicity tests/guideline values	Ions							Severity order and Rank [1]		
	Na^+	K^+	Chlorite (ClO_2^-)	Chlorine dioxide (ClO_2)	Chlorate (ClO_3^-)	Chlorine (Cl_2)	Free Chlorine (ClO^- /HClO)	Acute/ short-term benchmark	Chronic/ protective benchmarks	Drinking water guidelines
Acute toxicity (LC50, *Cerioduphniu dubiu*, fed, unfed, continues) (mg/L) [14]							0.08, 0.048, 0.006/ 0.14, 0.035, 0.005	ClO^- > HClO<br>HClO > ClO^-<br>HClO > ClO^-		
Acute toxicity (LC50, 24-96h, freshwater fish, mg/L) [15] (sensitive sp. *Oncorhynchus mykiss*)							0.07-0.840 (0.014)			
Acute (LC50, 48-96h) & chronic (NOEC 16, 7d) toxicity (marine fish, mg/L) [15]							0.128-0.250 & 0.02-0.087			

1: The lower the rank, the lower the toxicity (larger values = more toxicity); 2: [199]; 3: [200]; 4: MCLG (Maximum Contaminant Level Goal), data retrieved from the EPA website [201]; 5: [202]; 6: [203]; 7: [204]; 8: NOAEL (No Observed Adverse Effect Level), the highest dose or exposure level of a substance at which no statistically significant, harmful (adverse) health effects are observed in a tested population; 9 LOAEL (Lowest Observed Adverse Effect Level), the lowest concentration or dose of a substance that causes a harmful effect in people or animals; 10: MAC (Maximum Acceptable Concentration), MAC has not been proposed for chlorine dioxide because of its rapid reduction to chlorite (and, to a lesser extent, chlorate), the taste and odor threshold for chlorine dioxide is 0.4 mg/L (lower than the MACs derived for chlorite and chlorate); 11: [205]; 12: [206]; 13: MATC (Maximum-Acceptable-Toxicant-Concentration); 14: [207]; 15: [208]; 16: NOEC (No-Observed-Effect Concentration).

Table S13. Results of ecotoxicity tests and regulatory guidelines for inorganic ions derived from mineral acids. Comparable values are shown in the same color. Data were obtained from references 64 to 69 provided below the Table.

Ecotoxicity tests/guideline values	Ions			Severity order and Rank [4]		
	Chloride	Sulfate	Phosphate	Acute/short-term benchmark	Chronic/protective benchmarks	Drinking water guidelines
Final acute [1] aquatic toxicity (mg/L) (6)	640	1200				
Final chronic [2] aquatic toxicity (mg/L) (6)	150	370		Phosphate > Chloride > Sulfate	Chloride > Sulfate	Chloride > Sulfate > Phosphate
Long-term (L) & short-term (S) exposure values for the protection of aquatic life (mg/L) (7)	120 (L) & 640 (S)	-				
Acute toxicity (EC50) for aquatic organisms (mg/L): Fatmucket, Rainbow mussel, & Amphipod (8)	-	2071, 2064, & 2689	-			
Ecotoxicity tests (acute) on Fish (LC50), Daphnia (EC50), and Algae (LC50) in mg/L (9)	-	-	> 100	Sulfate: 1<br>Chloride: 2<br>Phosphate: 3	Sulfate: 1<br>Chloride: 2<br>Phosphate: NA	Sulfate: 2<br>Chloride: 3<br>Phosphate: 1
Ecotoxicity tests (acute) on Fish (LC50), Daphnia (EC50), and Algae (LC50) in mg/L (from sodium phosphate (10))			178, 1085-1402, 158			
Qatar General Electricity and Water Corporation (KAHRAMAA) Requirements for Water Quality in Distribution System (11)	< 80	50	NA[3]			
Taste threshold (mg/mL) (11)	< 250	250 to 1000 [5] (usually < 250)	-			
WHO drinking water specifications (12)	200-300	There is no health-based guideline value [5]	-			

1: The concentration above which short-term exposure can cause immediate or serious harm to aquatic life; 2: The maximum concentration that marine life can safely tolerate over more extended periods (e.g., 4-day average); 3: There are no recommended levels for phosphate in the international drinking water guidelines. A low level of 0.01 is usually found in desalinated water; 4: The lower the rank/penalty point, the lower the toxicity (larger points = more toxicity); 5: Drinking water guideline values indicate more concern toward chloride than sulfate, while phosphate is not a regulated ion in drinking water (least concern); 6: [209]; 7: [210]; 8: [211]; 9: [212]; 10: [213]; 11: [214]; 12: [215].

Table S14. Summary of the chemical penalty points listed in Tables S7 to S13 and the sum of the total penalty points used for the overall ranking of the chemicals for environmental friendliness from a waste perspective.

Chemicals	Post-use environmental burden (Table S7)	Environmental toxicity severity (Fish) (Tables S8/S9/S10)	Environmental toxicity severity (Daphnia) (Tables S8/S9/S10)	Environmental toxicity severity (Algae) (Tables S9/S10)	Animal toxicity severity (Oral) (Tables S8/S9/S10)	Animal toxicity severity (Inhalation) (Tables S9/S10)	Animal toxicity severity (Dermal) (Table S9)	Inorganic ions ecotoxicity (Acute) (Table S12/S13)	Inorganic ions ecotoxicity (Chronic) (Table S12)	Inorganic ions (Water quality guidelines) (Table S12/S13)	Sum of the penalty points
**NaOH**	4	1	2	-	2	-	-	1	-	-	10
**KOH**	2	2	1	-	1	-	-	2	-	-	8
**$NaClO_2$**	4	1	2	1	3	3	3	1 & 2	1 & 2	2 & 2	27
**NaClO**	5	3	3	3	1	1	1	2 & 1	2 & 1	1 & 1	25
**H_2O_2**	1	2	1	2	2	2	2	-	-	-	12
**Formic acid**	1	1	1	2	5	2	-	-	-	-	12
**Lactic acid**	1	1	2	1	2	1	-	-	-	-	8
**Citric acid**	3	2	4	4	1	4	-	-	-	-	18
**HCl**	4	4	5	5	6	3	-	2	-	3	32
**H_2SO_4**	5	5	3	3	3	6	-	1	-	2	28
**H_3PO_4**	6	3	3	3	4	5	-	3	-	1	28

References

1. Poling, B.E.; Thomson, G.H.; Friend, D.G.; Rowley, R.L.; Wilding, W.V. *Perry's Chemical Engineers' Handbook*; McGraw-Hill Companies, Inc.: New York, NY, 2008.
2. Haynes, W.M. *CRC handbook of chemistry and physics*; CRC press: 2016.
3. Żarska, M.; Dzida, M.; Apelblat, A. Surface tensions and densities of concentrated aqueous solutions of citric acid. *J. Mol. Liq.* **2017**, *228*, 91-95, doi:10.1016/j.molliq.2016.07.019.
4. Mini Industry. Sodium hypochlorite solution density. Available online: https://www.miniindustry.com/d/sodium-hypochlorite-solution-density-naocl (accessed on 03 Dec 2025).
5. Occidenta Chemical Corporation OxyChem. *Sodium chlorite – solutions from dry product (Metric Units)*; OxyChem Basic Chemicals – Wichita Technical Service Department: Wichita, KS, USA, 2023.
6. Dormann, C.F.; Elith, J.; Bacher, S.; Buchmann, C.; Carl, G.; Carré, G.; Marquéz, J.R.G.; Gruber, B.; Lafourcade, B.; Leitão, P.J. Collinearity: a review of methods to deal with it and a simulation study evaluating their performance. *Ecography* **2013**, *36*, 27-46.
7. Fraley, C.; Raftery, A. Model-based clustering, discriminant analysis, and density estimation. *J. Am. Stat. Assoc.* **2002**, *97*, 611-631, doi:10.1198/016214502760047131.
8. Sigma Aldrich. Sigma-Aldrich product and safety information. Available online: https://www.sigmaaldrich.com/ (accessed on 20 Nov 2025).
9. Fisher Scientific. Fisher Scientific product and safety information. Available online: https://www.fishersci.com/ (accessed on 20 Nov 2025).
10. Wahib, S.A.; Da'na, D.A.; Al-Ghouti, M.A. Insight into the extraction and characterization of cellulose nanocrystals from date pits. *Arab. J. Chem.* **2022**, *15*, 103650, doi:10.1016/j.arabjc.2021.103650.
11. Mishra, S.; Prabhakar, B.; Kharkar, P.S.; Pethe, A.M. Banana peel waste: An emerging cellulosic material to extract nanocrystalline cellulose. *Acs Omega* **2022**, *8*, 1140-1145, doi:10.1021/acsomega.2c06571.
12. Beyan, S.M.; Amibo, T.A.; Prabhu, S.V.; Ayalew, A.G. Production of nanocellulose crystal derived from enset fiber using acid hydrolysis coupled with ultrasonication, isolation, statistical modeling, optimization, and characterizations. *J. Nanomater.* **2021**, *2021*, 7492532, doi:10.1155/2021/7492532.
13. George, T.S.; Muhammadaly, S.A.; Parambath Kanoth, B.; Joseph, T.; Chemmarickal Dominic, M.D.; George, N.; Balachandrakurupp, V.; John, H. Isolation of high crystalline nanocellulose from Mimosa pudica plant fibres with potential in packaging applications. *Packag. Technol. Sci.* **2022**, *35*, 163-174, doi:10.1002/pts.2617.
14. Bahloul, A.; Semlali, F.-Z.; Oumam, M.; Hannache, H.; Kassab, Z.; El Achaby, M. Starch bio-nanocomposites based on phosphorylated and sulphated cellulose nanocrystals extracted from pepper plant residue: effect of surface functionality on property improvements. *Cellulose* **2023**, *30*, 5051-5070, doi:10.1007/s10570-023-05199-4.
15. Kassab, Z.; Kassem, I.; Hannache, H.; Bouhfid, R.; Qaiss, A.E.K.; El Achaby, M. Tomato plant residue as new renewable source for cellulose production: extraction of cellulose nanocrystals with different surface functionalities. *Cellulose* **2020**, *27*, 4287-4303, doi:10.1007/s10570-020-03097-7.

16. Perumal, A.B.; Sellamuthu, P.S.; Nambiar, R.B.; Sadiku, E.R. Development of polyvinyl alcohol/chitosan bio-nanocomposite films reinforced with cellulose nanocrystals isolated from rice straw. *Appl. Surf. Sci.* **2018**, *449*, 591-602, doi:10.1016/j.apsusc.2018.01.022.
17. Perumal, A.B.; Nambiar, R.B.; Sellamuthu, P.S.; Sadiku, E.R.; Li, X.; He, Y. Extraction of cellulose nanocrystals from areca waste and its application in eco-friendly biocomposite film. *Chemosphere* **2022**, *287*, 132084, doi:10.1016/j.chemosphere.2021.132084.
18. Raza, M.; Abu-Jdayil, B.; Banat, F.; Al-Marzouqi, A.H. Isolation and characterization of cellulose nanocrystals from date palm waste. *ACS omega* **2022**, *7*, 25366-25379.
19. Kassab, Z.; Syafri, E.; Tamraoui, Y.; Hannache, H.; Qaiss, A.E.K.; El Achaby, M. Characteristics of sulfated and carboxylated cellulose nanocrystals extracted from Juncus plant stems. *International Journal of Biological Macromolecules* **2020**, *154*, 1419-1425.
20. Akinjokun, A.I.; Petrik, L.F.; Ogunfowokan, A.O.; Ajao, J.; Ojumu, T.V. Isolation and characterization of nanocrystalline cellulose from cocoa pod husk (CPH) biomass wastes. *Heliyon* **2021**, *7*.
21. Hemida, M.H.; Moustafa, H.; Mehanny, S.; Morsy, M.; Abd EL Rahman, E.N.; Ibrahim, M.M. Valorization of Eichhornia crassipes for the production of cellulose nanocrystals further investigation of plethoric biobased resource. *Sci. Rep.* **2024**, *14*, 12387, doi:10.1038/s41598-024-62159-z.
22. Lu, S.; Ma, T.; Hu, X.; Zhao, J.; Liao, X.; Song, Y.; Hu, X. Facile extraction and characterization of cellulose nanocrystals from agricultural waste sugarcane straw. *J. Sci. Food Agric.* **2022**, *102*, 312-321, doi:10.1002/jsfa.11360.
23. Abdul Rahman, N.H.; Chieng, B.W.; Ibrahim, N.A.; Abdul Rahman, N. Extraction and characterization of cellulose nanocrystals from tea leaf waste fibers. *Polymers* **2017**, *9*, 588, doi:10.3390/polym9110588.
24. Thipchai, P.; Punyodom, W.; Jantanasakulwong, K.; Thanakkasaranee, S.; Sringarm, K.; Panyathip, R.; Tanadchangsaeng, N.; Worajittiphon, P.; Rachtanapun, P. Property investigation of nanocellulose I and II from different non-wood fibers using chemical acid hydrolysis method. *Ind. Crops Prod.* **2024**, *222*, 119534, doi:10.1016/j.indcrop.2024.119534.
25. Mahardika, M.; Abral, H.; Kasim, A.; Arief, S.; Asrofi, M. Production of nanocellulose from pineapple leaf fibers via high-shear homogenization and ultrasonication. *Fibers* **2018**, *6*, 28, doi:10.3390/fib6020028.
26. Fortunati, E.; Luzi, F.; Puglia, D.; Petrucci, R.; Kenny, J.M.; Torre, L. Processing of PLA nanocomposites with cellulose nanocrystals extracted from Posidonia oceanica waste: Innovative reuse of coastal plant. *Ind. Crops Prod.* **2015**, *67*, 439-447, doi:10.1016/j.indcrop.2015.01.075.
27. Nagarajan, K.J.; Sanjay, M.R.; Basha, K.S.; Raghav, G.R.; Kumar, R.A.; Siengchin, S.; Rajan, B.S.; Nath, P.S.; Khan, A. Extraction of cellulose nanocrystals from red banana peduncle agro-waste and application in environmentally friendly biocomposite film. *Polym. Compos.* **2022**, *43*, 4942-4958, doi:10.1002/pc.26755.
28. Schiavi, D.; Francesconi, S.; Taddei, A.R.; Fortunati, E.; Balestra, G.M. Exploring cellulose nanocrystals obtained from olive tree wastes as sustainable crop protection tool against bacterial diseases. *Sci. Rep.* **2022**, *12*, 6149, doi:10.1038/s41598-022-10225-9.
29. Verma, C.; Chhajed, M.; Gupta, P.; Roy, S.; Maji, P.K. Isolation of cellulose nanocrystals from different waste bio-mass collating their liquid crystal ordering with morphological

exploration. *Int. J. Biol. Macromol.* **2021**, *175*, 242-253, doi:10.1016/j.ijbiomac.2021.02.038.

30. Rabby, M.M.R.; Rahman, M.M.; Ghos, B.C.; Gafur, M.A.; Al-Amin, M.; Dewan, S.; Alam, M.A.; Hossain, M.I. Production of CNC from agro-waste biomass (maize shells) as a potential reinforcement in bio-nanocomposites: extraction, modification, and characterization study. *Carbohydr. Polym. Technol. Appl.* **2025**, *9*, 100671, doi:10.1016/j.carpta.2025.100671.
31. Rahman, M.M.; Pk, M.E.H.; Waliullah, M.; Hossain, M.I.; Maniruzzaman, M.; Ghos, B.C. Production of cellulose nanocrystals from the waste banana (M. Oranta) tree rachis fiber as a reinforcement to fabricate useful bionanocomposite. *Carbohydr. Polym. Technol. Appl.* **2024**, *8*, 100607, doi:10.1016/j.carpta.2024.100607.
32. Rahman, M.M.; Hossain, M.I.; Hassan, M.M.; Ghos, B.C.; Rahman, M.S.; Gafur, M.A.; Alam, M.A.; Al Zuhanee, M.K. Cellulose nanocrystal (CNC) from okra plant (Abelmoschus esculentus L.) stalks as a reinforcement in bionanocomposite fabrication: Extraction, processing, and characterization study. *Carbohydr. Polym. Technol. Appl.* **2024**, *8*, 100581, doi:10.1016/j.carpta.2024.100581.
33. Gabriel, T.; Belete, A.; Hause, G.; Neubert, R.H.; Gebre-Mariam, T. Isolation and characterization of cellulose nanocrystals from different lignocellulosic residues: a comparative study. *Journal of Polymers and the Environment* **2021**, *29*, 2964-2977.
34. Hemmati, F.; Jafari, S.M.; Taheri, R.A. Optimization of homogenization-sonication technique for the production of cellulose nanocrystals from cotton linter. *Int. J. Biol. Macromol.* **2019**, *137*, 374-381, doi:10.1016/j.ijbiomac.2019.06.241.
35. Prado, K.S.; Spinacé, M.A. Isolation and characterization of cellulose nanocrystals from pineapple crown waste and their potential uses. *Int. J. Biol. Macromol.* **2019**, *122*, 410-416, doi:10.1016/j.ijbiomac.2018.10.187.
36. Valencia, L.; Arumughan, V.; Jalvo, B.; Maria, H.J.; Thomas, S.; Mathew, A.P. Nanolignocellulose extracted from environmentally undesired Prosopis juliflora. *ACS omega* **2019**, *4*, 4330-4338, doi:10.1021/acsomega.8b02685.
37. Nang An, V.; Chi Nhan, H.T.; Tap, T.D.; Van, T.T.T.; Van Viet, P.; Van Hieu, L. Extraction of high crystalline nanocellulose from biorenewable sources of Vietnamese agricultural wastes. *J. Polym. Environ.* **2020**, *28*, 1465-1474, doi:10.1007/s10924-020-01695-x.
38. Jiang, F.; Hsieh, Y.-L. Cellulose nanocrystal isolation from tomato peels and assembled nanofibers. *Carbohydr. Polym.* **2015**, *122*, 60-68, doi:10.1016/j.carbpol.2014.12.064.
39. Gabriel, T.; Wondu, K.; Dilebo, J. Valorization of khat (Catha edulis) waste for the production of cellulose fibers and nanocrystals. *PLoS One* **2021**, *16*, e0246794, doi:10.1371/journal.pone.0246794.
40. Akatan, K.; Kabdrakhmanova, S.; Kuanyshbekov, T.; Ibraeva, Z.; Battalova, A.; Joshy, K.; Thomas, S. Highly-efficient isolation of microcrystalline cellulose and nanocellulose from sunflower seed waste via environmentally benign method. *Cellulose* **2022**, *29*, 3787-3802, doi:10.1021/acsomega.3c00295.
41. Abitbol, T.; Kubat, M.; Brannvall, E.; Kotov, N.; Johnson, C.M.; Nizamov, R.; Nyberg, M.; Miettunen, K.; Nordgren, N.; Stevanic, J.S.; Guerreiro, M.P. Isolation of Mixed Compositions of Cellulose Nanocrystals, Microcrystalline Cellulose, and Lignin Nanoparticles from Wood Pulps. *ACS Omega* **2023**, *8*, 21474-21484, doi:10.1021/acsomega.3c00295.

42. Alifa, T.; Aminatou, H.; Said, O.; Tchatchueng, J.-B. Optimization of cellulose nanocrystal isolation from ayous sawdust using response surface methodology. *J. Mater. Sci. Chem. Eng.* **2024**, *12*, 36-55, doi:10.4236/msce.2024.121004.
43. Arbelaiz, A.; Orue, A. Walnut shells as cellulose nanocrystal source: isolation procedure and properties characterization (vol 14, pg 17579, 2023). *Biomass Convers. Biorefin.* **2024**, *14*, 30857-30857, doi:10.1007/s13399-023-03996-2.
44. Chawalitsakunchai, W.; Dittanet, P.; Loykulnunt, S.; Tanpichai, S.; Prapainainar, P. Extraction of nanocellulose from pineapple leaves by acid-hydrolysis and pressurized acid hydrolysis for reinforcement in natural rubber composites. In Proceedings of the IOP Conference Series: Materials Science and Engineering, 2019; p. 012019.
45. Chen, L.; Wang, Q.; Hirth, K.; Baez, C.; Agarwal, U.P.; Zhu, J. Tailoring the yield and characteristics of wood cellulose nanocrystals (CNC) using concentrated acid hydrolysis. *Cellulose* **2015**, *22*, 1753-1762, doi:10.1007/s10570-015-0615-1.
46. Dewan, S.; Rahman, M.M.; Hossain, M.I.; Ghos, B.C.; Rabby, M.M.R.; Gafur, M.A.; Al-Amin, M.; Alam, M.A. Isolation and characterization of CNC from waste maize cob available in Bangladesh as a potential candidate for the fabrication of multifunctional bio-nanocomposites: A new approach. *S. Afr. J. Chem. Eng.* **2025**, *51*, 287-301, doi:10.1016/j.sajce.2024.12.007.
47. Sartika, D.; Firmansyah, A.P.; Junais, I.; Arnata, I.W.; Fahma, F.; Firmanda, A. High yield production of nanocrystalline cellulose from corn cob through a chemical-mechanical treatment under mild conditions. *Int. J. Biol. Macromol.* **2023**, *240*, 124327, doi:10.1016/j.ijbiomac.2023.124327.
48. de Farias, D.T.; Labidi, J.; Pedrazzi, C.; Gatto, D.A.; de Cademartori, P.H.G.; Welter, C.A.; da Silva, G.T.; de Almeida, T.M. Acid-hydrolysis-assisted cellulose nanocrystal isolation from acacia mearnsii de wild. wood kraft pulp. *Polymers* **2024**, *16*, 3371.
49. Haddis, D.Z.; Chae, M.; Asomaning, J.; Bressler, D.C. Evaluation of steam explosion pretreatment on the cellulose nanocrystals (CNCs) yield from poplar wood. *Carbohydr. Polym.* **2024**, *323*, 121460, doi:10.1016/j.carbpol.2023.121460.
50. Kandhola, G.; Djioleu, A.; Rajan, K.; Labbé, N.; Sakon, J.; Carrier, D.J.; Kim, J.-W. Maximizing production of cellulose nanocrystals and nanofibers from pre-extracted loblolly pine kraft pulp: a response surface approach. *Bioresour. Bioprocess.* **2020**, *7*, 19, doi:10.1186/s40643-020-00302-0.
51. Kandhola, G.; Djioleu, A.; Rajan, K.; Batta-Mpouma, J.; Labbé, N.; Sakon, J.; Babst, B.A.; Ghosh, A.; Carrier, D.J.; Kim, J.-W. Impact of species-based wood feedstock variability on physicochemical properties of cellulose nanocrystals. *Cellulose* **2022**, *29*, 8213-8228, doi:10. 1007/ s10570- 022- 04762-9.
52. Lin, K.-H.; Enomae, T.; Chang, F.-C. Cellulose nanocrystal isolation from hardwood pulp using various hydrolysis conditions. *Molecules* **2019**, *24*, 3724, doi:10.3390/molecules24203724.
53. Çiçek Özkan, B.; Güner, M. Isolation, characterization, and comparison of nanocrystalline cellulose from solid wastes of horse chestnut and chestnut seed shell. *Cellulose* **2022**, *29*, 6629-6644, doi:10.1007/s10570-022-04682-8.
54. Pantamanatsopa, P.; Ariyawiriyanan, W.; Ekgasit, S. Production of cellulose nanocrystals suspension with high yields from water hyacinth. *J. Nat. Fibers* **2023**, *20*, 2134266, doi:10.1080/15440478.2022.2134266.

55. Song, K.; Zhu, X.; Zhu, W.; Li, X. Preparation and characterization of cellulose nanocrystal extracted from Calotropis procera biomass. *Bioresour. Bioprocess.* **2019**, *6*, 1-8, doi:10.1186/s40643-019-0279-z.
56. Dube, A.M. Isolation and characterization of cellulose nanocrystals from Ensete ventricosum pseudo-stem fiber using acid hydrolysis. *Biomass Convers. Biorefin.* **2025**, *15*, 30139-30154, doi:10.1007/s13399-022-02987-z.
57. Adel, A.M.; El-Shafei, A.M.; Ibrahim, A.A.; Al-Shemy, M.T. Chitosan/nanocrystalline cellulose biocomposites based on date palm (Phoenix dactylifera L.) sheath fibers. *J. Renew. Mater.* **2019**, *7*, 567-582, doi:10.32604/jrm.2019.00034.
58. Asghari, M.; Karimi Zarchi, A.A.; Taheri, R.A. Preparation and characterization nanocrystalline cellulose as a food additive to produce healthy biscuit cream. *Starch-Stärke* **2021**, *73*, 2000033, doi:10.1002/star.202000033.
59. Asmoro, N.W.; Hidayat, C.; Ariyanto, T.; Millati, R. Cellulose isolation from oil palm empty fruit bunch using different Pretreatment processes. *J. Appl. Sci. Eng.* **2023**, *26*, 1513-1520, doi:10.6180/jase.202311_26(11).0001.
60. Asmoro, N.W.; Hidayat, C.; Ariyanto, T.; Millati, R. Extraction of nanocrystalline cellulose from oil palm empty fruit bunches using sulfuric acid hydrolysis method. In Proceedings of the IOP Conf. Ser.: Earth Environ. Sci., 2025; p. 012022.
61. Brahma, R.; Ray, S. Optimization of extraction conditions for cellulose from jackfruit peel using RSM, its characterization and comparative studies to commercial cellulose. *Measurement: Food* **2024**, *13*, 100130, doi:10.1016/j.meafoo.2023.100130.
62. Barlianti, V.; Dahnum, D.; Hendarsyah, H.; Abimanyu, H. Effect of alkaline pretreatment on properties of lignocellulosic oil palm waste. *Procedia Chem.* **2015**, *16*, 195-201, doi:10.1016/j.proche.2015.12.036.
63. Beltramino, F.; Roncero, M.B.; Torres, A.L.; Vidal, T.; Valls, C. Optimization of sulfuric acid hydrolysis conditions for preparation of nanocrystalline cellulose from enzymatically pretreated fibers. *Cellulose* **2016**, *23*, 1777-1789, doi:10.1007/s10570-016-0897-y.
64. César, N.R.; Pereira-da-Silva, M.A.; Botaro, V.R.; de Menezes, A.J. Cellulose nanocrystals from natural fiber of the macrophyte Typha domingensis: extraction and characterization. *Cellulose* **2015**, *22*, 449-460, doi:10.1007/s10570-014-0533-7.
65. Cheng, M.; Qin, Z.; Chen, Y.; Liu, J.; Ren, Z. Facile one-step extraction and oxidative carboxylation of cellulose nanocrystals through hydrothermal reaction by using mixed inorganic acids. *Cellulose* **2017**, *24*, 3243-3254, doi:10.1007/s10570-017-1339-1.
66. Couret, L.; Irle, M.; Belloncle, C.; Cathala, B. Extraction and characterization of cellulose nanocrystals from post-consumer wood fiberboard waste. *Cellulose* **2017**, *24*, 2125-2137, doi:10.1007/s10570-017-1252-7.
67. Demewoz, G.E.; Tiruneh, A.H.; Wilson, V.H.; Jose, S.; Sundramurthy, V.P. Enhancing mechanical properties of polyvinyl alcohol films through cellulose nanocrystals derived from corncob. *Biomass Convers. Biorefin.* **2025**, *15*, 30519-30533, doi:10.1007/s13399-024-06128-6.
68. Du, H.; Liu, C.; Wang, D.; Zhang, Y.; Yu, G.; Si, C.; Li, B.; Mu, X.; Peng, H. Sustainable preparation and characterization of thermally stable and functional cellulose nanocrystals and nanofibrils via formic acid hydrolysis. *Biomed. Mater.* **2017**, *2*, doi:10.21967/jbb.v2i1.68.
69. Du, H.; Liu, C.; Mu, X.; Gong, W.; Lv, D.; Hong, Y.; Si, C.; Li, B. Preparation and characterization of thermally stable cellulose nanocrystals via a sustainable approach of

FeCl3-catalyzed formic acid hydrolysis. *Cellulose* **2016**, *23*, 2389-2407, doi:10.1007/s10570-016-0963-5.
70. Li, B.; Xu, W.; Kronlund, D.; Määttänen, A.; Liu, J.; Smått, J.-H.; Peltonen, J.; Willför, S.; Mu, X.; Xu, C. Cellulose nanocrystals prepared via formic acid hydrolysis followed by TEMPO-mediated oxidation. *Carbohydr. Polym.* **2015**, *133*, 605-612, doi:10.1016/j.carbpol.2015.07.033.
71. Ditzel, F.I.; Prestes, E.; Carvalho, B.M.; Demiate, I.M.; Pinheiro, L.A. Nanocrystalline cellulose extracted from pine wood and corncob. *Carbohydr. Polym.* **2017**, *157*, 1577-1585, doi:10.1016/j.carbpol.2016.11.036.
72. Dube, A.M.; Daba, B.J.; Muleta, M.D. Optimized isolation and characterization of cellulose for extraction of cellulose nanocrystals from Ensete ventricosum pseudo-stem fibre using a two-stage extraction method. *J. Exp. Nanosci.* **2023**, *18*, 2199989, doi:10.1080/17458080.2023.2199989.
73. Fardioui, M.; Stambouli, A.; Gueddira, T.; Dahrouch, A.; Qaiss, A.E.K.; Bouhfid, R. Extraction and characterization of nanocrystalline cellulose from doum (Chamaerops humilis) leaves: a potential reinforcing biomaterial. *J. Polym. Environ.* **2016**, *24*, 356-362, doi:10.1007/s10924-016-0784-5.
74. Fitriani, F.; Aprilia, S.; Arahman, N.; Bilad, M.R.; Amin, A.; Huda, N.; Roslan, J. Isolation and characterization of nanocrystalline cellulose isolated from pineapple crown leaf fiber agricultural wastes using acid hydrolysis. *Polymers* **2021**, *13*, 4188, doi:10.3390/polym13234188.
75. Frost, B.A.; Foster, E.J. Isolation of thermally stable cellulose nanocrystals from spent coffee grounds via phosphoric acid hydrolysis. *J. Renew. Mater.* **2020**, *8*, 187-203, doi:10.32604/jrm.2020.07940.
76. Getachew, M.; Gabriel, T.; Belete, A.; Gebre-Mariam, T. Extraction and characterization of cellulose and microcrystalline cellulose from Teff straw and evaluation of the microcrystalline cellulose as tablet excipient. *J. Nat. Fibers* **2023**, *20*, 2245565, doi:10.1080/15440478.2023.2245565.
77. Benito-González, I.; López-Rubio, A.; Gavara, R.; Martínez-Sanz, M. Cellulose nanocrystal-based films produced by more sustainable extraction protocols from Posidonia oceanica waste biomass. *Cellulose* **2019**, *26*, 8007-8024, doi:10.1007/s10570-019-02641-4.
78. Dunlop, M.J.; Acharya, B.; Bissessur, R. Isolation of nanocrystalline cellulose from tunicates. *Journal of Environmental Chemical Engineering* **2018**, *6*, 4408-4412, doi:10.1016/j.jece.2018.06.056.
79. Feng, X.; Meng, X.; Zhao, J.; Miao, M.; Shi, L.; Zhang, S.; Fang, J. Extraction and preparation of cellulose nanocrystals from dealginate kelp residue: structures and morphological characterization. *Cellulose* **2015**, *22*, 1763-1772, doi:10.1007/s10570-015-0617-z.
80. Hachaichi, A.; Kouini, B.; Kian, L.K.; Asim, M.; Fouad, H.; Jawaid, M.; Sain, M. Nanocrystalline cellulose from microcrystalline cellulose of date palm fibers as a promising candidate for bio-nanocomposites: isolation and characterization. *Materials* **2021**, *14*, 5313, doi:10.3390/ma14185313.
81. Van Hai, L.; Son, H.N.; Seo, Y.B. Physical and bio-composite properties of nanocrystalline cellulose from wood, cotton linters, cattail, and red algae. *Cellulose* **2015**, *22*, 1789-1798, doi:10.1007/s10570-015-0633-z.

82. Han, W.; Geng, Y. Optimization and characterization of cellulose extraction from olive pomace. *Cellulose* **2023**, *30*, 4889-4903, doi:10.1007/s10570-023-05195-8.
83. Ilyas, R.; Sapuan, S.; Ishak, M. Isolation and characterization of nanocrystalline cellulose from sugar palm fibres (Arenga Pinnata). *Carbohydr. Polym.* **2018**, *181*, 1038-1051, doi:10.1016/j.carbpol.2017.11.045.
84. Ioelovich, M. Optimization of production process of nanocrystalline cellulose and its composites. *Russian Journal of Bioorganic Chemistry* **2022**, *48*, 1411-1415, doi:10.1134/S106816202207007X.
85. Islam, M.S.; Kao, N.; Bhattacharya, S.N.; Gupta, R. Optimization and modelling of delignification process for nanocrystalline cellulose production from rice husk biomass. *AIP Conference Proceedings* **2017**, *1914*, doi:10.1063/1.5016692.
86. Islam, M.S.; Kao, N.; Bhattacharya, S.N.; Gupta, R. An investigation between high and low pressure processes for nanocrystalline cellulose production from agro-waste biomass. *AIP Conference Proceedings* **2017**, *1914*, doi:10.1063/1.5016729.
87. Jahan, M.S.; Hossain, S.; Das, S.C.; Rahman, M.L.; Khan, M.A. Extraction and characterization of cellulose nanocrystals (CNCs) from Jute fibers and other by-products towards a circular economy approach. *Mater. Circ. Econ.* **2024**, *6*, 57, doi:10.1007/s42824-024-00149-2.
88. Jordan, J.H.; Easson, M.W.; Dien, B.; Thompson, S.; Condon, B.D. Extraction and characterization of nanocellulose crystals from cotton gin motes and cotton gin waste. *Cellulose* **2019**, *26*, 5959-5979, doi:10.1007/s10570-019-02533-7.
89. Joshi, M.; Manjare, S. Sugarcane trash-derived nanocrystalline cellulose: a sustainable approach with mild chemical pretreatment and comprehensive characterisation. *Cellulose* **2024**, *31*, 4947-4965, doi:10.1007/s10570-024-05906-9.
90. Karakehya, N.; Bilgiç, C. Preparation of nanocrystalline cellulose from tomato stem and commercial microcrystalline cellulose: A comparison between two starting materials. *Cellul. Chem. Technol.* **2019**, *53*, 993-1000, doi:10.35812/CelluloseChemTechnol.2019.53.97.
91. Dinçel Kasapoğlu, E.; Kahraman, S.; Tornuk, F. Extraction optimization and characterization of cellulose nanocrystals from apricot pomace. *Foods* **2023**, *12*, 746, doi:10.3390/foods12040746.
92. Khan, A.; Jawaid, M.; Kian, L.K.; Khan, A.A.P.; Asiri, A.M. Isolation and production of nanocrystalline cellulose from conocarpus fiber. *Polymers* **2021**, *13*, 1835, doi:10.3390/polym13111835.
93. Kian, L.K.; Jawaid, M.; Ariffin, H.; Karim, Z. Isolation and characterization of nanocrystalline cellulose from roselle-derived microcrystalline cellulose. *Int. J. Biol. Macromol.* **2018**, *114*, 54-63, doi:10.1016/j.ijbiomac.2018.03.065.
94. Kian, L.K.; Saba, N.; Jawaid, M.; Alothman, O.Y.; Fouad, H. Properties and characteristics of nanocrystalline cellulose isolated from olive fiber. *Carbohydr. Polym.* **2020**, *241*, 116423, doi:10.1016/j.carbpol.2020.116423.
95. Kumar, P.; Miller, K.; Kermanshahi-pour, A.; Brar, S.K.; Beims, R.F.; Xu, C.C. Nanocrystalline cellulose derived from spruce wood: Influence of process parameters. *Int. J. Biol. Macromol.* **2022**, *221*, 426-434, doi:10.1016/j.ijbiomac.2022.09.017.
96. Lee, B.-M.; Jeun, J.-P.; Kang, P.-H.; Choi, J.-H.; Hong, S.-K. Isolation and characterization of nanocrystalline cellulose from different precursor materials. *Fibers Polym.* **2017**, *18*, 272-277, doi:10.1007/s12221-017-6548-6.

97. Li, Z.; Liu, A.; Wu, H.; Naeem, A.; Fan, Q.; Jin, Z.; Liu, H.; Ming, L. Extraction of cellulose nanocrystalline from Camellia oleifera Abel waste shell: Study of critical processes, properties and enhanced emulsion performance. *Int. J. Biol. Macromol.* **2024**, *254*, 127890, doi:10.1016/j.ijbiomac.2023.127890.
98. Liu, C.; Du, H.; Yu, G.; Zhang, Y.; Kong, Q.; Li, B.; Mu, X. Simultaneous extraction of carboxylated cellulose nanocrystals and nanofibrils via citric acid hydrolysis—A sustainable route. *Pap. Biomater.* **2017**, *2*, 19–26, doi:10.26599/PBM.2017.9260024.
99. Liu, Z.; Li, X.; Xie, W.; Deng, H. Extraction, isolation and characterization of nanocrystalline cellulose from industrial kelp (Laminaria japonica) waste. *Carbohydr. Polym.* **2017**, *173*, 353-359, doi:10.26599/PBM.2017.9260024.
100. Luzi, F.; Fortunati, E.; Puglia, D.; Petrucci, R.; Kenny, J.M.; Torre, L. Modulation of acid hydrolysis reaction time for the extraction of cellulose nanocrystals from Posidonia oceanica leaves. *J. Renew. Mater.* **2016**, *4*, 190–198, doi:10.7569/JRM.2015.634134.
101. Mamudu, U.; Kabyshev, A.; Bekmyrza, K.; Kuterbekov, K.A.; Baratova, A.; Omeiza, L.A.; Lim, R.C. Extraction, preparation and characterization of nanocrystalline cellulose from lignocellulosic Simpor leaf residue. *Molecules* **2025**, *30*, 1622, doi:10.3390/molecules30071622.
102. Melenia, A.T.; Jovita, S.; Utami, D.I.; Tamim, R.; Holilah, H.; Bahruji, H.; Hamid, Z.A.A.; Mubarok, F.; Widiyastuti, W.; Wibisono, A.T.; et al. Nanocrystalline cellulose from Calophyllum inophyllum shells waste by adjusting organic acid hydrolysis and optimization of reaction parameters using response surface methodology. *Int. J. Biol. Macromol.* **2024**, *281*, 135705, doi:10.1016/j.ijbiomac.2024.135705
103. Melikoğlu, A.Y.; Bilek, S.E.; Cesur, S. Optimum alkaline treatment parameters for the extraction of cellulose and production of cellulose nanocrystals from apple pomace. *Carbohydr. Polym.* **2019**, *215*, 330-337, doi:10.1016/j.carbpol.2019.03.103.
104. Mendes, C.A.d.C.; Ferreira, N.M.S.; Furtado, C.R.G.; de Sousa, A.M.F. Isolation and characterization of nanocrystalline cellulose from corn husk. *Mater. Lett.* **2015**, *148*, 26-29, doi:10.1016/j.matlet.2015.02.047.
105. Mishra, S. New excipient for oral drug delivery: CNC derived from sugarcane bagasse-derived microcrystalline cellulose. *ACS Omega* **2024**, *9*, 19353-19362, doi:10.1021/acsomega.4c00497.
106. Musa, A.; Ahmad, M.B.; Hussein, M.Z.; Izham, S.M. Acid hydrolysis-mediated preparation of nanocrystalline cellulose from rice straw. *Int. J. Nanomater. Nanotechnol. Nanomed.* **2017**, *3*, 051–056, doi:10.17352/2455-3492.000021.
107. Nsude, O.P.; Orie, K.J. Nano-crystalline cellulose of African bean (Pentaclethra macrophylla Benth) pod: extraction and characterisation. *J. Chem. Soc. Niger.* **2023**, *48*, doi:10.46602/jcsn.v48i4.905.
108. Nurdiana, O.; Sam, S.T.; Faiq, A.M. Optimization of the product of nanocrystalline cellulose from coconut husks. *IOP Conference Series: Materials Science and Engineering* **2018**, *429*, 012041, doi:10.1088/1757-899X/429/1/012041.
109. Nuruddin, A.; Habibullah, A.; Adipratama, M.J.; Purwasasmita, B.S. Synthesis of barium hexaferrite templated by nanocrystalline cellulose extracted from luffa acutangula fiber. *Mater. Res. Express* **2021**, *8*, 066104, doi:10.1088/2053-1591/abe5fe.
110. Nwosu, O.U.; Nwanonenyi, S.C.; Osuagwu, C.O.; Nnawugwu, N.; Eziefula, U.G.; Ononogbo, C. Extraction and characterization of nanocrystalline cellulose from

agricultural residue—oil palm petiole fiber. *Int. J. Adv. Sci. Eng.* **2022**, *9*, 2751–2757, doi:10.29294/IJASE.9.2.2022.2751-2757.

111. Seta, F.T.; An, X.; Liu, L.; Zhang, H.; Yang, J.; Zhang, W.; Nie, S.; Yao, S.; Cao, H.; Xu, Q.; et al. Preparation and characterization of high yield cellulose nanocrystals (CNC) derived from ball mill pretreatment and maleic acid hydrolysis. *Carbohydr. Polym.* **2020**, *234*, 115942, doi:10.1016/j.carbpol.2020.115942.
112. Pandi, N.; Sonawane, S.H.; Anand Kishore, K. Synthesis of cellulose nanocrystals (CNCs) from cotton using ultrasound-assisted acid hydrolysis. *Ultrason. Sonochem.* **2021**, *70*, 105353, doi:10.1016/j.ultsonch.2020.105353
113. Rahman, M.M.; Hossain, M.I.; Hosen Pk, M.E.; Al-amin, M.; Knani, S.; Waliullah, M.; Gafur, M.A.; Ghos, B.C. Production of cellulose nanocrystal (CNC) from papaya tree stem as a potential reinforcement in bionanocomposite manufacturing. *Bioresour. Technol. Rep.* **2025**, *31*, 102284, doi:10.1016/j.biteb.2025.102284.
114. Pk, M.E.H.; Rahman, M.M.; Ghos, B.C.; Ahmed, N.U.; Hossen, M.N.; Knani, S.; Rahman, M.S.; Hossain, M.I. Production of cellulose nanocrystals from the residual mass of P. chaba stem after methanol extraction as potential reinforcement in sustainable nanocomposite manufacturing: A cutting-edge approach in waste management. *Bioresour. Technol. Rep.* **2025**, *31*, 102273, doi:10.1016/j.biteb.2025.102273.
115. Rashid, S.; Shahi, A.K.; Dutta, H.; Sahu, J.K. Extraction and characterization of cellulose and cellulose nanowhiskers from almond shell biomass, metal removal and toxicity analysis. *Biointerface Res. Appl. Chem.* **2022**, *12*, 1705–1720, doi:10.33263/BRIAC122.17051720.
116. Mohd Rasli, S.R.A.; Ahmad, I.; Mat Lazim, A.; Hamzah, A. Extraction and characterization of cellulose from agricultural residue—oil palm fronds. *Malays. J. Anal. Sci.* **2017**, *21*, 1065–1073, doi:10.17576/mjas-2017-2105-08.
117. Ratna, R.; Arahman, N.; Munawar, A.A.; Aprilia, S. Extraction, isolation, and characterization of nanocrystalline cellulose from barangan banana (Musa acuminata L.) peduncles waste. *Indones. J. Chem.* **2023**, *23*, 73–89, doi:10.22146/ijc.74718.
118. Ratna; Aprilia, S.; Arahman, N.; Munawar, A.A. Characterization of cellulose nanocrystalline isolated from banana peduncles using acid hydrolysis. *IOP Conf. Ser.: Earth Environ. Sci.* **2021**, *922*, 012072, doi:10.1088/1755-1315/922/1/012072.
119. Ritonga, S.I.; Frida, E.; Humaidi, S. Extraction and characterization of nanocrystalline cellulose from sugar palm fibres (Arenga pinnata). *Prisma Sains* **2023**, *11*, 612-618, doi:10.33394/j-ps.v11i2.7935.
120. Rovera, C.; Carullo, D.; Bellesia, T.; Büyüktaş, D.; Ghaani, M.; Caneva, E.; Farris, S. Extraction of high-quality grade cellulose and cellulose nanocrystals from different lignocellulosic agri-food wastes. *Front. Sustain. Food Syst.* **2023**, *Volume 6 - 2022*, doi:10.3389/fsufs.2022.1087867.
121. Rusdianto, A.S.; Amilia, W.; Sinta, V.J.D. The optimization of cellulose content in tobacco stems (Nicotiana tabaccum L.) with acid extraction method and alkaline extraction method. *Int. J. Food Agric. Nat. Resour.* **2021**, *2*, 13–19, doi:10.46676/ij-fanres.v2i2.28.
122. Rusdin, A.; Ahmad, A.; Karim, A.; Kasim, S.; Wahab, A.W.; Tahir, D.; Rohaeti, E.; Taba, P.; Awaluddin; Baharuddin, M.; et al. Comparison of extraction methods and characterization of cellulose from sugarcane bagasse (Saccharum officinarum L.) for bioplastic food packaging. *Tropical Journal of Natural Product Research* **2024**, *8*, doi:10.26538/tjnpr/v8i12.42.

123. Samarawickrama, K.G.R.; Wijayapala, U.G.S.; Fernando, C.A.N. Extraction and analysis of cellulose nanocrystals from cotton balls by acid hydrolysis. *Journal of the University of Kelaniya* **2023**, *16*, 23–29, doi:10.4038/josuk.v16i1.8075.
124. Saupi, F.A.C.; Yusof, N.S.M.; Rajagopal, V.; Rahman, N.M.M.A. Comparative analysis of nanocellulose extraction from Cocos nucifera shell by ultrasonication versus acid hydrolysis for methylene blue dye removal. *Iran. Polym. J.* **2025**, *34*, 1755-1778, doi:10.1007/s13726-025-01462-6.
125. Kusmono; Listyanda, R.F.; Wildan, M.W.; Ilman, M.N. Preparation and characterization of cellulose nanocrystal extracted from ramie fibers by sulfuric acid hydrolysis. *Heliyon* **2020**, *6*, doi:10.1016/j.heliyon.2020.e05486.
126. Nguyen, T.T.; Nguyen, H.C.; Chu, T.H.; Le, Q.D. Preparation and characterizations of nanocellulose from wood pulp using hydrolysis method. *Old Journal* **2019**, 11-14.
127. Wu, Y.; Luo, C.; Wang, T.; Yang, Y.; Sun, Y.; Zhang, Y.; Cui, L.; Song, Z.; Chen, X.; Cao, X.; et al. Extraction and characterization of nanocellulose from cattail leaves: Morphological, microstructural and thermal properties. *Int. J. Biol. Macromol.* **2024**, *255*, 128123, doi:10.1016/j.ijbiomac.2023.128123.
128. Yu, H.-Y.; Zhang, D.-Z.; Lu, F.-F.; Yao, J. New approach for sngle-step extraction of carboxylated cellulose nanocrystals for their use as adsorbents and flocculants. *ACS Sustainable Chemistry & Engineering* **2016**, *4*, 2632-2643, doi:10.1021/acssuschemeng.6b00126.
129. Serrano-Martínez, V.M.; Pérez-Aguilar, H.; Carbonell-Blasco, M.P.; García-García, A.; Arán-Ais, F.; Orgilés-Calpena, E. Effect of acid hydrolysis conditions on the extraction of cellulose nanocrystals. *Polymers* **2025**, *17*, 1313, doi:10.3390/polym17101313.
130. Murugesan, S.; Rajasree, S.R.R.; Rajan, R. Extraction and characterization of cellulose nanocrystals from brown seaweed Dictyota bartayresiana, J.V. Lamouroux. *J. Cluster Sci.* **2024**, *36*, 12, doi:10.1007/s10876-024-02721-x.
131. Tenaye, T.; Mohammed, S.A.; Jabasingh, S.A. Sustainable synthesis and characterization of Enset cellulose nanocrystals (E-CNp) from Enset ventricosum biomass and its application in the fabrication of Enset cellulose nanocomposite (E-CNc). *Biomass Convers. Biorefin.* **2024**, *14*, 5019-5034, doi:10.1007/s13399-022-02682-z.
132. Travalini, A.P.; Prestes, E.; Pinheiro, L.A.; Demiate, I.M. Extraction and characterization of nanocrystalline cellulose from cassava bagasse. *J. Polym. Environ.* **2018**, *26*, 789-797, doi:10.1007/s10924-017-0983-8.
133. Wibowo, A.; Chiarasumran, N.; Thanapimmetha, A.; Saisriyoot, M.; Srinophakun, P.; Suriyachai, N.; Champreda, V. Conversion of sugarcane trash to nanocrystalline cellulose and its life cycle assessment. *Catalysts* **2022**, *12*, 1215, doi:10.3390/catal12101215.
134. Ferreira, F.V.; Mariano, M.; Rabelo, S.C.; Gouveia, R.F.; Lona, L.M.F. Isolation and surface modification of cellulose nanocrystals from sugarcane bagasse waste: From a micro- to a nano-scale view. *Appl. Surf. Sci.* **2018**, *436*, 1113-1122, doi:10.1016/j.apsusc.2017.12.137.
135. Woldie, W.A.; Shibeshi, N.T.; Kuffi, K.D. Optimization of cellulose nanocrystals extraction from teff straw using acid hydrolysis followed by ultrasound sonication. *Carbohydr. Polym. Technol. Appl.* **2025**, *9*, 100707, doi:10.1016/j.carpta.2025.100707.
136. Dyah Kencana Wulan, P.P.; Ismojo; Khumaeroh; Syabila, A.N.; Handayani, A.S.; Ratnawati. Sustainable extraction of cellulose nanocrystals from empty palm oil bunches

via low-acid hydrolysis. *Results Eng.* **2024**, *24*, 103012, doi:10.1016/j.rineng.2024.103012.
137. Yu, X.; Jiang, Y.; Wu, Q.; Wei, Z.; Lin, X.; Chen, Y. Preparation and characterization of cellulose nanocrystal extraction from Pennisetum hydridum fertilized by municipal sewage Sludge via sulfuric acid hydrolysis. *Front. Energy Res.* **2021**, *Volume 9 - 2021*, doi:10.3389/fenrg.2021.774783.
138. Romadhon, Z.R.F.; Made Sanjaya, I.G. Development of nanocomposite films with durian peel nanocrystalline cellulose. *Jurnal Akademika Kimia* **2021**, *10*, 230-236, doi:10.22487/j24775185.2021.v10.i4.pp230-236.
139. Zhang, H.; Chen, Y.; Wang, S.; Ma, L.; Yu, Y.; Dai, H.; Zhang, Y. Extraction and comparison of cellulose nanocrystals from lemon (Citrus limon) seeds using sulfuric acid hydrolysis and oxidation methods. *Carbohydr. Polym.* **2020**, *238*, 116180, doi:https://doi.org/10.1016/j.carbpol.2020.116180.
140. Sun, B.; Zhang, M.; Hou, Q.; Liu, R.; Wu, T.; Si, C. Further characterization of cellulose nanocrystal (CNC) preparation from sulfuric acid hydrolysis of cotton fibers. *Cellulose* **2016**, *23*, 439-450, doi:10.1007/s10570-015-0803-z.
141. Zhong, Y.; Gao, X.; Zhang, W.; Wang, X.; Wang, K. Carboxylated nanocrystalline cellulose from Salix psammophila prepared by sulfuric acid hydrolysis combined with TEMPO-oxidation. *BioResources* **2022**, *17*, 1373–1384, doi:10.15376/biores.17.1.1373-1384.
142. Zhu, Z.; Wang, W.; Wang, X.; Zhao, X.; Xia, N.; Kong, F.; Wang, S. Easy way to prepare dispersible CNC dry powder by precipitation and conventional evaporation. *Cellulose* **2021**, *28*, 9661-9676, doi:10.1007/s10570-021-04123-y.
143. Zulnazri, Z.; Dewi, R.; Muarif, A.; Fikri, A.; Fithra, H.; Roesyadi, A.; Sangian, H.F.; Alva, S. Effect of hydrochloric acid hydrolysis under sonication and hydrothermal process to produce cellulose nanocrystals from oil palm empty fruit bunch (OPEFB). *Polymers* **2024**, *16*, 1866, doi:10.21203/rs.3.rs-3062011/v1.
144. Gallardo-Sánchez, M.A.; Diaz-Vidal, T.; Navarro-Hermosillo, A.B.; Figueroa-Ochoa, E.B.; Ramirez Casillas, R.; Anzaldo Hernández, J.; Rosales-Rivera, L.C.; Soltero Martínez, J.F.A.; García Enríquez, S.; Macías-Balleza, E.R. Optimization of the obtaining of cellulose nanocrystals from Agave tequilana Weber Var. azul bagasse by acid hydrolysis. *Nanomaterials* **2021**, *11*, 520, doi:10.3390/nano11020520.
145. Tan, X.; Peng, Q.; Yang, K.; Yang, T.; Saskova, J.; Wiener, J.; Venkataraman, M.; Militky, J.; Xiong, W.; Xu, J. Preparation and Characterization of corn husk nanocellulose coating on electrospun polyamide 6. *Alexandria Eng. J.* **2022**, *61*, 4529-4540, doi:10.1016/j.aej.2021.10.011.
146. Evdokimova, O.L.; Alves, C.S.; Krsmanović Whiffen, R.M.; Ortega, Z.; Tomás, H.; Rodrigues, J. Cytocompatible cellulose nanofibers from invasive plant species Agave americana L. and Ricinus communis L.: a renewable green source of highly crystalline nanocellulose. *J. Zhejiang Univ.-Sci. B* **2021**, *22*, 450-461, doi:10.1631/jzus.B2000683.
147. Chen, Y.W.; Lee, H.V.; Juan, J.C.; Phang, S.-M. Production of new cellulose nanomaterial from red algae marine biomass Gelidium elegans. *Carbohydr. Polym.* **2016**, *151*, 1210-1219, doi:10.1016/j.carbpol.2016.06.083.
148. James, A.; Johannes, M.S.; Fanyana, M.F.; Ojo, F.; Bamidele, O.J. Extraction and characterization of α-cellulose-rich residue from maize (Zea mays L.) husk. *Cellul. Chem. Technol.* **2023**, *57*, 935–944, doi:http://dx.doi.org/10.1016/j.foodchem.2016.10.048.

149. Oliveira, J.P.d.; Bruni, G.P.; Lima, K.O.; Halal, S.L.M.E.; Rosa, G.S.d.; Dias, A.R.G.; Zavareze, E.d.R. Cellulose fibers extracted from rice and oat husks and their application in hydrogel. *Food Chem.* **2017**, *221*, 153-160, doi:10.1016/j.foodchem.2016.10.048.
150. Sigma Aldrich. *Safety Data Sheet: Sodium hydroxide (S8045)*; Version 7.3 — Revision Date 11 September 2025; 2025.
151. Tata Chemicals Limited. *Safety Data Sheet: Sodium Hydroxide*; Updated as on 21 June 2021; 2021.
152. Thermo Fisher Scientific. *Safety Data Sheet: Sodium Hydroxide (CAS No. 1310-73-2)*; 2023.
153. AkKim Kimya Sanayi. *Safety Data Sheet: Sodium Hydroxide*; 2020.
154. FUJIFILM Wako Pure Chemical Corporation. *Safety Data Sheet: Sodium Hydroxide*; FUJIFILM Wako Pure Chemical Corporation: Osaka, Japan, 2024.
155. Sigma Aldrich. *Safety Data Sheet: Potassium hydroxide (P5958)*; Version 6.10 — Revision Date 18 December 2024; 2024.
156. Carl Roth GmbH. *Safety Data Sheet: Potassium Hydroxide ≥85%, p.a., Pellets (Article 6751)*; ersion 6.0 — Revision Date 18 September 2024; 2024.
157. Nixon, G.A.; Bannan, E.A.; Gaynor, T.W.; Johnston, D.H.; Griffith, J.F. Evaluation of modified methods for determining skin irritation. *Regul Toxicol Pharmacol* **1990**, *12*, 127-136, doi:10.1016/s0273-2300(05)80054-6.
158. Kishida Chemical Co. *Safety Data Sheet: Potassium hydroxide*; Kishida Chemical Co., Ltd.: Osaka, Japan, 2024.
159. Columbus Chemical Industries Inc. *Safety Data Sheet: Potassium hydroxide 45%*; Columbus Chemical Industries, Inc: Columbus, WI, USA, 2025.
160. FUJIFILM Wako Pure Chemical Corporation. *Safety Data Sheet: 1 mol/L Potassium hydroxide solution*; W01W0116-0388 JGHEEN; FUJIFILM Wako Pure Chemical Corporation: Osaka, Japan, 2024.
161. Sigma Aldrich. *Safety Data Sheet: Sodium chlorite (244155)*; Version 6.7 — Revision Date 16 October 2024; 2024.
162. Thermo Fisher Scientific. *Safety Data Sheet: Sodium chlorite, unstabilized*; Revision 8 — Revision Date 17 June 2025; 2025.
163. DC Fine Chemicals. *Safety Data Sheet: Sodium chlorite 80% (Product Code 123770)*; Terrassa, Spain, 2024.
164. FUJIFILM Wako Pure Chemical Corporation. *Safety Data Sheet: Chlorite ion standard solution (ClO_2^- 1000)*; FUJIFILM Wako Pure Chemical Corporation: Osaka, Japan, 2023.
165. Sigma Aldrich. *Safety Data Sheet: Sodium chlorite (25% solution in water)*; 2024.
166. Sigma Aldrich. *Safety Data Sheet: Sodium hypochlorite solution (425044)*; Version 7.3 — Revision Date 10 July 2024; 2024.
167. Thermo Fisher Scientific. *Safety Data Sheet: Sodium hypochlorite, 11–15% Available Chlorine (33369)*; Revision 7 — Revision Date 31 December 2024; 2024.
168. Kim, J.; Park, C.M.; Choi, S.H.; Yang, M.J.; Lee, J.Y.; Jeon, B.S.; Ku, H.O.; Kim, M.S. Assessment of acute inhalation toxicity of citric acid and sodium hypochlorite in rats. *J. Vet. Sci.* **2023**, *24*, e22, doi:10.4142/jvs.22253.
169. FUJIFILM Wako Pure Chemical Corporation. *Safety Data Sheet: Sodium hypochlorite solution*; FUJIFILM Wako Pure Chemical Corporation: Osaka, Japan, 2025.
170. Carl Roth GmbH. *Safety Data Sheet: Hydrogen peroxide 35% pure, stabilized (article 9683)*; Version 8.1 — Revision Date 10 October 2024; 2024.

171. Sigma Aldrich. *Safety Data Sheet: Hydrogen peroxide solution (16911)*; Version 6.11 — Revision Date 3 September 2024; 2024.
172. Thermo Fisher Scientific. *Safety Data Sheet: Hydrogen peroxide (30% in water)*; Revision 12 — Revision Date 24 December 2021; 2021.
173. FUJIFILM Wako Pure Chemical Corporation. *Safety Data Sheet: Hydrogen peroxide*; FUJIFILM Wako Pure Chemical Corporation: Osaka, Japan, 2025.
174. Sigma Aldrich. *Safety Data Sheet: Formic Acid (F0507)*; 2024.
175. Department of the Environment, W., Heritage and the Arts,; Department of the Environment and Energy. *Formic Acid: Environmental Assessment Dossier*; 2021.
176. Merck KGaA. *Safety Data Sheet: Formic Acid 98–100% for Analysis EMSURE® ACS, Reag. Ph. Eur.*; Darmstadt, Germany, 2021.
177. FUJIFILM Wako Pure Chemical Corporation. *Safety Data Sheet: Formic acid*; FUJIFILM Wako Pure Chemical Corporation: Osaka, Japan, 2024.
178. Sigma Aldrich. *Safety Data Sheet: Lactic acid (Product Number W261106)*; 2025.
179. Thermo Fisher Scientific. *Safety Data Sheet: DL-Lactic acid (Cat. No. A159-500)*; 2024.
180. Jungbunzlauer Suisse AG. *Safety Data Sheet: L(+)-Lactic acid 88%*; Jungbunzlauer Suisse AG: Basel, Switzerland, 2021.
181. Sigma Aldrich. *Safety Data Sheet: Citric acid (Product No. 251275)*; 2025.
182. Guerrette, Z.; Rutkiewicz, J. *GreenScreen® for safer chemicals: Citric acid (CAS No. 77-92-9), GS-176*; 2024.
183. European Directorate for the Quality of Medicines & Healthcare. *Safety Data Sheet: Citric acid (anhydrous)*; 2023.
184. FUJIFILM Wako Pure Chemical Corporation. *Safety Data Sheet: Citric acid Monohydrate*; FUJIFILM Wako Pure Chemical Corporation: Osaka, Japan, 2025.
185. Sigma Aldrich. *Safety Data Sheet: Hydrochloric acid (Product No. H1758)*; 2025.
186. Thermo Fisher Scientific. *Safety Data Sheet: Hydrochloric acid*; 2023.
187. GHC Gerling Holz & Co Handels GmbH. *Safety Data Sheet: Hydrogen chloride (CAS 7647-01-0)*; 2024.
188. FUJIFILM Wako Pure Chemical Corporation. *Safety Data Sheet: 30% Hydrochloric acid*; FUJIFILM Wako Pure Chemical Corporation: 2024.
189. Sigma Aldrich. *Safety Data Sheet: Sulfuric acid (Product No. 339741)*; 2024.
190. Thermo Fisher Scientific. *Safety Data Sheet: Sulfuric acid*; 2021.
191. Carl Roth GmbH. *Safety Data Sheet: Sulphuric acid ROTIPURAN® 96 %, p.a., ISO*; 2025.
192. Spectrum Chemical Manufacturing Corporation. *Safety Data Sheet: Sulfuric acid, 66° Bé, Technical*; Gardena, California, USA, 2021.
193. FUJIFILM Wako Pure Chemical Corporation. *Safety Data Sheet: 1 mol/L Sulfuric acid*; FUJIFILM Wako Pure Chemical Corporation: FUJIFILM Wako Pure Chemical Corporation, 2024.
194. Sigma Aldrich. *Safety Data Sheet: Phosphoric acid (product No. P6560)*; 2022.
195. Carl Roth GmbH. *Safety Data Sheet: Ortho-phosphoric acid 85 %, extra pure (article No. 9079)*; 2024.
196. National Industrial Chemicals Notification and Assessment Scheme (NICNAS); Australian Government Department of Health; Australian Government Department of the Environment and Energy. *Phosphoric acid: human health tier II assessment*; 2016.
197. Nutrient Ltd. *Safety Data Sheet: Phosphoric acid 85% Food Grade*; 2021.

198. FUJIFILM Wako Pure Chemical Corporation. *Safety Data Sheet: Phosphoric acid*; FUJIFILM Wako Pure Chemical Corporation: Osaka, Japan, 2025.
199. World Health Organization. *Sodium in drinking-water: background document for development of WHO guidelines for drinking water quality*; 2003.
200. Mount, D.R.; Erickson, R.J.; Highland, T.L.; Hockett, J.R.; Hoff, D.J.; Jenson, C.T.; Norberg-King, T.J.; Peterson, K.N.; Polaske, Z.M.; Wisniewski, S. The acute toxicity of major ion salts to Ceriodaphnia dubia: I. influence of background water chemistry. *Environ. Toxicol. Chem.* **2016**, *35*, 3039-3057, doi:10.1002/etc.3487.
201. United State Environmental Protection Agency. National Primary Drinking Water Regulations. Available online: https://www.epa.gov/ground-water-and-drinking-water/national-primary-drinking-water-regulations (accessed on 20 Dec 2025).
202. Agency for Toxic Substances and Disease Registry. *Public Health Statement: Chlorine dioxide and chlorite*; 2004.
203. Fisher, D.J.; Burton, D.T.; Yonkos, L.T.; Turley, S.D.; Ziegler, G.P.; Turley, B.S. Derivation of acute ecological risk criteria for chlorite in freshwater ecosystems. *Water Res.* **2003**, *37*, 4359-4368, doi:10.1016/S0043-1354(03)00433-0.
204. Canada, H. Guidelines for Canadian drinking water quality, guideline technical document - chlorite and chlorate **2008**, 1-53.
205. World Health Organization. *Chlorine dioxide, chlorite and chlorate in drinking-water: background document for development of WHO guidelines for drinking water quality*; 2016.
206. Svecevičius, G.; Šyvokienė, J.; Stasiūnaitė, P.; Mickėnienė, L. Acute and chronic toxicity of chlorine dioxide (ClO2) and chlorite (ClO2) to rainbow trout (Oncorhynchus mykiss)(4 pp). *Environ. Sci. Pollut. Res.* **2005**, *12*, 302-305, doi:10.1065/espr2005.04.248.
207. Taylor, P.A. An Evaluation of the Toxicity of Various Forms of Chlorine to Ceriodaphnia-Dubia. *Environ. Toxicol. Chem.* **1993**, *12*, 925-930, doi:10.1002/etc.5620120517.
208. Australian and New Zealand Environment and Conservation Council; Agriculture and Resource Management Council of Australia and New Zealand. *Chlorine in freshwater and marine water: toxicant default guideline values for protecting aquatic ecosystems*; 2000.
209. Michigan Department of Environment Great Lakes and Energy. *Chloride and sulfate water quality values: implementation plan*; 2021.
210. Canadian Council of Ministers of the Environment. *Scientific criteria document for the development of the Canadian water quality guidelines for the protection of aquatic lLife: Chloride Ion*; 2011.
211. Wang, N.; Dorman, R.A.; Ivey, C.D.; Soucek, D.J.; Dickinson, A.; Kunz, B.K.; Steevens, J.A.; Hammer, E.J.; Bauer, C.R. Acute and chronic toxicity of sodium nitrate and sodium sulfate to several freshwater organisms in water-only exposures. *Environ. Toxicol. Chem.* **2020**, *39*, 1071-1085, doi:10.1002/etc.4701.
212. Kim, E.; Yoo, S.; Ro, H.Y.; Han, H.J.; Baek, Y.W.; Eom, I.C.; Kim, H.M.; Kim, P.; Choi, K. Aquatic toxicity assessment of phosphate compounds. *Environ. Health Toxicol.* **2013**, *28*, e2013002, doi:10.5620/eht.2013.28.e2013002.
213. Global Safety Management. *Safety Data Sheet: Sodium phosphate, 0.1M*; Hanover, PA, 2015.
214. Qatar General Electricity and Water Corporation (KAHRAMAA). *KAHRAMAA drinking water quality requirements*; 2014.

215. Central Pollution Control Board. WHO guidelines for drinking water quality. Available online: https://cpcb.nic.in/who-guidelines-for-drinking-water-quality/ (accessed on 10 Nov 2025).
